# KKANs: Ku̇rková-Kolmogorov-Arnold networks and their learning dynamics

**DOI:** 10.1016/j.neunet.2025.107831

**Published:** 2025-07-06

**Authors:** Juan Diego Toscano, Li-Lian Wang, George Em Karniadakis

**Affiliations:** a Division of Applied Mathematics, Brown University, Providence, 02912, RI, USA; b Division of Mathematical Sciences, Nanyang Technological University, 637371, Singapore

**Keywords:** Kolmogorov-Arnold representation theorem, Physics-informed neural networks, Kolmogorov-Arnold networks, Optimization algorithms, Self-adaptive weights, Information bottleneck theory

## Abstract

Inspired by the Kolmogorov-Arnold representation theorem and Ku̇rková’s principle of using approximate representations, we propose the Ku̇rková-Kolmogorov-Arnold Network (KKAN), a new two-block architecture that combines robust multi-layer perceptron (MLP) based inner functions with flexible linear combinations of basis functions as outer functions. We first prove that KKAN is a universal approximator, and then we demonstrate its versatility across scientific machine-learning applications, including function regression, physics-informed machine learning (PIML), and operator-learning frameworks. The benchmark results show that KKANs outperform MLPs and the original Kolmogorov-Arnold Networks (KANs) in function approximation and operator learning tasks and achieve performance comparable to fully optimized MLPs for PIML. To better understand the behavior of the new representation models, we analyze their geometric complexity and learning dynamics using information bottleneck theory, identifying three universal learning stages, fitting, transition, and diffusion, across all types of architectures. We find a strong correlation between geometric complexity and signal-to-noise ratio (SNR), with optimal generalization achieved during the diffusion stage. Additionally, we propose self-scaled residual-based attention weights to maintain high SNR dynamically, ensuring uniform convergence and prolonged learning.

## 1. Introduction

Scientific machine learning (SciML) has emerged as a transformative approach for solving complex scientific problems by integrating machine learning with domain-specific knowledge from physics, biology, engineering, finance, and beyond (Toscano et al., 2025b). Within this framework, methods such as Physics-Informed Machine Learning (PIML) (Raissi et al., 2019a) and Operator Learning (Lu et al., 2019) have gained significant attention due to their ability to merge the predictive power of machine learning with the foundational principles of physics (Toscano et al., 2025b). These approaches rely on data-efficient and physics-guided learning to model systems that are otherwise difficult to solve using traditional methods (Boster et al., 2023; Cai et al., 2021; Raissi et al., 2020; Toscano et al., 2025a, 2024).

A cornerstone of SciML is the Multilayer Perceptron (MLP) used as the primary model architecture (Karniadakis et al., 2021). MLPs are foundational in modern deep learning (Goodfellow et al., 2020; He et al., 2016; Kaelbling et al., 1996; Li et al., 2018; Poluektov & Polar, 2020; Toscano et al., 2023; Vaswani et al., 2017) due to their proven ability to approximate complex functions, as guaranteed by the universal approximation theorem (Park & Sandberg, 1991). Despite their widespread success, MLPs face notable challenges, including limited interpretability (Cranmer, 2023), susceptibility to overfitting, and scalability issues (Jagtap & Karniadakis, 2020; Jagtap et al., 2020b). Addressing these limitations has become a central focus of recent advancements in SciML frameworks (Jagtap et al., 2020a; McClenny & Braga-Neto, 2023; Wang et al., 2021a).

Kolmogorov-Arnold Networks (KANs) (Liu et al., 2024b,c; Wang et al., 2024d) have been proposed as an alternative to MLPs, offering advantages such as enhanced interpretability, high accuracy in function regression, and resilience to catastrophic forgetting and spectral bias (Samadi et al., 2024; Vaca-Rubio et al., 2024; Wang et al., 2024d). Similar to Kolmogorov Networks (He, 2023; Köppen, 2002; Lai & Shen, 2021 (v1; Leni et al., 2013; Schmidhuber, 1997; Sprecher & Draghici, 2002) and other related architectures (Borri et al., 2024; Polar & Poluektov, 2021a), KANs are inspired by the Kolmogorov-Arnold Representation Theorem (KART), which provides a framework for decomposing multivariate functions into sums of univariate inner and outer functions (He, 2023; Köppen, 2002; Lai & Shen, 2021 (v1; Leni et al., 2013; Polar & Poluektov, 2021b; Schmidhuber, 1997; Somvanshi et al., 2024; Sprecher & Draghici, 2002). Despite their strengths, the original KANs (Liu et al., 2024c) diverge from the original KART and employ a stacked representation that relies on computationally expensive learnable B-splines as basis functions. Furthermore, their performance rapidly degrades with high parameter counts, limiting their application for SciML and raising questions about their suitability for real-world tasks (Pal and Das, 2024).

To address these limitations, several variations of KANs have been introduced (Bhattacharjee, 2024; Bozorgasl & Chen, 2024; Li, 2024; NLNR, 2024; Sidharth et al., 2024). Notable examples include FastKANs (Li, 2024) and cKANs (Sidharth et al., 2024), which use radial basis functions (RBFs) and Chebyshev polynomials as basis functions, respectively. These methods improve computational efficiency but still exhibit drawbacks. For instance, cKANs, while faster than KANs, are slower than MLPs and exhibit instabilities in single-precision arithmetic. Recursive formulations (Shukla et al., 2024; Wu et al., 2025) can restore cKAN stability, but tuning hyperparameters remains challenging (Toscano et al., 2025a), while performance degrades with an increasing number of parameters or higher polynomial orders.

KANs have been successfully extended to PIML (Liu et al., 2024c; Shukla et al., 2024; Toscano et al., 2025b; Wang et al., 2025) and operator learning (Abueidda et al., 2024; Shukla et al., 2024), with several specialized architectures and applications introduced, such as GRU-KAN (Zhang et al., 2024), separable physics-informed KANs (Jacob et al., 2024), multifidelity KANs (Howard et al., 2024b), finite basis KANs (Howard et al., 2024a), and others (Fareaa & Celebi, 2024; Ma et al., 2024; Mostajeran & Faroughi, 2024; Rigas et al., 2024). Other frameworks inspired by different versions of the Kolmogorov superposition theorem have also been proposed, such as AcNet (Guilhoto & Perdikaris, 2024). Some of these approaches have demonstrated advantages over MLPs in PIML, such as robustness to noise (Shukla et al., 2024) and reduced network size requirements (Toscano et al., 2025a). However, due to the maturity of MLPs, achieving comparable performance to a fully optimized MLP often requires extensive hyperparameter tuning and specialized training strategies (Guilhoto & Perdikaris, 2024), such as learning rate warmup, gradient clipping, and causality-enforcing methods. These challenges highlight the limitations of existing KAN-based models, which often deviate from the original theorem by employing deeply nested architectures.

From the theoretical perspective, KANs’ nested formulations (Liu et al., 2024c) and some of their variations (Guilhoto & Perdikaris, 2024) have been shown to be universal approximators. Notably, Wang et al. (2024d) compared the approximation capabilities of KANs and MLPs, demonstrating that KANs’ representation capabilities are at least as good as those of MLPs, and analyzed KANs’ resistance to spectral bias. Additionally, Zhang and Zhou (2024) presented a theoretical analysis of KANs by establishing generalization bounds, and Guilhoto and Perdikaris (2024) proved that their initialization scheme scales with the size of the network and does not suffer from vanishing derivatives. Furthermore, convergence estimates for one of KAN’s predecessors are provided in Igelnik and Parikh (2003). However, some of these studies relied on assumptions restricted to specific types of basis functions and imposed regularity conditions on the outer mappings, which are not valid for a broader class of functions.

To address these theoretical and computational challenges, we propose the Ku̇rková-Kolmogorov-Arnold Networks (KKANs) inspired by a special variant of the Kolmogorov-Arnold representation theorem by Ku̇rková (1991, 1992). KKANs consist of a new two-block architecture that adheres closely to the original theorem. KKANs use robust MLPs as inner functions and linear combinations of basis functions as outer functions. This design combines the robustness and adaptability of MLPs with the interpretability and flexibility of basis function representations (Liu et al., 2024c). We prove that KKANs are universal approximators regardless of the choice of basis function and for a general class of functions. Then, we extend their applicability to PIML and operator learning frameworks. Additionally, we demonstrate that KKANs can integrate modern enhancements originally developed for MLPs, such as weight normalization (Salimans & Kingma, 2016), Fourier feature expansions (Wang et al., 2021c), and residual connections (Wang et al., 2024a). These adaptations, previously challenging for KANs, unlock KKANs’ potential for a wide range of applications. Experimentally, we demonstrate that KKAN performance remains robust with an increasing number of parameters and is stable for a high number of basis functions. Through extensive benchmarking, we observe that KKANs outperform MLPs in function approximation and operator learning tasks, achieve comparable performance, and can outperform several state-of-the-art MLPs in PIML.

In addition to its structural advantages, we analyze the learning dynamics of KKANs, cKANs, and MLPs via the Information Bottleneck (IB) method (Tishby et al., 2000) and identify a strong correlation with the geometric complexity evolution (Dherin et al., 2022). We observe that KKANs and cKANs exhibit lower complexity than MLPs at initialization, which could be correlated with enhanced generalization capabilities. Using the IB theory, we identify three distinct learning stages: fitting, transition, and diffusion (Anagnostopoulos et al., 2024a) across all architectures and for all types of problems (i.e., function approximation, PIML and neural operators), with optimal generalization achieved during the diffusion stage when the signal-no-noise ratio (SNR) of the backpropagated gradients is high.

Finally, we propose new optimization techniques for PIML. Specifically, we extend the residual-based attention weights (RBA) introduced in Anagnostopoulos et al. (2024b) and Toscano et al. (2025a, 2024) to self-scaled RBA (ssRBA), a method that dynamically adapts during learning to maintain a high SNR. This ensures uniform convergence across the domain and enables prolonged learning. Additionally, we introduce a scaling weighting strategy that combines the ideas presented in Wang et al. (2021a) and Liu et al. (2024a) to automatically balance the loss-specific gradients, which enables achieving an optimal update direction, improving both stability and training efficiency.

In summary, the key contributions of this work are as follows:

- We propose KKAN, a novel two-block architecture that combines the robustness of MLPs with the interpretability and flexibility of basis function representations, adhering closely to the Kolmogorov-Arnold theorem.
- We prove that KKAN is a universal approximator regardless of the basis function selection and for a general class of functions.
- We extend KKAN’s applicability to PIML and operator learning.
- We analyze KKAN’s learning dynamics using information bottleneck theory, identifying a strong correlation between geometric complexity and SNR and corroborating the diffusion stage as the point of optimal generalization.
- We develop self-scaled residual-based attention weights (ssRBA) that dynamically maintain a high SNR, enabling uniform convergence and prolonged training.
- Through extensive benchmarking, we demonstrate that KKANs outperform MLPs and cKANs in function approximation and operator learning tasks and achieve comparable performance with fully optimized MLPs for PIML applications.

The remainder of this paper is organized as follows. In Section 2, we review the Kolmogorov-Arnold representation theorem and its key variants, concluding with a universal approximation theorem for two-block structures, which we use to introduce the KKAN architecture. Section 3 describes the methodology, including function approximation, PIML, operator learning frameworks, and evaluation criteria such as relative errors, geometric complexity, and learning dynamics. Additionally, we detail the proposed self-scaled residual-based attention (ssRBA) method. In Section 4, we present computational experiments, including benchmarks for function approximation, PIML, and operator learning. Section 5 analyzes learning dynamics across the proposed models and their connection to the geometric complexity. Finally, in Section 6, we summarize our findings and discuss potential future research directions.

To support reproducibility, the source code for our implementation and data are publicly available in our GitHub repository: https://github.com/jdtoscano94/KKANs_PIML.git.

## 2. Kolmogorov-Arnold representation theorem (KART)-inspired architecture

### 2.1. History and representative formulations

In a series of papers (Arnold, 1957a,b; Kolmogorov, 1956, 1957), Andrey Kolmogorov and Vladimir Arnold studied the representation of continuous functions of several variables on a bounded domain by superpositions of continuous functions of a smaller number of variables. Their astonishing discovery was summarized as the Kolmogorov-Arnold Representation Theorem (KART) in Kolmogorov (1957) (1957): *For any integer* d≥2, *there are continuous real functions* $\psi_{p,q}(x)$ *on the closed unit interval* $E^{1}=[0,1]$ *such that each continuous real function* $f\left(x_{1},\ldots ,x_{d}\right)$ *on the* d-*dimensional unit cube* $E^{d}=[0,1{]}^{d}$ *is representable as*

(1) $$f\left(x_{1},\ldots ,x_{d}\right)=\sum_{q=0}^{2d}g_{q}\left(\sum_{p=1}^{d}\psi_{p,q}\left(x_{p}\right)\right),$$

*where* $g_{q}(y)$ *are continuous real functions on R=(-∞,∞)*. Here, $\psi_{p,q}$ are known as the *inner functions* (which are universal and independent of f), while $g_{q}$ are referred to as the *outer functions* (which depend on f). This theorem and a previous result of Arnold (1957b) can be regarded as a refutation of Hilbert’s Problem 13: “*There are continuous functions of three variables, not representable as superpositions of continuous functions of two variables*” (see Girosi & Poggio, 1989).

Over the past several decades, research following this mathematically elegant representation has advanced mainly along two lines: (i) construction of refined versions to strengthen the connection to neural networks (with a two-fold focus on the reduction of the number of involved one-dimensional functions and on what smoothness conditions could be imposed on them); and (ii) theory to practical applications in deep learning and general machine learning.

In Table 1, we summarize some representative variants of the original KART. Lorentz (1962) first observed that the outer functions $g_{q}$ can be chosen to be all the same, and sketched the proof of the two-dimensional version. Subsequently, Sprecher (1963) showed that one could take the inner functions $\psi_{p,q}(x)=\lambda_{p}\psi_{q}(x)$ with suitable constants $\lambda_{q}$. This inspired the Lorentz’s version formulated in Lorentz (1966) [Chapter 11], where $g_{q}=g$ and $\psi_{p,q}(x)=\lambda_{p}\psi_{q}(x)$ with $\lambda_{p}\in (0,1)$ being arbitrary rationally independent numbers (i.e., a relation $\sum_{p=1}^{d}r_{p}\lambda_{p}=0$, with rational $r_{p}$ is possible, only if all $r_{p}=0$), and $\psi_{q}$ are strictly increasing and of α-Lipschitz class with α∈(0,1). Kahane (1975) (also see Lorentz et al., 1996, Chapter 15) provided an elegant proof of Lorentz’s version with the improvement of regularity: $\psi_{q}\in {\text{Lip}}_{1}1$ (i.e., both the Lipschitz index and constant are 1), where an additional condition for the inputs $\lambda_{p}$ is $\sum_{p=1}^{d}\lambda_{p}\leq 1$. Sprecher’s version (refined from his earlier versions in Sprecher (1965, 1996)) was stated in Sprecher (1997), where the parameters m≥2d,γ≥m+2, and

(2) $$a=\frac{1}{\gamma (\gamma -1)};\;\lambda_{1}=1,\;\lambda_{p}=\sum_{r=1}^{\infty }\gamma^{-(p-1)\left(d^{r}-1\right)/(d-1)},\;2\leq p\leq d.$$

Given these parameters, the finite domain and range of the outer and inner functions are [0, 2] and $\left[0,\frac{2(\gamma -1)}{\gamma -2}\right]$, respectively (see Montanelli & Yang, 2020). Here, the function ψ, generating all inner functions, is of $\frac{\text{ln}\;2}{\text{ln}\;\gamma }$-Lipschitz class and monotonically increasing. The modified version in Braun (2009) was resulted from a similar construction by Sprecher. It is noteworthy that (a) the introduction of an additional dilation $c_{q}$ was first realized by Sprecher (1965); and (b) the numerical construction in Sprecher (1996, 1997) could not guarantee the continuity and monotonicity of ψ. To remedy the issue (b), Köppen (2002) modified Sprecher’s recursive formula, and Braun and Griebel (2009) provided a theoretical justification for the desired continuity and monotonicity of the modified ψ. Interested readers are referred to Sprecher’s book (Sprecher, 2017) and the references therein for many more developments and insights of KART up to 2017. Recently, Schmidt-Hieber (2021) constructed a much-simplified version using the notion of space-filling curves (more precisely, the Lebesgue curve on a Cantor set 𝒞, see (Bader, 2013, Chapter 7)). Remarkably, it transfers the smoothness of f to the outer function g in the sense that if f is β-smooth (i.e., ∃β∈(0,1], such that $|f(x)-f(y)|\leq Q|x-y{|}_{\infty }^{\beta }$ for any $x,y\in E^{d}$ and some constant Q>0), then g is α-smooth with $\alpha =\frac{\beta \;\text{log}\;2}{d\;\text{log}\;3}$ on the Cantor set 𝒞⊂[0,1]. More recently, Ismayilova and Ismailov (2024) and Ismailov (2024) revisited Sprecher’s representation (with m=2d, the parameters given in (Eq. 2), and a dilation $c_{q}=(2d+1)q$), and proved that if f is continuous, discontinuous but bounded, or unbounded, then the outer function can be constructed to share the same properties (i.e., g is continuous, discontinuous but bounded, or unbounded, respectively).

Inspired by KART on a bounded domain, Doss (1977) and Demko (1977) were among the earliest to study the superposition of functions on $R^{d}$ in the form: $f(x)=\sum_{q=1}^{m}g_{q}\circ \phi_{q}(x)$, and the problem of interest was also extended to f∈C(X) (the set of real continuous functions on a topological space X). Hattori (1993), and Feng and Gartside (2011) proved the *existence of* 2d+1 *continuous functions* $\phi_{q}$ on every locally compact separable metric space with dim(X)≤d, which particularly holds for $X=R^{d}$. In summary, the number of inner functions in the KART/variants and even more general representations: $f(x)=\sum_{q=1}^{m}g_{q}\circ \phi_{q}(x)$ must be at least 2d+1 (see Sternfeld, 1985 and Lorentz et al., 1996, Chapter 17). Notably, Laczkovich (2021) showed that $\phi_{q}$ can be chosen as the KART’s inner structure: $\phi_{q}(x)=\sum_{p=1}^{d}\lambda_{p}\psi_{q}\left(x_{p}\right)$. In other words, the KART is valid on $R^{d}:$ *Let d≥2 and* $m>(2+\sqrt{2})d$ *be integers, and let $\left\{\lambda_{1},\ldots ,\lambda_{d}\right\}$ be distinct positive numbers. Then there are continuous functions $\psi_{1},\ldots ,\psi_{m}\in C(R)$ with the following property: for every bounded $f\in C\left(R^{n}\right)$*, *there is a continuous function g∈C(R) such that*

(3) $$f\left(x_{1},\ldots ,x_{d}\right)=\sum_{q=1}^{m}g\left(\sum_{p=1}^{d}\lambda_{p}\psi_{q}\left(x_{p}\right)\right).$$

Laczkovich (2021) also presented a modified version with $m>(2+\sqrt{2})(2d-1)$, *monotonically increasing inner functions $\psi_{q}$*, and constructed positive constants $\lambda_{p,q}$ in place of $\lambda_{p}$ in (Eq. 3), which shares the same monotonicity with several versions in Table 1.

As commonly recognized today, the KART/variants can be naturally translated into feed-forward neural networks with two hidden layers. The groundbreaking transition from theory to practice is attributed to Hecht-Nielsen (1987) (1987), who essentially mapped Sprecher’s construction (Sprecher, 1965) to a neural network. Hecht-Nielsen’s network is foundational (Sprecher, 2017, Chapter 5), but this short conference paper (Hecht-Nielsen, 1987) concluded with some neutral (perhaps pessimistic) views: “*The Kolmogorov’s mapping neural network existence theorem for approximations of functions by networks is, at least in theory, sound, but the direct usefulness of this result is doubtful*.” Girosi and Poggio (1989) further argued that “*Kolmogorov’s theorem: an exact representation is hopeless in representation properties of networks*” for at least two reasons:

- the inner and outer functions lack smoothness, so Kolmogorov’s network may lose generalization and stability against noise;
- Kolmogorov’s network is not the type of parameterized representation with modifiable/learnable parameters.

Indeed, one theoretical evidence for the first point is the existence of differentiable functions of d≥2 variables that cannot be expressed as a superposition of differentiable functions of fewer than d variables (see Vitushkin (1954) on the Hilbert’s Thirteenth Problem). In other words, if the KART and its variants are universal (i.e., can represent all multivariate continuous functions), we should not expect all the involved one-dimensional functions to be differentiable or more regular.

On the contrary, Ku̇rková (1991, 1992) advocated for the relevance of the KART in multilayer neural networks and asserted that

- One should sacrifice the exactness of representation by adopting an approximate version instead.

Taking advantage of the fact that any continuous function on a closed interval can be approximated arbitrarily well by shallow neural networks with sigmoidal activation function (see e.g., Cybenko, 1989), Ku̇rková, 1992 introduced an approximate representation, where the resulting KART-inspired NN has the universal approximability to $C\left(E^{d}\right)$ (see Theorem 1 below). In addition, Brattka (2007) provided some deep insights into the computability of the KART, which admits an algorithm that, in principle, allows a Turing machine to evaluate the functions up to any prescribed precision, and where the computable version derived was based upon Sprecher’s construction and Lorentz’ contraction mapping. Very recently, Freedman (2024) commented that KART may illuminate neural network learning and set its foundation. In a nutshell, the astonishing representation of the KART/variants present numerous theoretical and practical issues that deserve in-depth investigation. Indeed, the inherent mystery of this theory may at times lead to misconceptions and misinterpretations (Ismailov, 2024).

### 2.2. Two-block approximate Kolmogorov’s representation and its universality

Our network construction is influenced by Ku̇rková’s philosophy (Ku̇rková, 1991, 1992), and we aim to construct suitable *approximate Kolmogorov’s representations with the following features*:

- Adhere to the structure of KART or its variants;
- Establish universal approximability to all continuous functions;
- Allow to configure the neural networks simply by inner and outer blocks.

Our starting point is to generalize the theory in Ku̇rková (1991, 1992) and then construct the approximate representations. In general, we consider Kolmogorov’s representation:

(4) $$f\left(x_{1},\ldots ,x_{d}\right)=\sum_{q=0}^{m}g_{q}\left(\sum_{p=1}^{d}\psi_{p,q}\left(x_{p}\right)\right),$$

where $m\geq 2d+1,g_{q}\in C\left(I_{g}\right)$ and $\psi_{p,q}\in C\left(I_{\psi }\right)$. Note that the inner and outer functions and their domains can be adapted to different versions in Table 1 (where $g_{q}$ may be the same and $\psi_{p,q}$ may be $\lambda_{p}\psi_{q}$ with given $\lambda_{p}$, etc.). With a little abuse of notation, we use $I_{g},I_{\psi }$ (resp. $M_{g},M_{\psi }$) to denote intervals (resp. discretizations parameters) corresponding to the outer functions $g_{q}$ and inner functions $\psi_{p,q}$, respectively.

**Definition 1 (Set of approximators).** Define the subset of $C\left(E^{d}\right)$ generated by the KART/variants:

(5) $$K_{M}^{m,d}=\{F\left(x\right)=\sum_{q=0}^{m}G_{q}\left(\sum_{p=1}^{d}\Psi_{p,q}\left(x_{p}\right)\right):\forall G_{q}\in {𝒜}_{M_{g}}\left(I_{g}\right),\left.\forall \Psi_{p,q}\in {𝒜}_{M_{\psi }}\left(I_{\psi }\right)\right\},$$

where the ansatz spaces ${𝒜}_{M_{g}}\left(I_{g}\right)$ and ${𝒜}_{M_{\psi }}\left(I_{\psi }\right)$ are chosen to be dense subsets of the spaces of continuous functions $C\left(I_{g}\right)$ and $C\left(I_{\psi }\right)$ containing the true outer functions $g_{q}$ and inner functions $\psi_{p,q}$, respectively. It has the cardinality:

(6) $$P≔\#K_{M}^{m,d}=\left(m+1\right)M_{g}+d\left(m+1\right)M_{\psi }=\left(m+1\right)\left(M_{g}+dM_{\psi }\right),$$

where we assume the cardinality of ${𝒜}_{M_{z}}\left(I_{z}\right)$ is $M_{z}$ with z=g,ψ.

As a generalization of the theory in Ku̇rková (1992), we can show the universal approximability of the approximate representations (see Appendix A for the proof).

**Theorem 1 (Universal approximation theorem).** *Let* d≥2. *Assume that* ${𝒜}_{M_{z}}\left(I_{z}\right)$ *are dense in* $C\left(I_{z}\right)$ *for* z=g,ψ. *Then the subset* $K_{M}^{m,d}$ defined in (Eq. 5) *is dense in* $C\left(E^{d}\right)$ *with* $E^{d}=[0,1{]}^{d}$ *in the sense that for any* $f\in C\left(E^{d}\right)$ *and any* ε>0, *there exists* $F\in K_{M}^{m,d}$ (*i.e*., $\exists M_{g},M_{\psi }\in N$ *depending on* ε) *such that*

(7) $$\|f-F{\|}_{\infty }=\underset{x\in E^{d}}{\text{sup}}|f(x)-F(x)|<\varepsilon ,$$

*where* $x=\left(x_{1},\ldots ,x_{d}\right)$.

In Kúrková (1992), ${𝒜}_{M_{z}}\left(I_{z}\right)$ were both chosen as the set of staircase-like functions of a type σ (sigmoidal function), that is,

(8) $${𝒜}_{M_{z}}\left(I_{z}\right)≔\left\{\sum_{i=1}^{M_{z}}a_{i}\sigma \left(b_{i}x+c_{i}\right):a_{i},b_{i},c_{i}\in R,x\in I_{z}\right\},\;z=g,\psi ,$$

which are universal approximators of $C\left(I_{z}\right)$ (see e.g., Cybenko, 1989; Petersen & Zech, 2024). In theory, one can choose ${𝒜}_{M_{z}}\left(I_{z}\right)$ to be any ansatz classes consisting of orthogonal polynomials/functions (used in spectral methods), splines, wavelets and radial basis functions, among others. The interested readers are referred to Lorentz et al. (1996) and various other resources for their universal approximability and convergence estimates. On the other hand, one can also parameterize the one-dimensional inner/outer functions by neural networks such as MLPs.

It is noteworthy that Igelnik and Parikh (2003) proposed the Kolmogorov spline network (KSN) inspired by the Lorentz’s version:

(9) $$F_{M}^{s}\left(x\right)=\sum_{q=0}^{M}s\left(\sum_{p=1}^{d}\lambda_{p}s\left(x_{p},\gamma_{p,q}\right),\gamma_{q}\right),$$

where $s\left(\cdot ,\gamma_{q}\right)$ and $s\left(\cdot ,\gamma_{p,q}\right)$ are cubic splines with adjustable parameters $\gamma_{q},\gamma_{p,q}$, respectively, and the constants $\lambda_{p}$ are given in the Lorentz’s representation. They claimed that the KSN has a convergence rate for any $f\in C^{1}\left(E^{d}\right)$ (continuously differentiable functions defined on $[0,1{]}^{d}$ with bounded gradient):

(10) $${\left\|f-F_{M}^{s}\right\|}_{\infty }=𝒪\left(M^{-1}\right),$$

where the number of net parameters $P=𝒪\left(M^{3/2}\right)$. The estimate (Eq. 10) essentially relies on an intermediate approximate version of Kolmgorov-Arnold superpositions with $C^{4}$-smooth inner and outer functions whose representations were derived from the constructive proof in Kahane (1975) based on the Baire Category Theorem. However, the inner functions still suffer from the “wildness” and “pathology” with zero derivatives almost everywhere and wildly growing derivative values of the cubic splines in small gaps (see Demb & Sprecher, 2021). Therefore, their claim for the uniform boundedness of all derivatives up to forth-order appears questionable. In other words, the network size $P=𝒪\left(M^{3/2}\right)$ must have an order much bigger than 3/2.

We remark that Lai and Shen (2021 (v1) studied the approximate Lorentz’s representation via ReLU NN parameterisation, where a first-order convergence was obtained under the assumption that the outer function is Lipschitz continuous (see Kahane, 1975). Lai and Shen (2024) attempted to estimate higher order convergence for the spline networks, but the differentiability and stronger regularity must be imposed on the outer functions. Although there are some functions in such compositions satisfying the regularity assumption (e.g., $x_{1}x_{2}\cdots x_{d}=\text{exp}\left(\text{ln}\;x_{1}+\text{ln}\;x_{2}+\cdots +\text{ln}\;x_{d}\right)$ for all $x_{i}>0$), one may not expect such a regularity for general multi-dimensional functions. After all, the highest regularity of the outer functions by construction is Lipschitz (see Table 1). Similar assumptions were made in the KANs by Liu et al. (2024c) and some other variants for the stacked KART with an aim to approximate composite functions with more layers (e.g., one representative function in Liu et al., 2024c: $\left.f\left(x_{1},x_{2},x_{3},x_{4}\right)=\text{exp}\left(\text{sin}\left(x_{1}^{2}+x_{2}^{2}\right)+\text{sin}\left(x_{3}^{2}+x_{4}^{2}\right)\right)\right)$. Nevertheless, the theory of the stacked KANs in Liu et al. (2024c)[Theorem 2.1] holds for a specific class of functions defined by compositions.

We reiterate that the inner functions in KART are independent of the functions to be represented, while the outer functions are data-dependent. This suggests the use of different ansatz or parameterisation for the inner and outer functions, leading to the two-block architecture below.

### 2.3. KKAN architecture

**Fig. 1.**
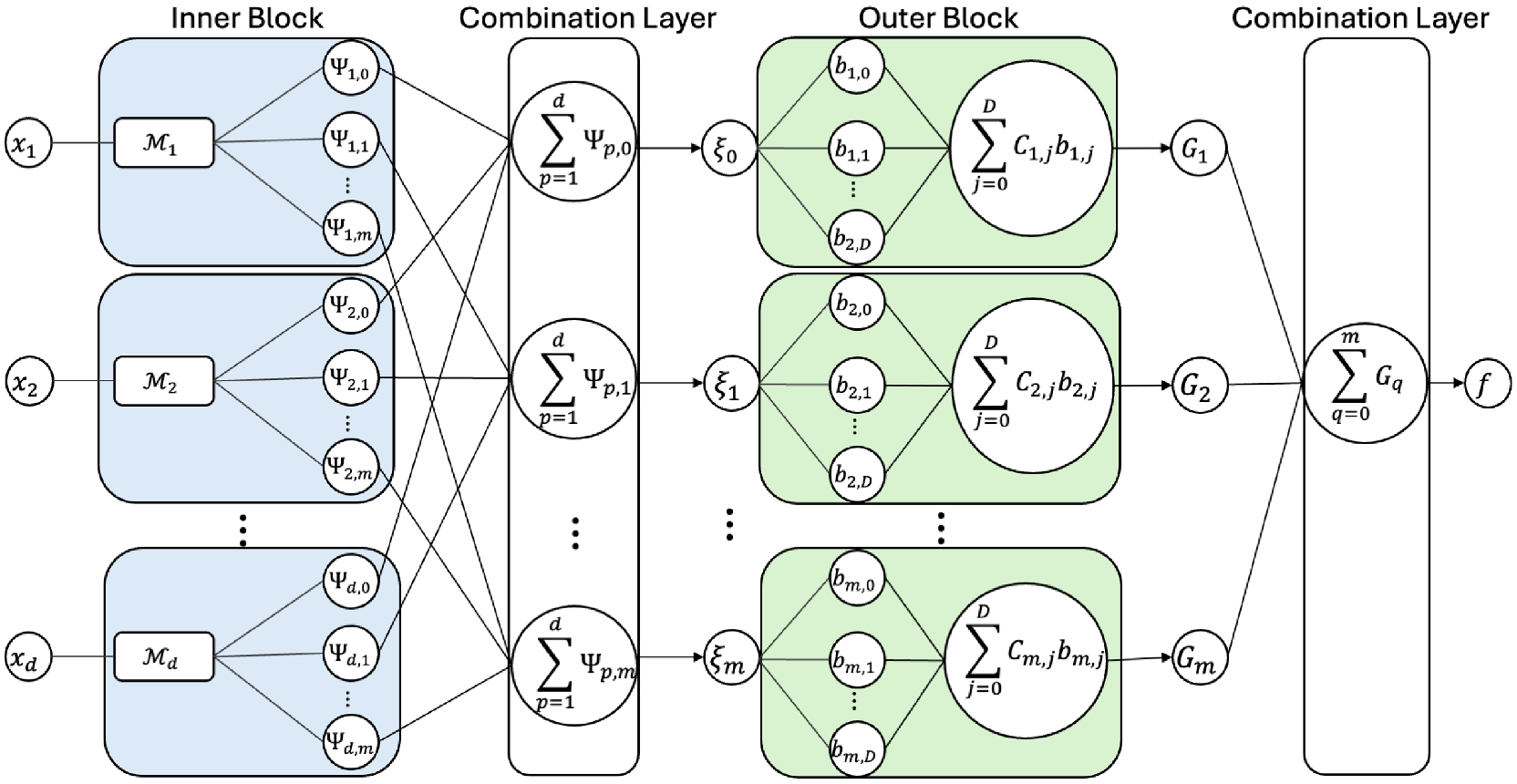
KKAN-Inspired architecture. The inner block computes the inner functions by expanding each input dimension into an m-dimensional space. The first combination layer sums the inner functions across the input dimensions, i.e., $\xi_{q}=\sum_{p=1}^{d}\Psi_{p,q}\left(x_{p}\right)$, to obtain an m-dimensional vector $\xi =\left[\xi_{0},\ldots ,\xi_{m}\right]$. The outer block computes the outer functions by transforming each $\xi_{q}$, and the final combination layer sums all the outer functions $G_{q}$, enabling the approximation of the target function, closely mimicking the KART.

The proposed architecture aims to closely mimic the KART variations, described as:

(11) $$f\left(x_{1},\ldots ,x_{d}\right)=\sum_{q=0}^{m}G_{q}\left(\sum_{p=1}^{d}\Psi_{p,q}\left(x_{p}\right)\right),$$

(12) $$f\left(x_{1},\ldots ,x_{d}\right)=\sum_{q=0}^{m}G_{q}\left(\xi_{q}\right),$$

where $\Psi_{p,q}\left(x_{p}\right)$ and $G_{q}\left(\xi_{q}\right)$ are referred to as the *inner* and *outer* functions, respectively. Inspired by this representation, we divide our architecture into an **inner block, combination layers**, and an **outer block** (see Fig. 1).

In this study, we propose defining trainable inner and outer blocks, enabling the approximation of multivariate functions with enhanced flexibility and expressiveness. The inner block computes the inner functions by expanding each input dimension into an m-dimensional space. The first combination layer sums the inner functions across the input dimensions, i.e., $\xi_{q}=\sum_{p=1}^{d}\Psi_{p,q}\left(x_{p}\right)$, to obtain an m-dimensional vector $\xi =\left[\xi_{0},\ldots ,\xi_{m}\right]$. The outer block then computes the outer functions by transforming each $\xi_{q}$. Finally, the last combination layer sums all the outer functions $G_{q}$, enabling the approximation of the target function closely mimicking (11).

Notably, the proposed architecture bears a resemblance to Tensor Neural Networks (Wang et al., 2022b) and Separable PINNs (Cho et al., 2024), with the key difference being that our architecture combines dimensions via summation instead of using the tensor product.

#### 2.3.1. Inner block

The role of the inner block is to obtain the inner functions by expanding each input dimension into an m-dimensional space. Towards this end, we propose using Multi-Layer Perceptrons (MLPs) due to their flexibility, strong approximation capabilities, and continuous advancements in the deep learning community.

Additionally, we enhance the baseline MLP by drawing inspiration from the “adaptive basis viewpoint” introduced in Cyr et al. (2020). Under this perspective, an MLP is considered a mesh-free technique that constructs an adaptive basis, where the output is obtained by a linear combination of basis functions in the last linear layer. This viewpoint intuitively justifies the effectiveness of using suitable input transformations (Cai & Xu, 2019; Wang et al., 2024a, 2020) to improve approximation capabilities in PIML.

**Fig. 2.**
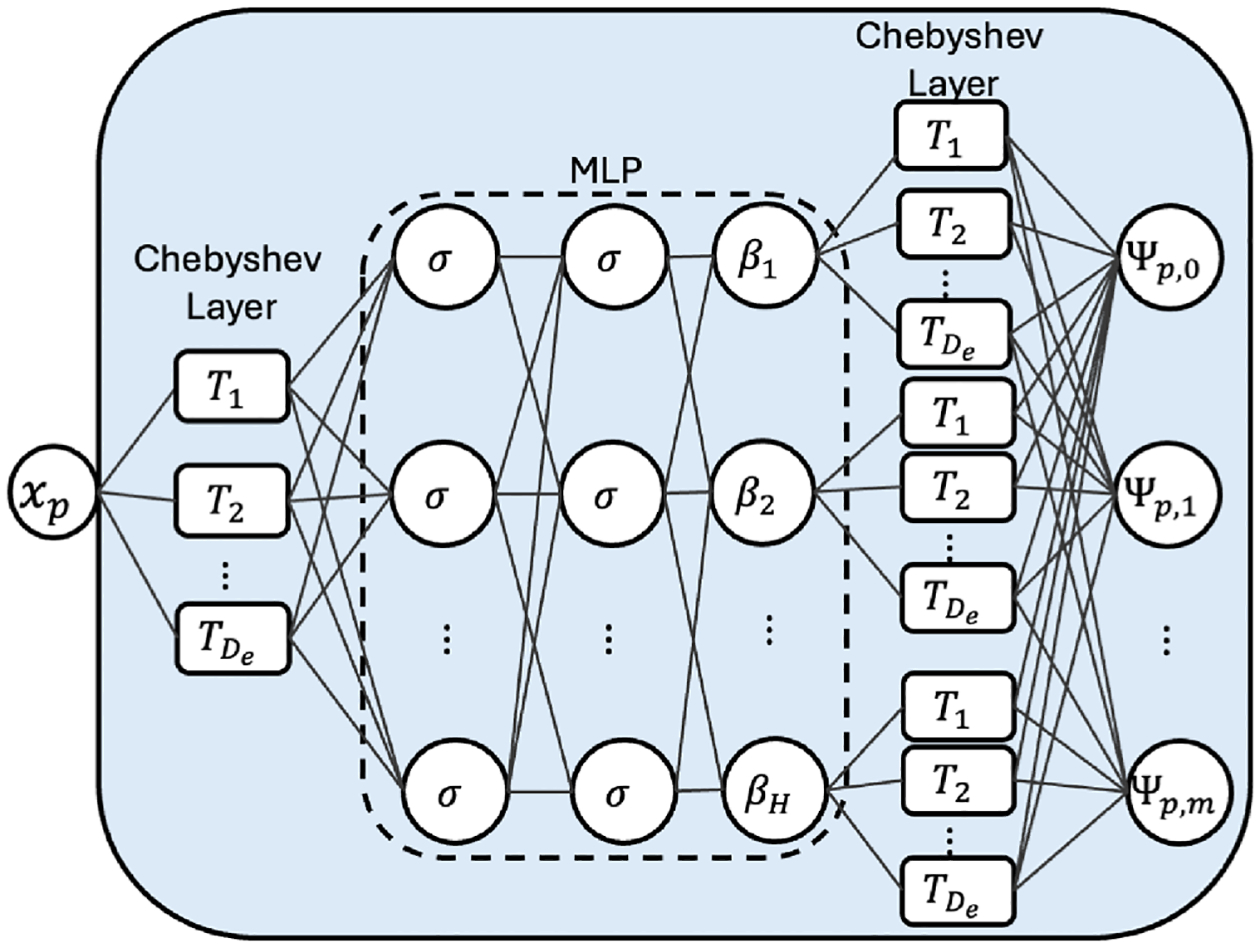
Enhanced-basis MLP (ebMLP). Each inner block expands its respective input dimension into an m-dimensional space using an enhanced Multi-Layer Perceptron (MLP). The ebMLP incorporates two trainable Chebyshev layers that perform orthogonal expansions of the inputs ($x_{p}$) and outputs ($\beta_{i}$), improving the quality of the basis functions and enhancing the network’s representation capabilities.

To further refine the basis construction, we introduce two trainable Chebyshev layers that enable us to obtain orthogonal expansions of the inputs ($x_{p}$) and outputs ($\beta_{i}$) of the baseline MLP (Toscano et al., 2025b). This architecture, which we denote as the enhanced-basis MLP (ebMLP), is used to construct each component of our inner block (see Fig. 2). By incorporating orthogonal basis functions through the Chebyshev layers, we increase the network’s representation capabilities and improve its ability to approximate complex functions more effectively.

#### 2.3.2. Outer block

For the outer block, we follow the approach in Liu et al. (2024c) and model each outer function as a linear combination of basis functions (see Fig. 1). Specifically, each outer function is defined as:

$$g_{q}\left(\xi_{q}\right)=\sum_{j=0}^{D}C_{q,j}b_{q,j}\left(\xi_{q}\right),$$

where $b_{q,j}\left(\xi_{q}\right)$ are suitable basis functions, $C_{q,j}$ are the corresponding trainable coefficients, and D is the expansion degree. By selecting appropriate basis functions and making the coefficients trainable, the outer blocks can flexibly model the nonlinear transformations required to accurately approximate the target function.

By combining the strengths of the inner and outer blocks, the architecture effectively captures the underlying structure described by the KART (i.e., Eq. (11)). This leads to enhanced flexibility and expressiveness in approximating multivariate functions. A detailed description of the proposed implementation and the basis functions considered in this study is provided in Appendix B.3.

## 3. Methodology

This study compares three representation models—KKANs, cKAN (i.e., KANs Liu et al., 2024c with Chebyshev basis functions), and MLPs—for function approximation, physics-informed machine learning (PIML), and data-driven Neural Operators (NOs). The trainable parameters for all models, denoted as θ, are optimized by minimizing a case-specific loss. Detailed formulations for KKANs, MLPs, and cKANs are provided in Section 2.3, Appendices B.1, B.2, and B.3.

### 3.1. Function approximation

The goal of this test is to evaluate the ability of representation models to fit a specific function. Here, the function $\overset{ˆ}{u}(x)$ is approximated by a representation model u(θ,x), where θ are the trainable parameters:

(13) $$\overset{ˆ}{u}\left(x\right)\approx u\left(\theta ,x\right),\;x\in \Omega .$$

The training process minimizes the data residuals:

(14) $$r_{D}\left(x,\theta \right)=u\left(x,\theta \right)-\overset{ˆ}{u}\left(x\right),\;x\in \Omega_{D},$$

where $\Omega_{D}\subset \Omega$ contains all available observations. The corresponding loss function is:

(15) $$ℒ={ℒ}_{D}=\sum_{i=1}^{N}\lambda_{D,i}r_{D}^{2}\left(x_{i},\theta \right),\;x_{i}\in \Omega_{D},$$

where $\lambda_{D,i}$ are local weights that balance the contribution of each training point (Anagnostopoulos et al., 2024a).

### 3.2. Physics-informed machine learning and operator learning

SciML is agnostic to specific governing laws, leveraging machine-learning-based models to approximate solutions (Toscano et al., 2025b). We consider the general nonlinear ODE/PDE system:

(16a) $${ℱ}_{\tau }\left[\overset{ˆ}{u}\right]\left(x\right)=f\left(x\right),\;x\in \Omega ,$$

(16b) $${ℬ}_{\tau }[\overset{ˆ}{u}](x)=b(x),\;x\in \Omega_{B,}$$

where x represents the spatial-temporal coordinate, $\overset{ˆ}{u}$ is the solution, τ are the parameters, and ℱ and ℬ are nonlinear differential and boundary operators, respectively.

In this study, we focus on two SciML approaches: Physics-Informed Machine Learning (PIML) and Neural Operators (NOs). The main difference between PIML and NO is that the former targets solving one specific ODE/PDE, in which the training of the representation model gives an approximated solution that maps point to point, while the latter aims to solve a family of ODEs/PDEs, in which the representation model maps functions to functions (Shukla et al., 2024).

#### 3.2.1. Physics-informed machine learning (PIML)

PIML approximates the solution $\overset{ˆ}{u}(x)$ of a PDE/ODE using a representation model u(θ,x), ensuring that it satisfies the governing equations and any available data coming from boundary conditions, initial condition or sparse observations inside the domain. The equation and boundary residuals are described as follows:

(17) $$r_{E}\left(x,\theta \right)={ℱ}_{\tau }\left[u\right]\left(x,\theta \right)-f\left(x\right),\;x\in \Omega ,$$

(18) $$r_{B}\left(x,\theta \right)=B_{\tau }\left[u\right]\left(x,\theta \right)-b\left(x\right),\;x\in \Omega_{B}.$$

The general total loss function combines residuals for governing equations $\left({ℒ}_{E}\right)$, boundary conditions $\left({ℒ}_{B}\right)$, and data $\left({ℒ}_{D}\right)$

(19) $$ℒ=m_{E}{ℒ}_{E}+m_{B}{ℒ}_{B}+m_{D}{ℒ}_{D},$$

where each term is computed as:

(20) $${ℒ}_{\alpha }\left(\theta \right)=\sum_{i=1}^{N_{\alpha }}\lambda_{\alpha ,i}f\left(r_{\alpha }\left(x_{i},\theta \right)\right),\;x_{i}\in \Omega_{\alpha }.$$

Here, α={E,B,D} indexes the loss groups for data, boundary, and equation respectively, $m_{\alpha }$ are global weights that balance the contribution of each term, and $\lambda_{\alpha ,i}$ are local weights that balance the contribution for each training point. For inverse problems, Eq. (19) incorporates the data residuals $r_{D}$, while for forward problems, only the boundary ($r_{B}$) and equation ($r_{E}$) residuals are considered (Toscano et al., 2025b).

#### 3.2.2. Neural operators (NOs)

Neural Operators (NOs) approximate solution operators $G_{\theta }$ that map input functions, such as a source term f, to the corresponding solution u (Li et al., 2020; Lu et al., 2021a). In this study, we focus on variations of DeepONet (Lu et al., 2019) and QR-DeepONet (Lee & Shin, 2024). A detailed description of these formulations is provided in Appendix D. The loss function for the DeepONet models can be described as:

(21) $$ℒ={ℒ}_{D}=\frac{1}{N}\sum_{i=1}^{N}\frac{1}{N_{u}^{i}}\sum_{j=1}^{N_{u}^{i}}{\left\|G_{\theta }\left(v_{i}\right)\left(x_{i}^{j}\right)-u_{i}^{j}\right\|}^{2},$$

where ${\left\{v_{i},{\left\{x_{i}^{j},u_{i}^{j}\right\}}_{j=1}^{N_{u}^{i}}\right\}}_{i=1}^{N}$ are training data. Here, N is the number of data pairs, $v_{i}$ represents the input functions, and $u_{i}^{j}$ denotes observations of the output function at points $x_{i}^{j}$.

### 3.3. Training

In this study, we learn the model parameters for all our examples by iteratively minimizing their respective loss functions (i.e., Eqs. (15), (19) and (21)) using gradient-based optimizers:

(22) $$\theta^{k+1}=\theta^{k}+\alpha^{k}p^{k},$$

(23) $$p^{k}=-H_{k}\nabla_{\theta }ℒ\left(\theta^{k}\right),$$

where $\alpha^{k}$ is the step size and $H_{k}$ defines the update direction. Common optimizers include ADAM (Kingma & Ba, 2014), when $H_{k}=I$, and L-BFGS (Liu & Nocedal, 1989), with the latter achieving superlinear convergence by approximating the Hessian i.e., $H_{k}\approx {\left(\nabla_{\theta }^{2}ℒ\right)}^{-1}$ (Urbán et al., 2024).

### 3.4. Learning dynamics via the information bottleneck theory

The IB theory provides an information-theoretic framework for analyzing the training and performance of neural networks. It explains how networks form a compressed representation of layer activations, 𝒯, with respect to an input variable x∈𝒳, retaining only relevant information about the output variable y∈𝒴 (Tishby et al., 2000; Tishby & Zaslavsky, 2015).

The key concept in IB theory is the mutual information I(x,y), which quantifies how much information about y is preserved in the representation x. Optimal models balance this tradeoff by discarding irrelevant information, creating an “information bottleneck.” IB identifies two primary learning stages: fitting and diffusion (Goldfeld & Polyanskiy, 2020; Shwartz-Ziv, 2022; Shwartz-Ziv & Tishby, 2017), with a third stage, transition, proposed in Anagnostopoulos et al. (2024a) for PIML. These three stages have been observed across various representation models, including PINNs and physics-informed KANs (Shukla et al., 2024).

#### 3.4.0.1. Signal-to-Noise Ratio (SNR).

The signal-to-noise ratio (SNR) is a critical metric for understanding training dynamics. It is defined as:

(24) $$\text{SNR}=\frac{\|\mu {\|}_{2}}{\|\sigma {\|}_{2}}=\frac{{\left\|E\left[\nabla_{\theta }{ℒ}_{ℬ}\right]\right\|}_{2}}{{\left\|\text{std}\left[\nabla_{\theta }{ℒ}_{ℬ}\right]\right\|}_{2}},$$

where θ represents the network parameters, μ is the batch-wise mean of the gradients, and σ is the batch-wise standard deviation of the gradients of the batch-wise loss ${ℒ}_{ℬ}$. High SNR indicates that the gradients are signal-dominant, while low SNR corresponds to noise-dominant gradients (Goldfeld & Polyanskiy, 2020; Shukla et al., 2024). This IB-based framework not only provides a lens to analyze convergence but also offers insights into why some models generalize better than others. Models that successfully transition through all three stages, particularly those entering diffusion early, tend to exhibit superior performance (Anagnostopoulos et al., 2024a; Shukla et al., 2024). Conversely, models trapped in the transition stage generally do not converge (Toscano et al., 2025b).

### 3.5. Self-scaled residual-based attention (ssRBA)

One challenge in training neural networks is that point-wise residuals can be neglected in cumulative loss computations (McClenny & Braga-Neto, 2023). To address this issue, various methods have been developed to balance the point-wise contributions using local $\lambda_{i}$, which enhance the performance in PIML (Anagnostopoulos et al., 2024b; Basir, 2022; Basir & Senocak, 2022, 2023; Chen et al., 2024; Hao et al., 2024; McClenny & Braga-Neto, 2023; Ramirez et al., 2024; Shukla et al., 2024; Son et al., 2023; Song et al., 2024; Wu et al., 2025). In particular, Residual-Based Attention (RBA) (Anagnostopoulos et al., 2024b) experimentally have been shown to be effective in a wide range of applications and extensions (Anagnostopoulos et al., 2024a; Chen et al., 2024; Ramirez et al., 2025; Ramireza et al., 2024; Rigas et al., 2024; Shukla et al., 2024; Toscano et al., 2025a, 2024; Wang et al., 2024b,c) due to its simplicity, efficiency and accuracy. RBA uses the exponentially weighted moving average of residuals to adaptively prioritize high-error regions during training, significantly improving the model performance with minimal computational overhead.

The Residual-Based Attention (RBA) method adaptively prioritizes training points located in regions of high error. Given that point-wise residuals, $r_{\alpha ,i}$, can exhibit high-frequency fluctuations during the optimization process, RBA employs an exponentially weighted moving average to update the local multipliers, $\lambda_{\alpha ,i}$, smoothly. This iterative update is defined as:

(25) $$\lambda_{\alpha ,i}^{\left(k+1\right)}\leftarrow \gamma \lambda_{\alpha ,i}^{\left(k\right)}+\eta \frac{\left|r_{\alpha ,i}^{\left(k\right)}\right|}{{\left\|r_{\alpha }^{\left(k\right)}\right\|}_{\infty }},\;i\in \left\{0,1,\ldots ,N\right\},$$

where η is a learning rate and γ∈[0,1) is a memory term that dictates the influence of past residuals. The interplay between these parameters establishes an effective upper bound for the multipliers, $\lambda_{\text{max}}=\eta /(1-\gamma )$. Consequently, adjusting the memory term γ provides a direct mechanism for controlling the magnitude of the attention weights. This process focuses the optimization on regions with persistently high error, thereby enhancing convergence efficiency.

To handle large datasets requiring batch-wise training, Toscano et al. (2025a) proposed Residual-Based Attention with Resampling (RBA-R). Here, RBA weights define a probability density function (PDF) for resampling critical points:

(26) $$p_{\alpha }^{\left(k\right)}\left(x\right)=\frac{{\left(\lambda_{\alpha }^{\left(k\right)}\right)}^{\nu }}{E\left[{\left(\lambda_{\alpha }^{\left(k\right)}\right)}^{\nu }\right]}+c,$$

where $\lambda_{\alpha }^{\left(k\right)}={\left\{\lambda_{\alpha ,i}^{k}\right\}}_{i=1}^{N_{\alpha }}$ represents RBA weights raised to the power ν, which controls the standard deviation of the PDF, and c>0 which ensures all points are eventually sampled. Unlike prior methods (Lu et al., 2021b; Wu et al., 2023), this PDF, based on cumulative residuals, offers greater stability and computational efficiency, enabling fast sampling with negligible cost.

In this study, we propose extending the RBA method, drawing inspiration from our insights on information bottleneck theory and the signal-to-noise ratio (SNR). As discussed in Section 3.4, optimal convergence occurs during the diffusion stage when the SNR is high, indicating an “agreement” or “equilibrium” in the gradient flow. However, prior studies (Anagnostopoulos et al., 2024a; Shukla et al., 2024) demonstrate that the diffusion stage may saturate, leading to a decline in the SNR and a plateau in the generalization error. This saturation may arise due to increasing stochasticity in the later diffusion stages and diminishing gradients as the training loss decreases, resulting in machine precision limitations.

To address the challenges of training in highly stochastic regimes, such as the diffusion stage, we propose a scheduling strategy analogous to an annealing process. The core of this strategy is not to tune the memory term γ directly, but rather to sequentially increase its effective upper bound, $\lambda_{\text{max}}$. As detailed in Algorithm 1, this increase occurs in a stepwise manner at fixed intervals of $N_{\text{stage}}$ iterations, with the corresponding γ derived at each stage via the relationship $\gamma =1-\eta /\lambda_{\text{max}}$. This dynamic scheduling creates a transition from an initial “high-temperature” state (low $\lambda_{\text{max}}$ and γ) where the attention weights are highly responsive for exploration, to a final “low-temperature” state (high $\lambda_{\text{max}}$ and γ). In this later state, the weights have a longer memory, which is crucial for averaging out noise and stabilizing the training. The ultimate impact of this increased stability is the ability to maintain a high Signal-to-Noise Ratio (SNR), enabling prolonged and effective convergence.

However, for multi-objective loss functions such as PIML (i.e., Eq. 19), modifying $\lambda_{\text{max}}$ could introduce imbalances between individual loss terms (e.g., ${ℒ}_{B},{ℒ}_{E}$). To address this, prior studies have proposed adaptive strategies, such as modifying global weights (Basir, 2022; Boster et al., 2023; Cai et al., 2021; Chen et al., 2024; Jin et al., 2021; Liu & Wang, 2021; Wang et al., 2022a, 2021a; Xiang et al., 2022) or refining update directions using cosine similarities and related methods (Liu et al., 2024a; Yao et al., 2023; Zhou et al., 2023). Building on these approaches, we propose a combined method that leverages global weights to scale loss-specific gradients based on their magnitudes, resulting in an improved update direction.

Notice that for first-order optimizers such as ADAM, the update direction (i.e., Eq. (D.1)) for PIML (i.e., Eq. (19)) is given by:

(27) $$p^{k}=-m_{E}\nabla_{\theta }{ℒ}_{E}\left(\theta^{k}\right)-m_{B}\nabla_{\theta }{ℒ}_{B}\left(\theta^{k}\right)-m_{D}\nabla_{\theta }{ℒ}_{D}\left(\theta^{k}\right),$$

where $\nabla_{\theta }{ℒ}_{E},\nabla_{\theta }{ℒ}_{B}$, and $\nabla_{\theta }{ℒ}_{D}$ are the loss gradients which can be represented as high-dimensional vectors defining directions to minimize their respective loss terms. Notice that if the gradient magnitudes are imbalanced, one direction will dominate, which may lead to poor convergence (See Fig. E.16(a)). To address this challenge, we propose modifying the magnitude of the individual directions by scaling their respective global weights. In particular, we fix $m_{E}$ and update the remaining global weights using the rule:

(28) $$m_{B}^{k}=\alpha m_{B}^{k-1}+\left(1-\alpha \right)\frac{\left\|\nabla_{\theta }{ℒ}_{E}\right\|}{\left\|\nabla_{\theta }{ℒ}_{B}\right\|},$$

(29) $$m_{D}^{k}=\alpha m_{D}^{k-1}+\left(1-\alpha \right)\frac{\left\|\nabla_{\theta }{ℒ}_{E}\right\|}{\left\|\nabla_{\theta }{ℒ}_{D}\right\|},$$

where α∈[0,1] is a stabilization parameter (Wang et al., 2021a). This formulation computes the iteration-wise average ratio between gradients, enabling normalized scaling, which, on average, allows us to define a balanced update direction ${\overset{ˆ}{p}}^{k}$ (See Fig. E.16(b)):

(30) $${\overset{ˆ}{p}}^{k}\approx -m_{E}\left\|\nabla_{\theta }{ℒ}_{E}\right\|\left[\nabla_{\theta }{ℒ}_{E}\left(\theta^{k}\right)-\frac{\nabla_{\theta }{ℒ}_{B}\left(\theta^{k}\right)}{\left\|\nabla_{\theta }{ℒ}_{B}\right\|}-\frac{\nabla_{\theta }{ℒ}_{D}\left(\theta^{k}\right)}{\left\|\nabla_{\theta }{ℒ}_{D}\right\|}\right].$$

Under this approach, all loss components have balanced magnitudes, allowing each optimization step to minimize all terms effectively.

A generalized training procedure for optimizing representation models using gradient descent and self-scaled residual-based attention is summarized in Algorithm 1. For our KKAN architecture, this procedure utilizes two separate optimizers for the inner and outer blocks, with specific hyperparameters detailed in Appendix F. Similar dual-optimizer strategies have been shown to be effective for more conventional Kolmogorov-Arnold models (Poluektov & Polar, 2023).

### 3.6. Evaluation metrics

In the current study, we analyze our models (i.e., MLP, cKAN, and KKAN) under three criteria, namely (1) the relative $L^{2}$ error on the ground truth data, (2) their geometric complexity, which has been linked to generalization capabilities, and (3) the learning dynamics using the IB theory.

#### 3.6.1. Relative $L^{2}$ error

The relative $L^{2}$ error is used to benchmark the accuracy of the final predictions from different representation models. It is defined as:

(31) $$\text{Relative}\;L^{2}=\frac{\|\overset{ˆ}{u}(x)-u(x,\theta ){\|}_{2}}{\|\overset{ˆ}{u}(x){\|}_{2}}=\frac{\sqrt{\sum_{i=1}^{N}{\left(\overset{ˆ}{u}\left(x_{i}\right)-u\left(x_{i},\theta \right)\right)}^{2}}}{\sqrt{\sum_{i=1}^{N}\overset{ˆ}{u}{\left(x_{i}\right)}^{2}}}$$

where $\overset{ˆ}{u}$ denotes the ground truth, obtained either analytically (e.g., for function approximation tasks) or from high-accuracy numerical solvers (e.g., for SciML problems), and u denotes the predicted solution obtained by the representation model. This formulation is commonly used (Chen et al., 2024; McClenny & Braga-Neto, 2023; Raissi et al., 2020; Toscano et al., 2025a) to measure the deviation of the model’s predictions from the expected solution, providing a clear measure of model performance.

#### 3.6.2. Geometric complexity

Simpler models are generally preferred over more complex ones, and controlling model complexity has been a long-standing goal in machine learning through techniques like regularization, hyperparameter tuning, and architecture design. In this study, we evaluate model complexity using the notion of geometric complexity, as defined in Dherin et al. (2022). Geometric complexity measures the variability of a model’s function using the discrete Dirichlet energy:

(32) $$\left\langle f_{\theta },D\right\rangle =\frac{1}{\left|D\right|}\sum_{x\in D}{\left\|\nabla_{x}f_{\theta }(x)\right\|}_{F}^{2},$$

where $f_{\theta }$ is the model’s output, $D={\left\{x_{i}\right\}}_{i=1}^{N}$ is the training dataset, and ${\left\|\nabla_{x}f_{\theta }(x)\right\|}_{F}$ is the Frobenius norm of the Jacobian of the model’s output with respect to its input.

This metric has been connected to various standard training heuristics, such as parameter normalization, spectral norm constraints, and noise regularization. Furthermore, it has been used to study initialization strategies and phenomena like double descent (Dherin et al., 2022). By analyzing the geometric complexity, we aim to understand its relationship with the generalization capabilities of different representation models.

## 4. Results

### 4.1. Function approximation

These models were trained using the loss function described in Eq. 15. To ensure a fair comparison, we aimed to match the total number of parameters across all models. However, it was observed that cKANs could not handle a significantly higher number of parameters without degrading performance, thereby limiting their scalability. Consequently, smaller networks were used for all models.

For all cases, the model’s performance was evaluated on a dense 256 × 256 uniform grid for error reporting and visualization purposes. The training process itself, however, was performed using a distinct set of 10,000 points sampled via Latin hypercube sampling to ensure uniform domain coverage. Additional implementation details and hyperparameters are provided in Appendix F.

#### 4.1.1. Discontinuous

To evaluate the robustness of the analyzed models, we tested their performance on a highly discontinuous function inspired by Shukla et al. (2024). Originally introduced as a challenging 1D example, we extended it to 2D using a tensor product formulation:

$$f\left(x_{1},x_{2}\right)=\prod_{i=1}^{2}h\left(x_{i}\right),$$

where:

$$h\left(x_{i}\right)=\left\{\begin{array}{ll} 5+\sum_{k=1}^{4}\text{sin}\left(kx_{i}\right), & x_{i}<0,\\ \text{cos}\left(10x_{i}\right), & x_{i}\geq 0. \end{array}\right.$$

**Fig. 3.**
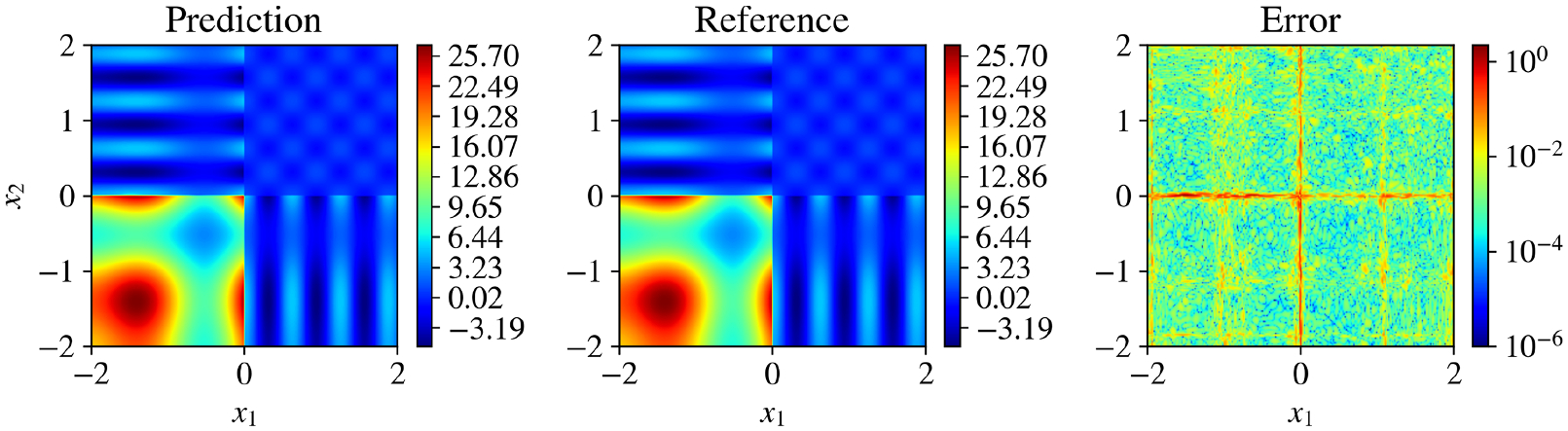
Performance of KKANs for discontinuous function approximation. Columns show predictions, ground truth references, and absolute errors, respectively. This function is particularly challenging to learn due to two discontinuities at $x_{1}=0.0$ and $x_{2}=0.0$, along with smooth regions containing relatively high frequencies. Additionally, the function exhibits a wide range of magnitudes, with outputs spanning from −5 to 25. The KKAN model achieves a relative $L^{2}$ error of 5.86 × 10^−3^.

This problem introduces abrupt changes in the function (two discontinuities along $x_{1}=0$ and $x_{2}=0$), making it particularly challenging for models trained using gradient-based methods. For the KKAN model, we used the sin-series basis function introduced in Guilhoto and Perdikaris (2024). The relative $L^{2}$ error convergence and geometric complexity for other basis functions are shown in Fig. G.17, highlighting the robustness of our approach. While the sin-series basis performs best, the model converges effectively with all the tested cases Fig. 3.

**Fig. 4.**
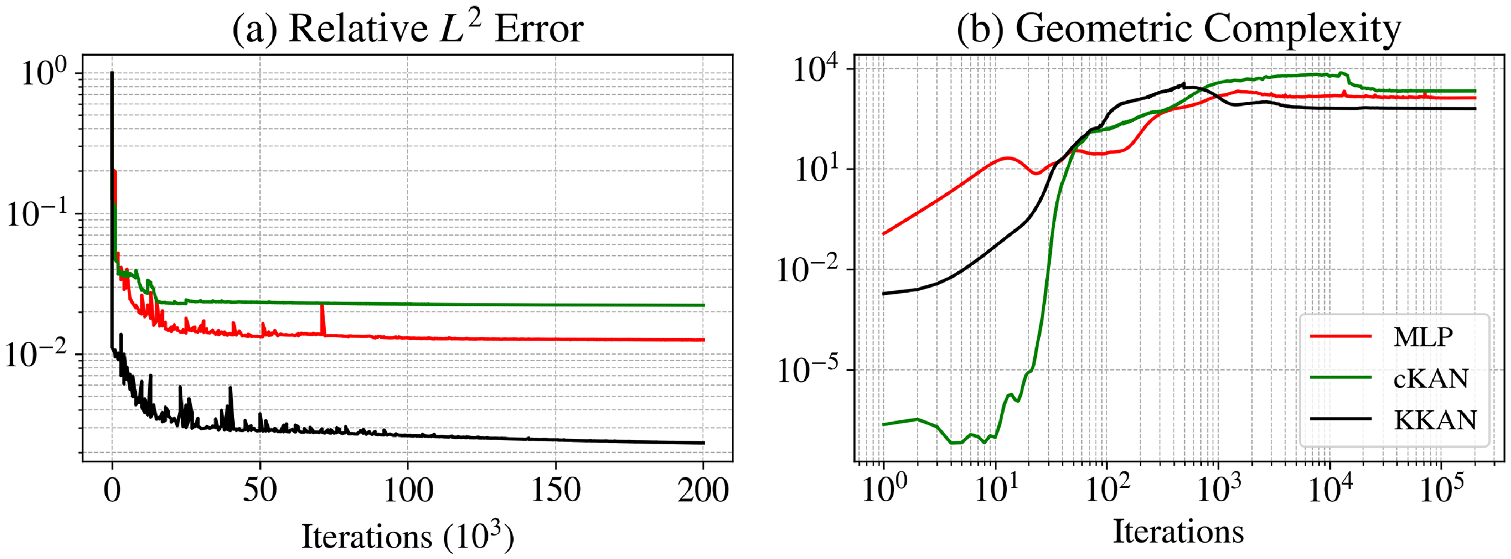
Results for discontinuous function approximation. (a) Relative $L^{2}$ error convergence on the testing dataset, evaluated on a uniform 256 × 256 mesh. KKANs converge significantly faster than MLPs, achieving a relative $L^{2}$ error of 5.86 × 10^−3^ after 200,000 ADAM iterations. cKANs converge slightly faster than MLPs initially but start to overfit after several iterations, as indicated by a sudden increase in the test error. (b) Geometric complexity evolution during training. Geometric complexity, represented by the discrete Dirichlet energy, reflects the gradient of the function with respect to its inputs. For this case, the geometric complexity is significantly higher for all models due to the two discontinuities in the function, which introduce sharp changes and amplify gradient variations. Initially, cKANs exhibit lower complexity than the other methods. However, their final complexity is significantly higher, indicating overfitting. In contrast, KKANs maintain the lowest complexity throughout training, contributing to their superior generalization and performance.

We present the results for this function in Table 2 and Fig. 4. KKANs demonstrated significantly better performance on the testing dataset, achieving a relative $L^{2}$ error of 5.86 × 10^−3^, outperforming both MLPs and cKANs. As shown in Fig. 4(a), KKANs converge significantly faster than MLP and cKAN.

As shown in Table 2, all models exhibited comparable speeds during training, with MLPs being slightly faster, averaging 2.36 ms per iteration compared to 2.64 ms for cKANs and 2.77 ms for KKANs. Fig. 4(b) highlights the evolution of geometric complexity during training. Initially, cKANs exhibit lower complexity than other methods, but their final complexity is slightly higher, potentially indicating overfitting, as lower convergence has been associated with better generalization capabilities (Dherin et al., 2022). In contrast, KKANs display the lowest complexity at the end of training, which aligns with better generalization and contributes to their robust performance. These results underscore KKANs’ ability to balance efficiency, accuracy, and robustness in approximating highly discontinuous functions.

#### 4.1.2. Smooth

In the second example, we present results for a smooth oscillatory function. This example was introduced in Ainsworth and Dong (2021) as a challenging benchmark for MLP due to the combination of frequencies. Similar to the previous case, we extended it to 2D using a tensor product formulation:

$$f\left(x_{1},x_{2}\right)=\prod_{i=1}^{2}h\left(x_{i}\right),$$

where

$$h\left(x_{i}\right)=\text{sin}\left(x_{i}\right)+\frac{1}{3}\text{sin}\left(3\pi x_{i}\right)+\frac{1}{5}\text{sin}\left(5\pi x_{i}\right)+\frac{1}{7}\text{sin}\left(7\pi x_{i}\right).$$

For the KKAN model, we use a Chebyshev-grid basis function for the outer blocks. In this example, a polynomial degree of D=15 is used to demonstrate KKANs’ ability to handle high polynomial orders effectively. In contrast, cKANs exhibit performance degradation for D>7, as noted in previous studies (Shukla et al., 2024). Specific details about this basis function, along with others, are provided in Appendix B.3.2. The relative $L^{2}$ error convergence and geometric complexity for other basis functions are shown in Fig. G.18, further demonstrating KKANs’ robustness across different configurations.

**Fig. 5.**
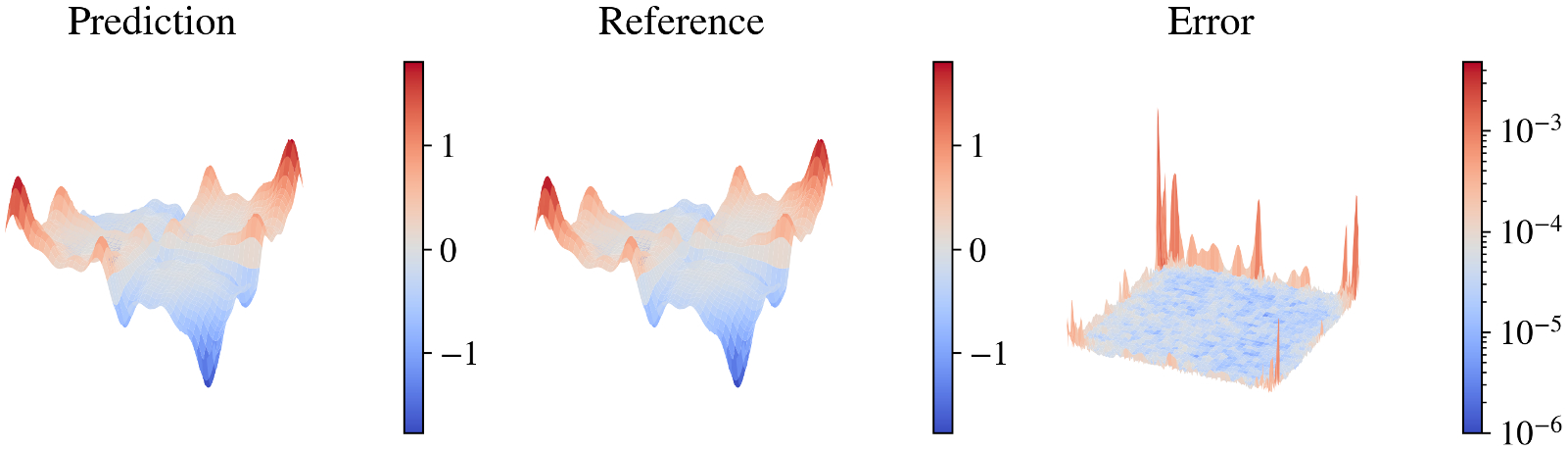
Performance of KKANs + ssRBA for smooth function approximation. Columns show predictions, ground truth references, and absolute errors, respectively. This smooth function is challenging due to its rapidly varying gradients. The inclusion of ssRBA enhances convergence and accuracy, enabling the KKAN model to achieve a relative $L^{2}$ error of 1.75 × 10^−4^.

**Fig. 6.**
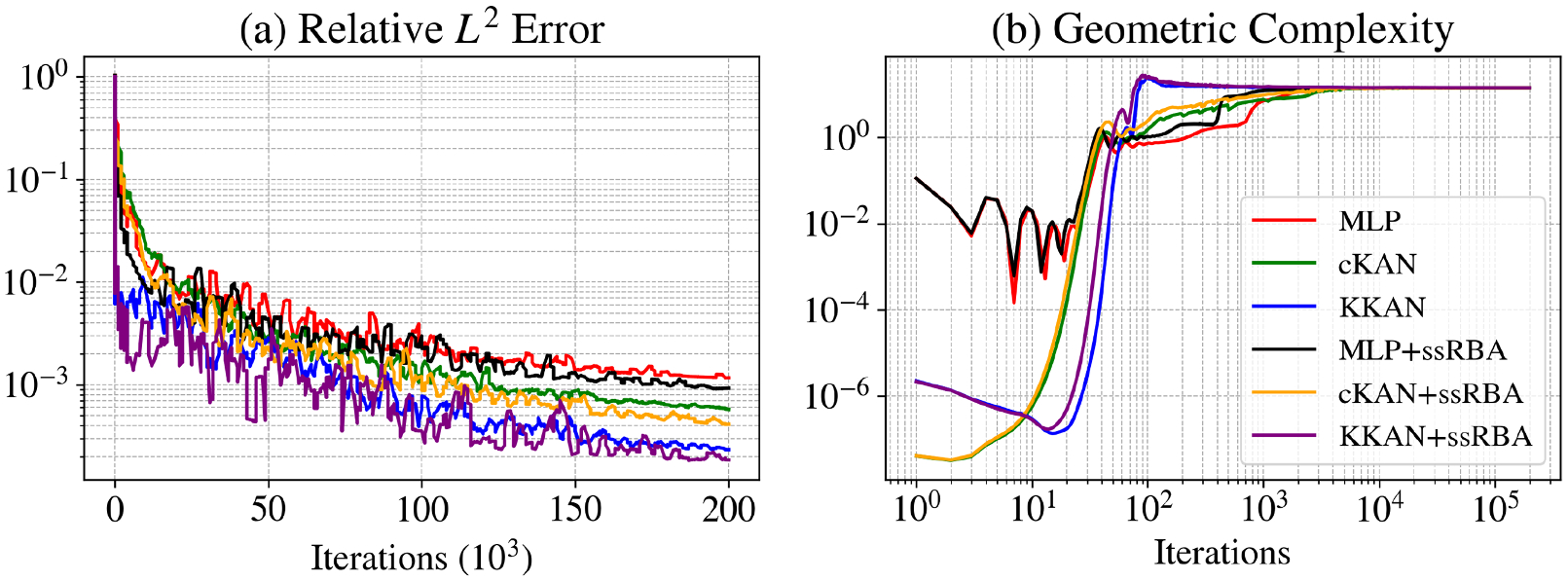
Results for smooth function approximation. (a) Relative $L^{2}$ error convergence on the testing dataset, evaluated on a uniform 256 × 256 mesh. KKANs converge significantly faster than both MLPs and cKANs. The proposed ssRBA improves the performance of all representation models by enhancing accuracy and accelerating convergence. The best-performing model, KKANs + ssRBA, achieves a relative $L^{2}$ error of 1. 75 × 10^−4^. (b) Geometric complexity evolution during training. Both cKANs and KKANs exhibit lower geometric complexity at the start of training, reflecting their simplicity in the early stages. However, by the end of training, all models converge to a similar geometric complexity, indicating a shared characteristic among all representation models.

Fig. 5 illustrates the performance of KKANs + ssRBA for smooth function approximation, showing predictions, ground truth references, and absolute errors. The smooth function, characterized by rapidly varying gradients, poses a challenge for accurate approximation. The inclusion of ssRBA significantly improves convergence and accuracy, enabling KKANs to achieve a relative $L^{2}$ error of 1.75 × 10^−4^. The relative $L^{2}$ error convergence and geometric complexity evolution during training are presented in Fig. 6. KKANs converge significantly faster than both MLPs and cKANs, with the ssRBA mechanism enhancing the performance of all models by accelerating convergence and improving accuracy. Both cKANs and KKANs start with lower geometric complexity, reflecting their simplicity in early training. However, by the end of training, all models converge to a similar geometric complexity, indicating a shared characteristic among the representations. Table 3 provides a detailed comparison, showing that KKANs consistently outperform cKANs and MLPs in accuracy, both with and without ssRBA, while maintaining comparable training times across all models.

##### Overfitting analysis.

A known trade-off in representation model design is that greater expressive power can increase the risk of overfitting, particularly in low-data regimes. To quantify this behavior for the models under consideration, we analyze the generalization gap (Hellström et al., 2025), Δε, defined as the absolute difference between the training and testing errors:

(33) $$\Delta \varepsilon =\varepsilon_{\text{test}}-\varepsilon_{\text{train}}$$

where $\varepsilon_{\text{test}}$ and $\varepsilon_{\text{train}}$ are the relative $L^{2}$ errors on the testing and training datasets, as formulated in Eq. (31). For this analysis, we used a fixed testing grid of 256 × 256 points. We then train the models on four different training set sizes, comprising approximately 5 %, 10 %, 25 %, and 50 % of the testing data points (3277, 6554, 16384, and 32,768 points, respectively), sampled independently via Latin hypercube sampling. An increasing generalization gap during training is a clear indicator of overfitting, as it signifies that the model’s performance on unseen data is degrading even as its fit to the training data improves.

**Fig. 7.**
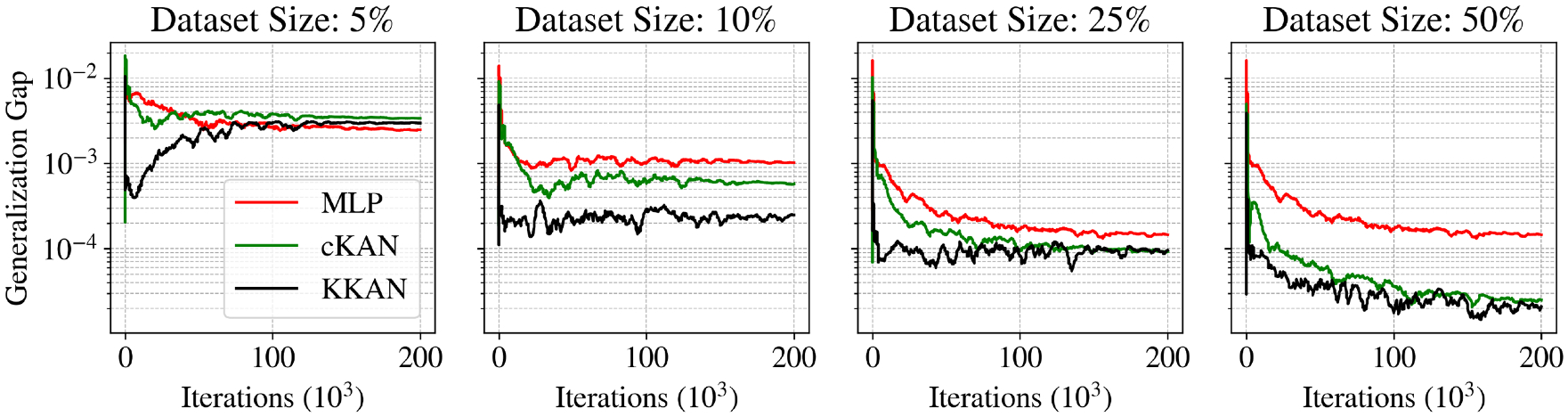
Generalization gap analysis for the smooth function approximation task, comparing MLP, cKAN, and KKAN models (columns) across varying training data sizes (rows). The gap is the absolute difference between training and testing relative $L^{2}$ errors. The results highlight that while the more expressive cKAN and KKAN models exhibit a tendency to overfit (indicated by a rising gap) in the very low-data regime (5 %), this behavior is substantially mitigated with modest increases in data (10 % and 25 %), where the generalization gap stabilizes.

The results, presented in Fig. 7, show that the more expressive KKAN and cKAN architectures exhibit a tendency to overfit when the training data is exceptionally sparse (5 % regime). However, this overfitting behavior is substantially mitigated as the amount of training data increases. With training sets of 10 % and 25 %, the generalization gap for all models stabilizes, indicating that the training and testing errors are decreasing at a comparable rate. Encouragingly, for the largest training set (50 %), the generalization gap begins to decrease, suggesting that the models are generalizing effectively.

The observed overfitting in the 5 % data regime is less of a concern for standard machine learning applications, which typically employ much larger training sets. Furthermore, in the context of PIML for forward problems, this risk is inherently lower; the governing equation in the loss function acts as a strong regularizer, and the model is not optimized on interior data points in the same manner as in a purely data-driven regression task (Toscano et al., 2025b).

### 4.2. Physics-informed machine learning

In this study, we compare the performance of KKANs, cKANs, and MLPs for PIML tasks. For KKANs, radial basis functions (RBFs) are used as the outer blocks. The PDE is trained using 25,600 collocation points, with initial conditions imposed on 201 points. Periodic boundary conditions are enforced as hard constraints through architecture modifications. The models are evaluated on a fine grid of 501 × 201, and the exact solution is obtained from established benchmark studies (McClenny & Braga-Neto, 2023). Additional implementation details and the specific network configurations are provided in Appendix F.2.

#### 4.2.1. Allen-Cahn equation

The Allen-Cahn equation is a widely recognized benchmark in Physics-Informed Machine Learning (PIML) due to its challenging characteristics (Anagnostopoulos et al., 2024b; McClenny & Braga-Neto, 2023; Raissi et al., 2019b; Wang et al., 2024a). This complexity arises from the equation’s tendency to generate solutions with sharp transitions in both spatial and temporal dimensions, which makes accurate approximation and prediction particularly difficult. The 1D Allen-Cahn partial differential equation (PDE) is defined as:

(34) $$\frac{\partial u}{\partial t}=k\frac{\partial^{2}u}{\partial x^{2}}-5u\left(u^{2}-1\right),$$

where $k=10^{-4}$. The problem is further defined by the following initial and periodic boundary conditions:

(35) $$u(0,x)=x^{2}\;\text{cos}(\pi x),\;\forall x\in [-1,1],$$

(36) u(t,x+1)=u(t,x-1),∀t≥0andx∈[-1,1].

The Allen-Cahn equation’s complexity has motivated the development of numerous techniques and methods specifically tailored for MLP-based PIML to address its inherent difficulties. Consequently, directly comparing cKANs and KKANs with MLPs presents a challenge: excluding enhancements designed for MLPs may unfairly disadvantage them, while including these techniques could potentially undermine the unique capabilities of cKANs and KKANs. Therefore, we divide our analysis into three parts.

First, to ensure a fair comparison with cKANs, which tend to degrade in performance with a higher number of parameters, we evaluate models with a reduced parameter count. Specifically, we compare the baseline versions of cKANs, MLPs, and KKANs without incorporating any additional enhancements. All models are trained using the loss function described in Eq. 19, with $\lambda_{\alpha ,i}=1$ and $m_{B}=100$, as proposed in McClenny and Braga-Neto (2023), using a batch size of 10,000 and train it for 300000 ADAM (Kingma & Ba, 2014) iterations. For KKANs, the inner block starts with a polynomial embedding layer, which enforces periodicity exactly by using odd polynomial degrees. To maintain consistency and fairness, this embedding layer is also included in the MLP and cKAN models.

The results, presented in Table 4(a), show that both KKANs and cKANs outperform MLPs in this configuration. MLP achieves a relative $L^{2}$ error of 1.36 × 10^−2^, which is significantly worse compared to cKANs (3.55 × 10^−3^) and KKANs (4.41 × 10^−3^). Furthermore, as shown in Fig. 9(a), KKANs and cKANs converge faster than MLPs, demonstrating their effectiveness in capturing the sharp transitions characteristic of the Allen-Cahn equation.

Fig. 9(b) shows the evolution of geometric complexity during training. At the beginning of training, MLPs exhibit the highest geometric complexity, while cKANs show the lowest, reflecting their initial simplicity. By the end of training, all models converge to similar geometric complexity values, suggesting that their representational capacities become comparable after sufficient training iterations. Despite this, KKANs and cKANs maintain superior accuracy, as indicated by their significantly lower $L^{2}$ errors compared to MLPs.

For the second part, we enhance the models by incorporating Fourier feature embeddings to encode periodicity across all architectures. For MLPs, we combine weight normalization (Salimans & Kingma, 2016) with the modified MLP (mMLP) (Wang et al., 2021a), resulting in an enhanced architecture referred to as weight-normalized mMLP (WNmMLP) (see Appendix C.1). Leveraging the inherent flexibility of KKANs, WNmMLP is seamlessly integrated into their inner block. In contrast, cKANs are not compatible with enhancements like mMLP or weight normalization, as current methods designed for MLPs do not directly translate to cKANs. Consequently, cKANs are implemented without these modifications, which partially explains their slightly faster training times.

For all models, we employ ssRBA-R with a batch size of 10,000. This combination of resampling and reduced parameter counts provides a speed-up of more than four times compared to standard setups. KKAN + ssRBA-R outperforms all other models, achieving a relative $L^{2}$ error of 5.5 × 10^−5^, which is comparable to state-of-the-art results (see Fig. 9(a)). As shown in Figs. G.19 and G.20, the proposed formulation effectively resamples high-error regions, enabling a double-attention mechanism via multipliers and sampling. Consequently, as depicted in Fig. 9(a), ssRBA-R accelerates convergence for all models, particularly for MLPs. Without ssRBA-R, MLPs require approximately 20,000 iterations to converge, but with this method, convergence is achieved in around 3000 iterations. Additional implementation details are described in Appendix F.2, and a ssRBA-R hyperparameter sensitivity analysis highlighting the method’s robustness is detailed in Appendix G.2.1.

Fig. 9(b) shows the geometric complexity evolution during training. While Fourier feature embeddings improve model performance, they also increase the geometric complexity for all architectures. cKANs and KKANs begin with lower geometric complexity than MLPs, demonstrating their simpler initial representations. However, all models converge to the same final complexity value, highlighting a shared representational capacity at the end of training.

For the final part of the analysis, we employ an architecture tailored to benefit MLPs. Specifically, we use larger networks and full-batch training, following the original RBA implementation (Anagnostopoulos et al., 2024b). For MLPs, we adopt the WNmMLP architecture, while for KKANs, we introduce a weight-normalized adaptive ResNet (Appendix C.2), a KKAN-specific design inspired by Wang et al. (2024a). To ensure a fair comparison, we scale the number of parameters in cKANs to approximately match those of the other architectures.

As shown in Table 4(c), with larger networks and full-batch training, even the base cKAN architecture becomes significantly slower than both MLPs and KKANs. Despite this, cKANs still demonstrate competitive accuracy. MLP + ssRBA achieves a relative $L^{2}$ error of 3.52 × 10^−5^, highlighting the effectiveness of ssRBA compared to the original RBA (Anagnostopoulos et al., 2024b), where the authors’ best-performing model achieved 4.57 × 10^−5^. Notably, KKAN + ssRBA outperforms all other models, achieving a relative $L^{2}$ error of 3.07 × 10^−5^ while also being faster than MLP + ssRBA, demonstrating its efficiency and superior accuracy. The convergence history for these three models is shown in Fig. G.22(a).

**Fig. 8.**
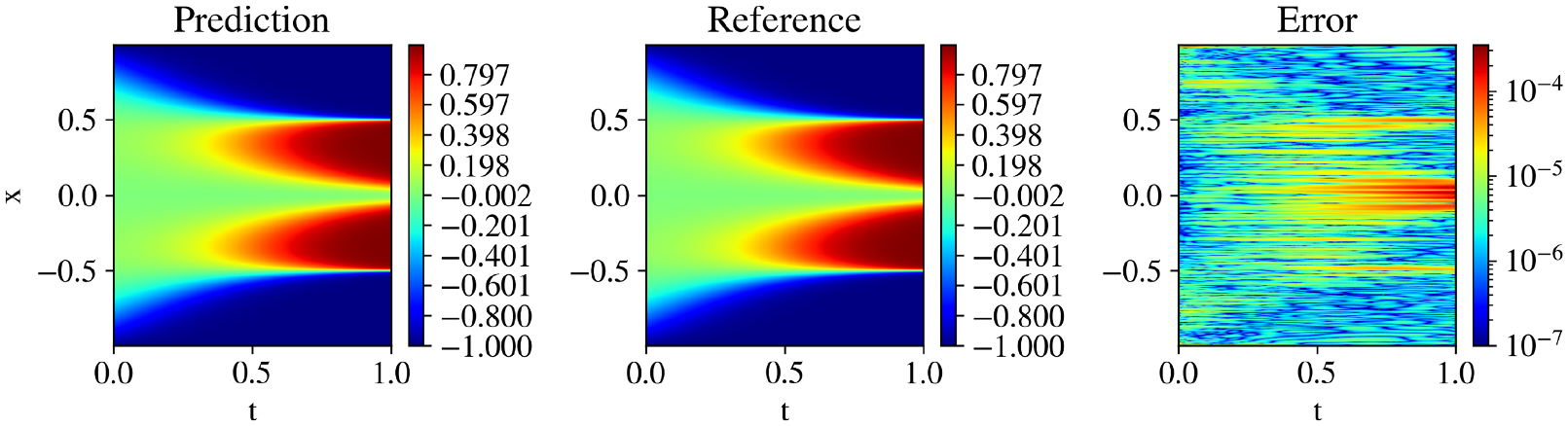
Performance of KKAN + ssRBA for solving the Allen-Cahn equation. The columns display predictions, ground truth references, and absolute errors, respectively. The model achieves a relative $L^{2}$ error of 2.28 × 10^−5^ after 500,000 Adam iterations.

**Fig. 9.**
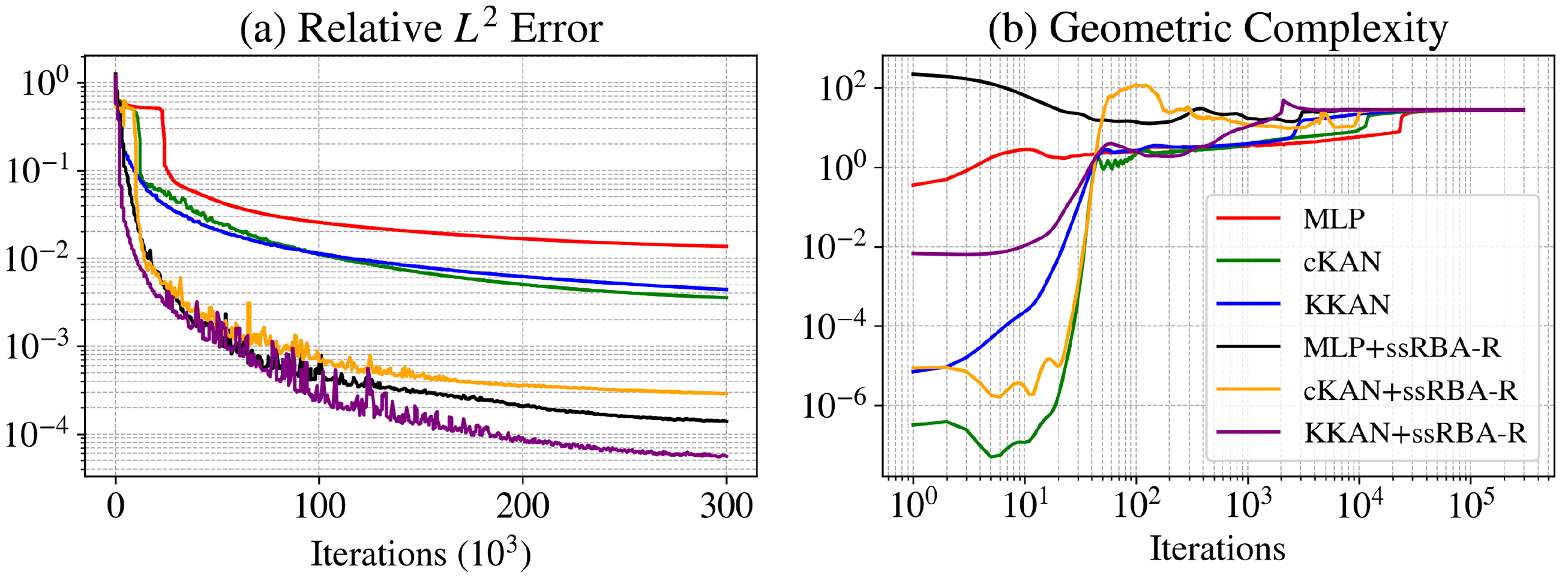
Results for solving the Allen-Cahn Equation. (a) Relative $L^{2}$ error convergence for the analyzed models. Vanilla KKAN and cKAN models converge significantly faster than MLP, which begins converging after 20, 000 Adam iterations. The inclusion of enhancements such as ssRBA and Fourier Feature embeddings (Wang et al., 2021c) accelerates convergence for all models, with KKAN + ssRBA achieving a relative $L^{2}$ error of 5.5 × 10^−5^. While larger networks could achieve better performance, the proposed approach is approximately four times faster than alternative methods. (b) Geometric complexity evolution during training. Initially, cKANs and KKANs exhibit lower complexity compared to MLPs. The Fourier Feature embeddings increase the initial complexity of the vanilla models, but as observed in previous cases, all models eventually converge to a uniform or optimal geometric complexity.

Additionally, since ssRBA allows our model to train for longer periods by maintaining a higher signal-to-noise ratio (SNR), we further trained the model for 500,000 Adam iterations, resulting in a best-performing model with a relative $L^{2}$ error of 2.28 × 10^−5^. The predictions, references, and pointwise errors are shown in Fig. 8. Furthermore, we analyzed the model’s performance across five different initializations (see Fig. G.22(b)). Additionally, the global weight, relative $L^{2}$ error, and SNR evolution for KKANs are presented in Fig. G.21.

Table 5 shows that the proposed model achieves an average relative error of 2.56 × 10^−5^ with a standard deviation of 0.17 × 10^−5^. This table compares our results with current state-of-the-art methods, demonstrating that the proposed KKAN formulation outperforms all other KAN variants and achieves performance highly comparable to fully optimized MLPs.

### 4.3. Neural operators

Finally, we evaluate the performance of MLPs, cKANs, and KKANs within operator learning frameworks. To this end, we extend the DeepONet framework (Lu et al., 2019) to KKANs, resulting in the models DeepOKKAN. These models are trained using the loss function described in Section 21. For KKAN-based models, we use radial basis functions (RBFs) as outer blocks. It is worth noting that the DeepONet framework has previously been extended to cKANs and KANs in Shukla et al. (2024) and Abueidda et al. (2024), respectively.

Additionally, we enhance our models by incorporating the QR-DeepONet framework introduced in Lee and Shin (2024). QR-DeepONet improves the standard DeepONet architecture by reparameterizing the trunk network and leveraging a QR decomposition to enhance training stability and accuracy. A detailed description of DeepONets, QR-DeepONets, and their corresponding objective functions (i.e., Eq. D.1 and D.2) is provided in Appendix D.

All models were trained on a dataset of 3500 functions and tested on 1500 functions. The QR-based models utilized a two-stage training process, with Stage 1 running for 200,000 iterations and Stage 2 for 400,000 iterations. To ensure a fair comparison, the model architectures were chosen so that the number of parameters was approximately matched across all models. Further details regarding the specific implementation and architectural choices are provided in Appendix F.

#### 4.3.1. Burgers equation

For this benchmark, we consider the 1D Burgers’ equation, which is well-known for developing sharp, shock-like gradients at low viscosity values (v):

(37) $$u_{t}+uu_{x}=vu_{xx},$$

The problem is defined on the domain Ω=(0,1)×(0,1) with periodic boundary conditions, u(t,0)=u(t,1) and $u_{x}(t,0)=u_{x}(t,1)$. Our goal is to learn the solution operator that maps an initial condition u(0,x) to the entire solution trajectory u(t,x) for all t∈[0,1]. To rigorously test model robustness, we learn this operator across three distinct scenarios: a high-viscosity case (v=1/100), an intermediate case (v=1/(100π)), and a challenging low-viscosity case (ν=1/1000). Initial conditions are sampled from a Gaussian random field (GRF), and the corresponding numerical solutions are obtained using the code provided in Wang et al. (2021b), which implements a high-fidelity spectral solver based on the conservative form of the equation.

**Fig. 11.**
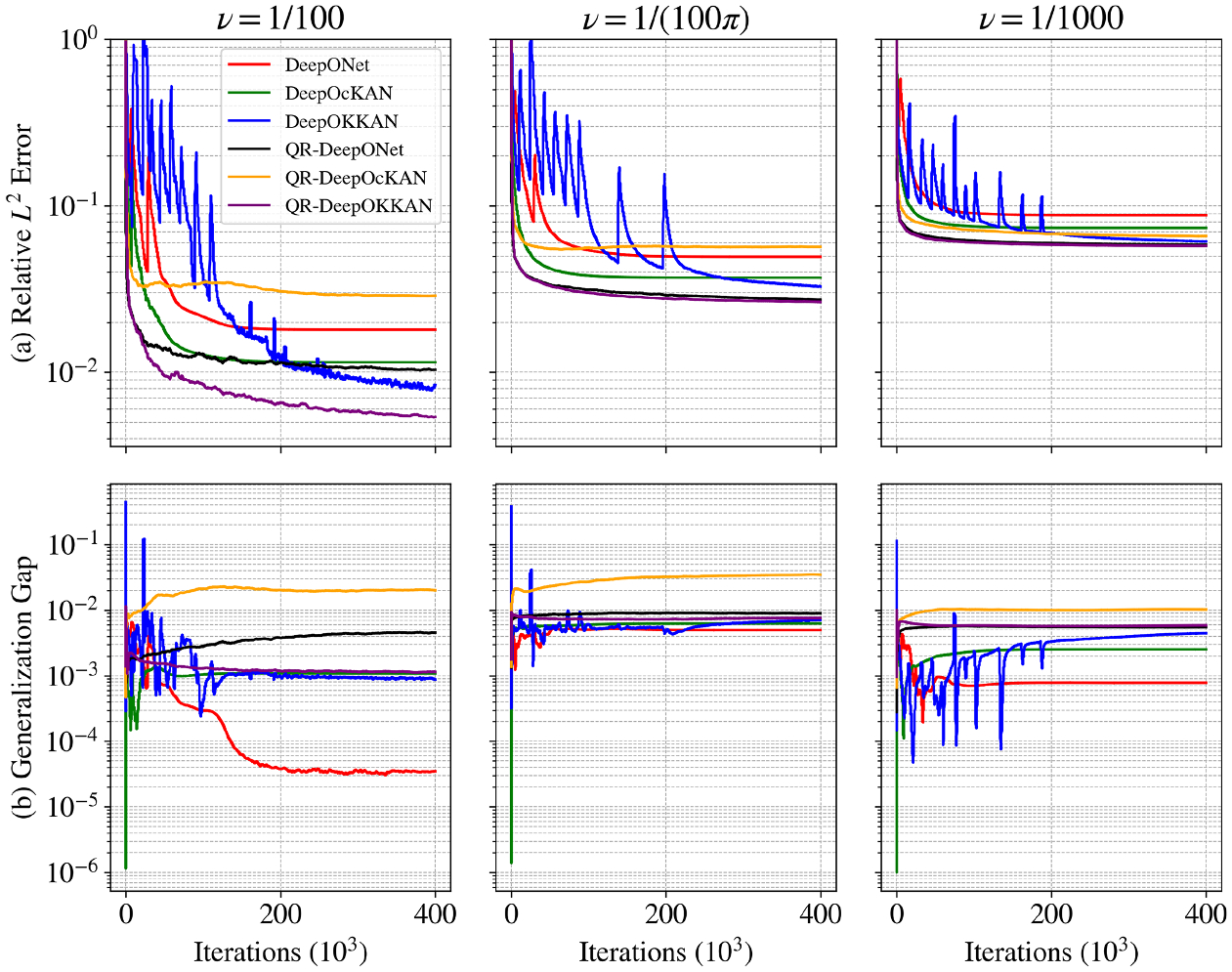
Comparison of convergence history for all operator learning models across three different viscosity values (v). (a) Relative $L^{2}$ error on the testing dataset. As expected, the performance of all models deteriorates as the viscosity is reduced, making the problem more challenging. For the baseline models, the proposed DeepOKKAN consistently outperforms DeepONet and DeepOcKAN across all viscosities. The QR reparameterization enhances the performance of the KKAN and MLP-based models but is detrimental to the cKAN model. Among all models, QR-DeepOKKAN and QR-DeepONet are the top performers, with QR-DeepOKKAN being the most accurate in the high-viscosity case. (b) Generalization gap, defined as the difference between the testing and training relative errors $\left(\varepsilon_{\text{test}}-\varepsilon_{\text{train}}\right)$. An increasing gap is clear evidence of overfitting. The poor performance of QR-DeepOcKAN is explained by its large and increasing generalization gap in all scenarios. We also observe evidence of overfitting for the baseline DeepOKKAN model in the most challenging low-viscosity case (v=1/1000).

The performance of all operator learning models is summarized in Fig. 11 and detailed in Table 6. As expected, reducing the viscosity increases the problem’s difficulty, leading to higher prediction errors across all models. In a direct comparison of the baseline architectures, our proposed DeepOKKAN consistently outperforms both the standard DeepONet (MLP-based) and DeepOcKAN (cKAN-based) across all viscosity regimes. The introduction of the QR reparameterization significantly boosts the performance of the KKAN and MLP-based models. However, this enhancement increases the risk of overfitting and is detrimental to the cKAN architecture. To analyze this behavior, we analyze the generalization gap, which measures the difference between the training and testing errors (see Eq. 7) and is used as a measure of overfitting. Using this metric, we observed that QR-DeepOcKAN exhibits severe overfitting, as evidenced by its large and continuously increasing generalization gap as shown in Fig. 11(b). We also observe signs of overfitting for the baseline DeepOKKAN model in the most challenging low-viscosity setting. This indicates that while KAN architectures are more expressive, they are prone to overfitting.

Our proposed models demonstrate a high degree of accuracy and successfully predict the entire solution history, u(x,t). In the high-viscosity case (v=1/100), our best model yields a relative $L^{2}$ error of 0.52 %, which is a marked improvement over the 3.3 % reported by the physics-informed approach in Wang et al. (2021b). For v=1/(100π), our QR-DeepOKKAN’s error is 2.64 %, outperforming a comparable data-driven method that reported 3.02 % for only the final state (Shukla et al., 2024). In the most challenging low-viscosity regime (v=1/1000), our model’s error of 5.74 % remains highly competitive with the 3.4 % achieved by specialized, physics-informed MLP-based models (Chen et al., 2024).

To complement the quantitative metrics, we present qualitative results in Figs. G.24, G.25, and 10. Each figure displays the model’s prediction, the ground truth reference, and the pointwise absolute error for a representative test sample, corresponding to the high-viscosity $\left(\nu =\frac{1}{100}\right)$, intermediate ($\nu =\frac{1}{100\pi }$), and low-viscosity ($\nu =\frac{1}{1000}$) regimes. The close agreement between the predicted and true solutions, especially in capturing the sharp shock front in the challenging low-viscosity case, corroborates the robustness of the proposed KKANs.

## 5. Learning dynamics via information bottleneck theory

The learning dynamics of a model can be characterized by analyzing the signal-to-noise ratio (SNR) of backpropagated gradients (i.e., Eq. 24). The SNR provides insight into deterministic and stochastic regimes during training, where high SNR corresponds to deterministic updates, and low SNR indicates stochastic exploration (Anagnostopoulos et al., 2024a). By studying the variation in SNR, we can identify distinct stages of learning: fitting, transition, and diffusion. These stages have been observed in PIML tasks for MLP and cKAN models, and in this study, we extend the framework to KKANs for function approximation and operator learning tasks.

**Fig. 12.**
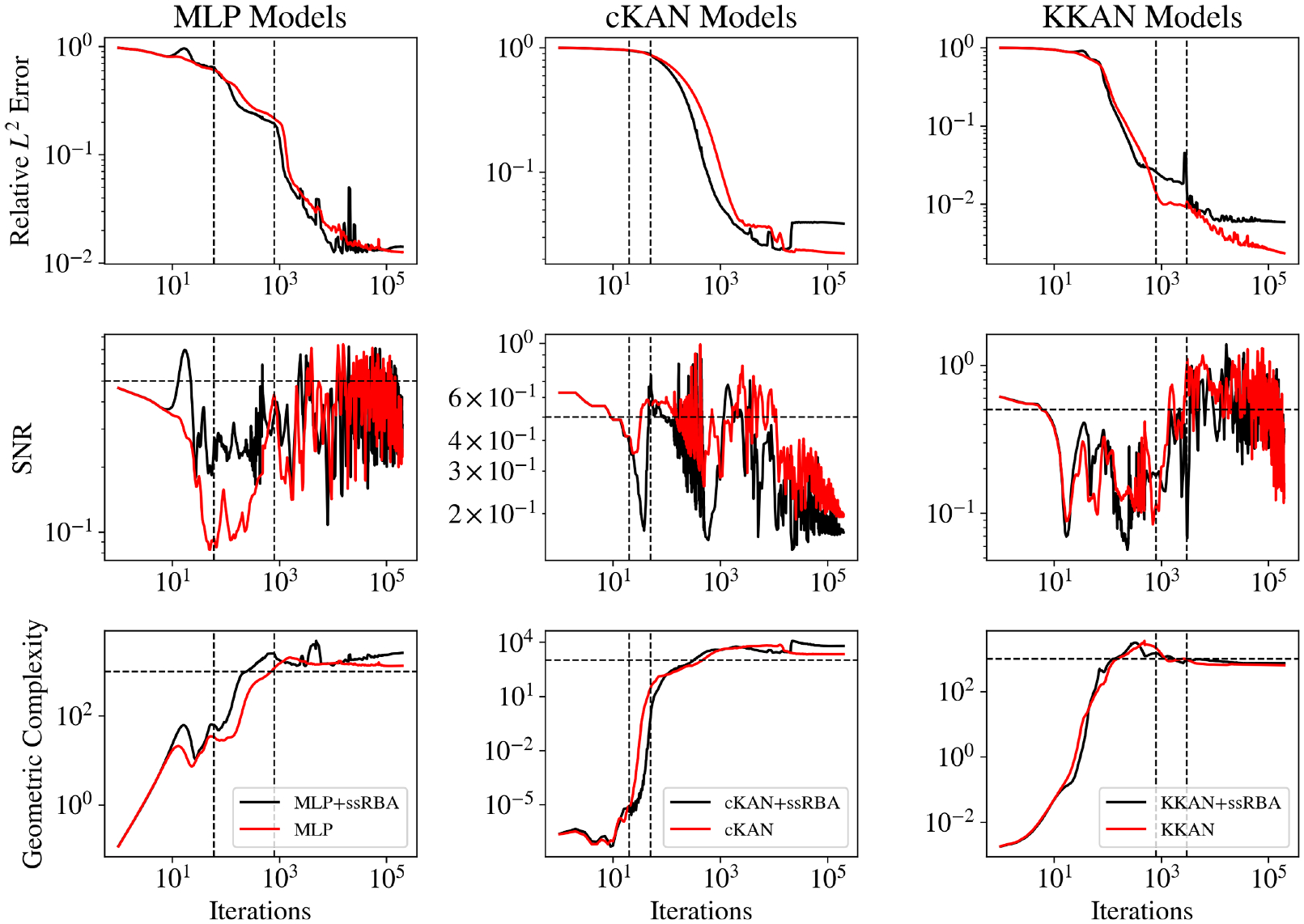
Relative $L^{2}$ error (first row), SNR (second row), and geometric complexity (third row) convergence for discontinuous function approximation using MLP (first column), cKAN (second column), and KKAN models (third column). The three stages of learning are observed across all representation models and are marked, for the worst performing case, by vertical dashed lines. During the fitting stage, the SNR decreases without significant improvement in the generalization error (i.e., relative $L^{2}$ error) and with an increase in geometric complexity. In the transition stage, the model explores the optimal direction, resulting in further increases in geometric complexity. In the diffusion stage, the model achieves the optimal complexity and finds the optimal direction, leading to an increase in SNR and significant convergence. However, it can also be observed that, over time, the SNR decreases again. For extreme cases such as cKAN, this decrease in SNR leads to higher geometric complexity, causing the model to overfit and harming the generalization performance.

**Fig. 13.**
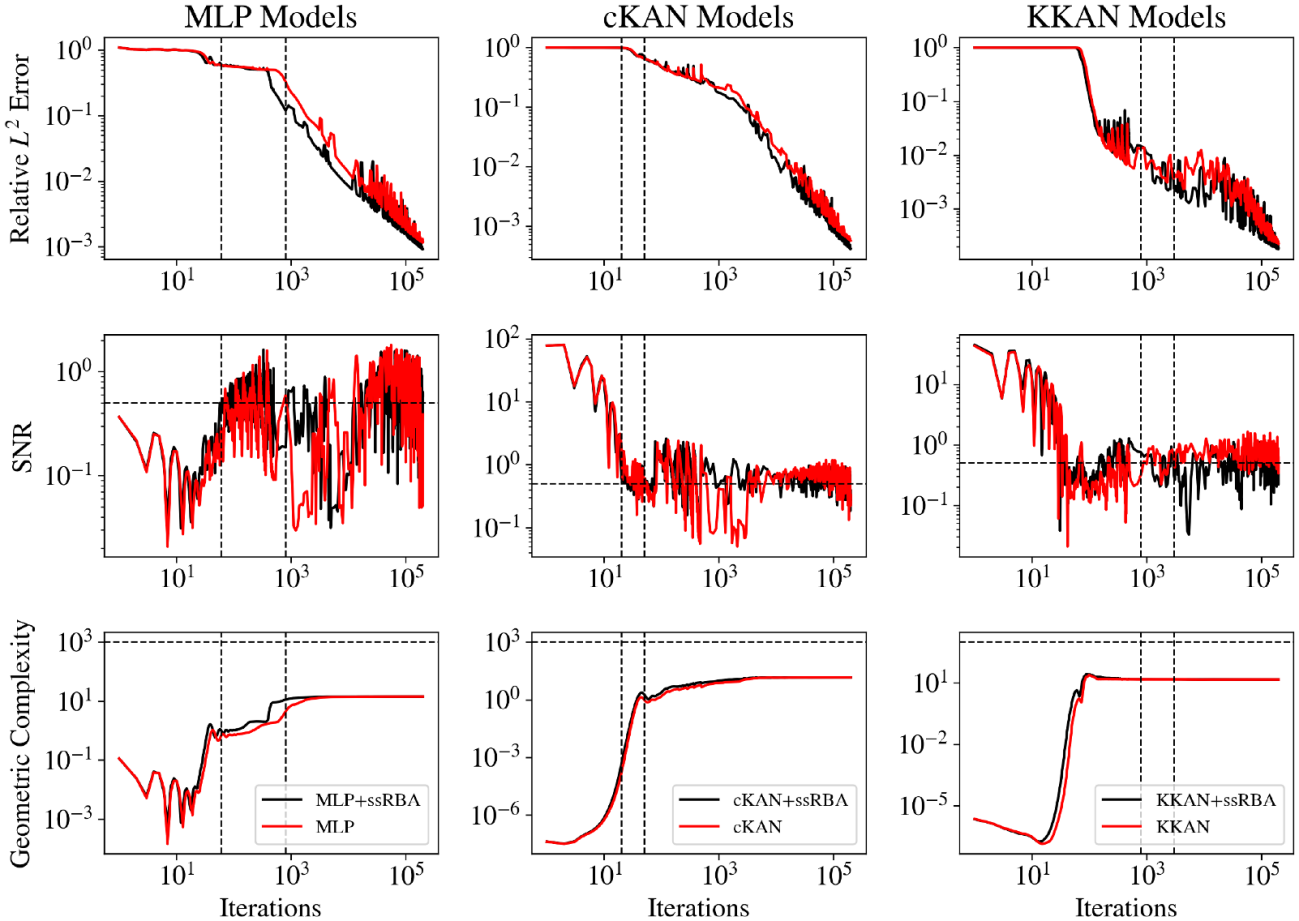
Relative $L^{2}$ error (first row), SNR (second row), and geometric complexity (third row) convergence for smooth function approximation with MLP (first column), cKAN (second column), and KKAN models (third column). The three stages of learning are evident across all models and are indicated, for the worst-performing case, by vertical dashed lines. During the fitting stage, the SNR decreases, accompanied by a slight improvement in the generalization error (i.e., relative $L^{2}$ error) and increased geometric complexity. Notably, for MLPs, the geometric complexity is initially high and decreases before further increasing again. In the transition stage, the model explores the optimal direction for learning, which results in an increase in geometric complexity. Finally, in the diffusion stage, the model achieves the optimal complexity and direction, causing the SNR to rise and driving significant convergence. For smooth functions, maintaining a high SNR is easier, which supports continuous learning and improved performance.

**Fig. 14.**
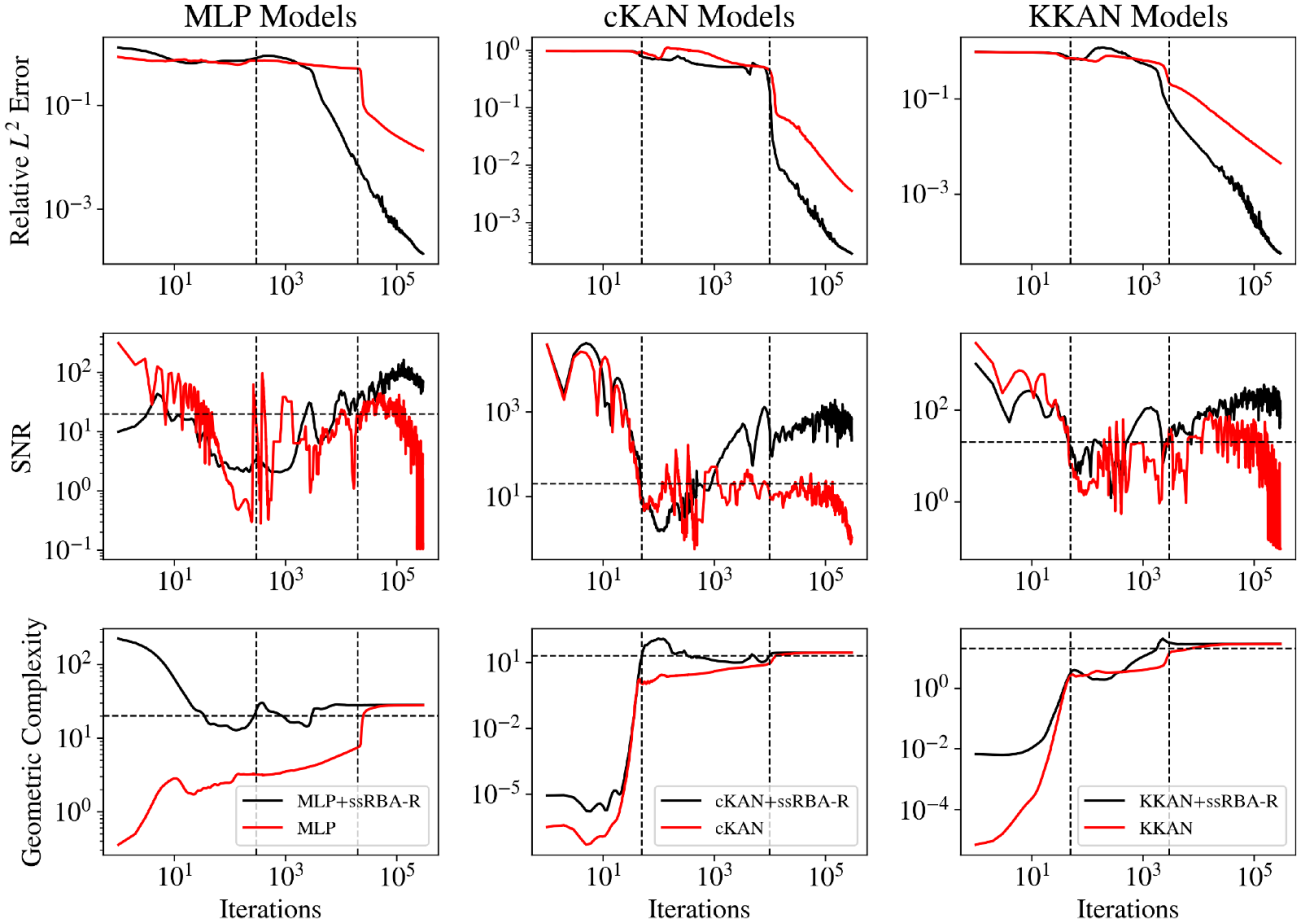
Relative $L^{2}$ error (first row), SNR (second row), and geometric complexity (third row) convergence for solving the Allen-Cahn Equation using MLP (first column), cKAN (second column), and KKAN models (third column). The three stages of learning are evident across all models and are marked, for the worst-performing case, by vertical dashed lines. During the fitting stage, the SNR decreases, accompanied by minor improvements in the generalization error (i.e., relative $L^{2}$ error) and an increase in geometric complexity. As discussed in the results section, the mMLP increases the geometric complexity of the models. Notably, MLP + ssRBA-R exhibits a significantly high geometric complexity, where the model first simplifies the representation, characterized by fluctuations in the SNR. In the transition stage, the model explores the optimal direction for learning, resulting in a further increase in geometric complexity. However, during this stage, the generalization error does not improve. Finally, in the diffusion stage, the model achieves the optimal complexity and direction, leading the SNR to converge to an optimal value and driving significant improvements in generalization error. It is noteworthy that the proposed ssRBA-R successfully maintains a consistently high SNR, resulting in optimal performance across all representation models.

**Fig. 15.**
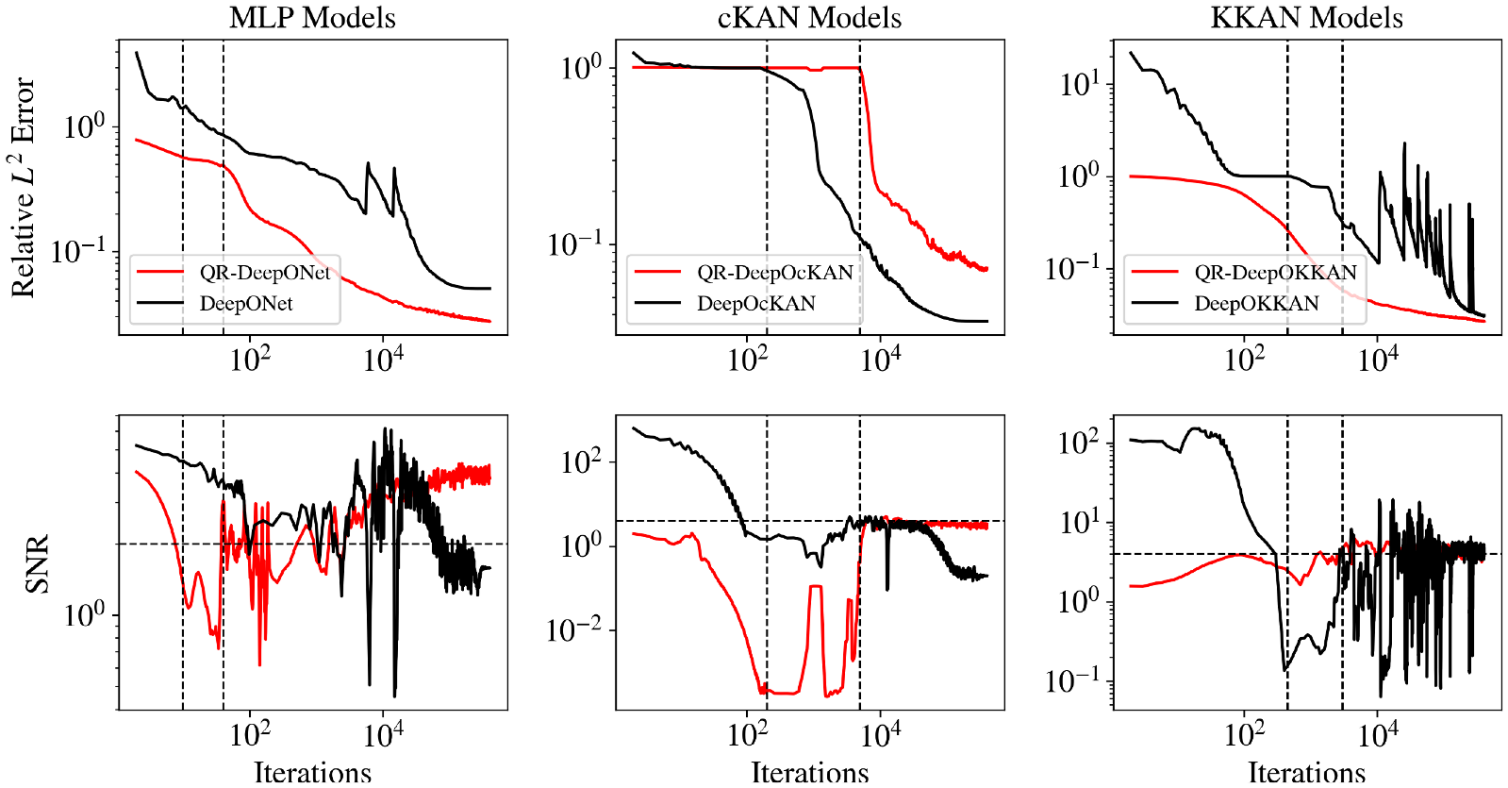
Relative $L^{2}$ error (first row) and SNR (second row) for operator learning tasks using MLP (first column), cKAN (second column), and KKAN models (third column). Due to the high dimensionality of the branch net inputs (100), computing the geometric complexity is computationally challenging. Therefore, only the relative error and SNR results are presented. The three stages of learning are evident across all models, with vertical dashed lines indicating the stages for the worst-performing case. During the fitting stage, the SNR decreases, accompanied by minor improvements in the generalization error (i.e., relative $L^{2}$ error). In the transition stage, the model explores the optimal direction, with no significant improvement in generalization error. Finally, in the diffusion stage, the model identifies the optimal direction, leading to an increase in SNR and driving significant convergence.

In this study, we extend the Information Bottleneck (IB) framework to KKANs as well as function approximation and operator learning tasks. Notably, we experimentally observed that the three stages of learning are present across all tasks, including function approximation (Figs. 12 and 13), PIML (Fig. 14), and operator learning (Fig. 15), for all representation models: MLP (first column), cKAN (second column), and KKAN (third column). The SNR is closely linked to the generalization error shown in the first row of all our figures. Additionally, we observed an intriguing connection between the SNR and the geometric complexity, as depicted in the third row of all the figures.

In particular, the three stages of training are described as follows:

### Fitting.

The initial phase of training, where gradients are large and agreement between subdomains is high, resulting in a high SNR. During this deterministic phase, the model focuses on reducing the training error across subdomains. However, as the model learns the “general” trend, the disagreement between subdomains increases, which leads to a low SNR. Therefore, this stage can be identified by a transition from high to low SNR.

The fitting stage can be clearly identified in all our examples, as shown in the second row of Figs. 12–15. During this stage, the training errors are reduced; however, there is minimal improvement in the relative error on the testing dataset (see the first row of Figs. 12–15. Additionally, during this stage, as the model fits new information, it becomes more complex, leading to an increase in geometric complexity. Notice that if the geometric complexity is high at the beginning of training (as seen in MLP in Figs. 13 and 14), the model first needs to simplify these representations; this may justify why better initialization schemes are characterized by lower initial geometric complexities (Dherin et al., 2022).

### Transition.

After the model fits the data, it enters an exploration stage where it attempts to minimize the error across all subdomains. During this exploratory stage, there is disagreement on the optimal direction for weight updates, resulting in a low SNR. This phase is also characterized by a minor or null decrease in the generalization error (see Figs. 12–15 (first row)). Additionally, as the model attempts to minimize the error along all subdomains, the geometric complexity increases (see Figs. 12–15 (third row)).

### Diffusion.

After the exploration phase, the model becomes sufficiently complex (i.e., after the geometric complexity increases) to reach an agreement on the optimal update direction, leading to a sudden increase in the SNR. Once this optimal direction (i.e., high SNR) is achieved, the generalization error improves significantly, and the geometric complexity decreases as the model becomes more efficient and starts simplifying internal representations. However, as the loss decreases further, the gradients become smaller (i.e., lower signal), which leads to a subsequent decrease in the SNR—eventually, the SNR drops, learning stops, and the generalization error plateaus. This pattern is clearly observed in our operator learning tasks (see Fig. 15). For complex problems such as discontinuous function approximation (see Fig. 12), this decrease in SNR induces an increase in the geometric complexity, which eventually causes the model to overfit. On the other hand, for smooth function approximation tasks, the SNR remains higher, enabling a continuous decrease in the generalization error without overfitting (see Fig. 13). Notice that the ss-RBA introduced for PIML tasks (Section 3.5) successfully maintains a high SNR during the late diffusion stage, enabling continuous and improved learning (see Fig. 14).

Interestingly, during the diffusion stage, all models converge to the same geometric complexity. This behavior is expected because the geometric complexity (Eq. 32) depends solely on the Jacobian of the function, $\nabla u_{x}$. Since u is unique, its derivatives with respect to the inputs are also identical, leading to the same geometric complexity across models. Notice that for discontinuous function approximation (see Fig. 12), the geometric complexity is higher due to the undefined derivatives at the discontinuities. However, this metric proves useful for identifying unnecessarily complex functions, particularly for spatial data or operator learning tasks, such as function approximation. Similarly, in PIML, where the function is learned through its residuals, higher geometric complexity corresponds to larger $\nabla u_{x}$ values when evaluated at the testing points, potentially indicating overfitting (Dherin et al., 2022).

## 6. Summary

The Kolmogorov-Arnold representation theorem (KART) has historically faced skepticism regarding its practical utility, with criticisms citing its computational complexity and infeasibility for direct implementation. Inspired by Ku̇rková’s reinterpretation of KART, we proposed the Ku̇rkov’a-Kolmogorov-Arnold Networks (KKANs), a novel two-block architecture that adheres closely to the original theorem. KKANs combine robust MLP-based inner blocks with interpretable basis functions as outer blocks, preserving both computational efficiency and theoretical rigor. We proved that KKANs are universal approximators regardless of the choice of basis functions and for a general class of functions. Also, we extended KKAN’s applicability to Physics-Informed Machine Learning (PIML) and operator learning tasks. We evaluated our models’ performance using their generalization error (i.e., relative error on testing data), geometric complexity, and learning dynamics via the Information Bottleneck (IB) theory.

To improve PIML models’ performance, we developed a new optimization method for PIML called self-scaled residual-based attention (ssRBA). This method induces uniform convergence and enhances learning dynamics, enabling prolonged learning. Additionally, we proposed a global weighting scheme that scales loss weights based on their gradients, ensuring consistent update directions that minimize all loss terms.

In function approximation tasks, we showed that KKANs consistently outperformed MLPs and cKANs across both smooth and discontinuous function benchmarks. KKANs exhibited faster convergence and superior generalization, maintaining lower geometric complexity throughout training. In contrast, cKANs showed signs of overfitting, with higher final geometric complexity and deteriorating generalization performance. These results highlight KKANs’ ability to efficiently handle both smooth and discontinuous functions while preserving robustness and adaptability.

For PIML tasks, we demonstrated that KKANs offered significant computational advantages while achieving excellent accuracy, particularly when enhanced with ssRBA. KKANs converged faster than alternative methods while maintaining high generalization capabilities. By maintaining a high SNR during the diffusion stage, ssRBA enabled prolonged training and improved learning efficiency, positioning KKANs as strong competitors to state-of-the-art PIML models.

In operator learning tasks, we extended the DeepONet formulation to KKANs and the QR-DeepONet formulation to cKANs and KKANs. The QR formulation stabilized training and enhanced KKAN and MLP performance, improving accuracy and robustness. However, we observed that the QR approach negatively impacted cKANs, revealing their sensitivity to optimization techniques. KKANs consistently demonstrated adaptability and superior performance, making them suitable for a wide range of operator learning applications.

We analyzed the learning dynamics of all the analyzed models using the Information Bottleneck (IB) theory, extending its application to function approximation and operator learning tasks. We demonstrated that the three stages of learning—fitting, transition, and diffusion—are universal across models and tasks. Additionally, we identified a strong relationship between geometric complexity and SNR, providing deeper insights into learning dynamics. In summary, we observed that during the fitting stage, the model captures general trends, transitioning from high to low SNR as geometric complexity converges to a structured representation. In the transition stage, the model explores subdomains to minimize errors, but the stochastic nature of this phase keeps SNR low while geometric complexity increases gradually.

In the diffusion stage, we observed that once the model becomes complex enough, it identifies the optimal update direction, resulting in a sharp increase in SNR. This stage is critical for reducing generalization error, as the geometric complexity converges to an optimal value unique to the learned solution. However, as gradients weaken and SNR decreases, overfitting can occur in complex tasks like discontinuous function approximation. To address this issue, we used ssRBA to dynamically scale local multipliers during the diffusion stage, maintaining a high SNR. This approach enabled prolonged learning and ensured robust performance for PIML tasks.

In conclusion, we propose KKANs as a transformative approach to implementing KART-based architectures in scientific machine learning. By closely adhering to the Kolmogorov-Arnold theorem and integrating advancements like ssRBA, KKANs bridge the gap between interpretability and computational efficiency. Our success across function approximation, PIML, and operator learning tasks establishes KKANs as a versatile and powerful tool, paving the way for future research and innovation in scientific machine learning.

## Supplementary Material

1

## Figures and Tables

**Fig. 10.**
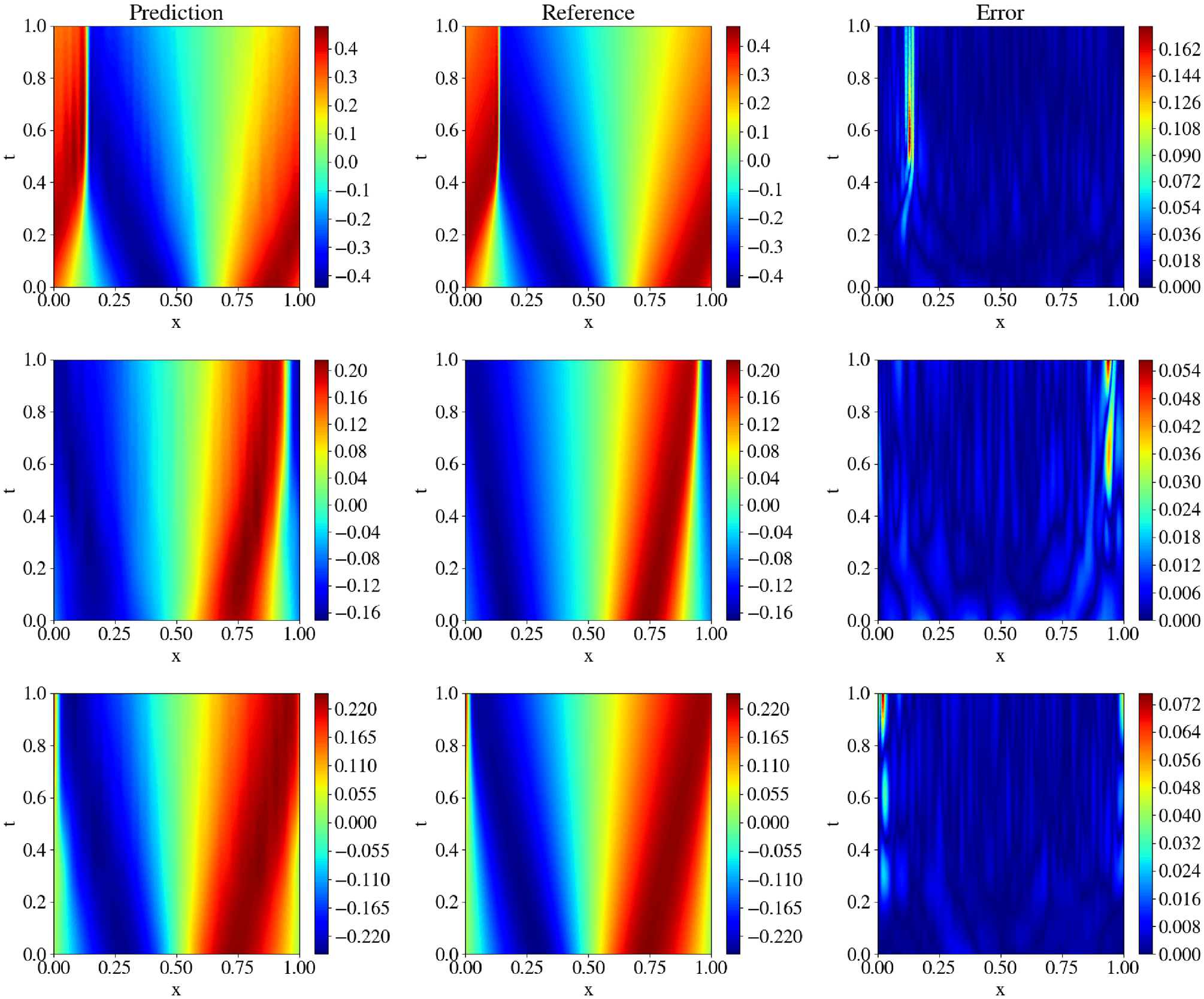
QR-DeepOKKAN predictions for three different initial conditions from the testing dataset for Burgers’ Equation with v=(1/1000). The corresponding relative $L^{2}$ errors are: (top row) 4.84 × 10^−2^, (middle row) 4.63 × 10^−2^, and (bottom row) 3.17 × 10^−2^.

**Table 1:** KART and some selected variants.

Version	Representation	Inner Function	Outer Function
KART (1957)	$\overset{2d}{\underset{q=0}{\sum }}g_{q}\left(\overset{d}{\underset{p=1}{\sum }}\psi_{p,q}\left(x_{p}\right)\right)$	$\frac{d(2d+1)}{\psi_{p,q}\in C([0,1])}$	$\frac{2d+1}{g_{q}\in C(\bar{R})}$
Lorentz (1962)	$\overset{2d}{\underset{q=0}{\sum }}g\left(\overset{d}{\underset{p=1}{\sum }}\lambda_{p}\psi_{q}\left(x_{p}\right)\right)$	$\frac{2\;d+1}{\psi_{q}\in {\text{Lip}}^{\alpha };\in [0,1];↗}$	$\frac{1}{g\in C([0,d])}$
Sprecher (1965)	$\overset{m}{\underset{q=0}{\sum }}g_{q}\left(\overset{d}{\underset{p=1}{\sum }}\lambda_{p}\psi \left(x_{p}+qa\right)\right)$	$\frac{1}{\psi \in Lip^{{\text{log}}_{\gamma }2};[0,2]\to [0,2];↗}$	$\frac{m+1}{g_{q}\in C\left(\left[0,2\frac{\gamma -1}{\gamma -2}\right]\right)}$
Braun (2009)	$\overset{2d}{\underset{q=0}{\sum }}g\left(\overset{d}{\underset{p=1}{\sum }}\lambda_{p}\psi \left(x_{p}+qa\right)+c_{q}\right)$	$\frac{1}{\psi \in C(\mathbb{R});↗}$	$\frac{1}{g\in C(R)}$
Schmidt-Hieber (2021)	$\frac{g\left(\sum_{p=1}^{d}3^{1-p}\psi \left(x_{p}\right)\right)}{f:\beta \text{-smooth},\beta \in (0,1]}$	$\frac{1}{\psi \in C:[0,1]\to C;↗}$	$\frac{1}{g:𝒞\to \mathbb{R};\alpha \text{-smooth}}$
Ismayilova & Ismailov (2024)	$\frac{\frac{\text{Formula as in Braun}\left(2009\right)}{\text{with given}\;a\left(\gamma \right),\lambda_{p}\left(\gamma \right)}}{c_{q}=(2\;d+1)\;q}$	$\frac{1}{\psi \in {\text{Lip}}^{{\text{log}}_{\gamma }2};[0,2]\to [0,2];↗}$	$\frac{\frac{f\in C\Rightarrow g\in C}{f\text{dis}-C\Rightarrow g\text{dis}-C}}{\|f{\|}_{\infty },\|g{\|}_{\infty }=\infty }$

**Table 2:** Comparison of models for function approximation of a discontinuous function, evaluated on the number of parameters (|θ|), training time (t), and relative $L^{2}$ error. KKANs significantly outperform both MLPs and cKANs. The training times are comparable across models. The challenging nature of the discontinuous function, characterized by abrupt changes, amplifies the difficulty for all models, highlighting KKANs’ robustness and ability to generalize better under such conditions.

Model	$|\theta |\left(10^{3}\right)$	$t\left(\frac{ms}{it}\right)$	$RL^{2}$ Error
MLP	41	2.36	1.26×10^−2^
cKAN	39	2.64	2.22×10^−2^
**KKAN**	40	2.77	**5.86**×**10**^−**3**^

**Table 3:** Comparison of models for smooth function approximation, evaluating the number of parameters (|θ|), training time (t), and relative $L^{2}$ error. The performance of all models improves with the addition of ssRBA. The training times are comparable across models. KKANs consistently outperform cKANs and MLPs in accuracy, both with and without ssRBA, demonstrating their robustness and efficiency for smooth function approximation.

Model	$|\theta |\left(10^{3}\right)$	$t\left(\frac{ms}{it}\right)$	$RL^{2}$ Error
MLP	41	2.38	1.17 × 10^−3^
cKAN	46	3.23	5.75 × 10^−4^
KKAN	41	2.88	2.234 × 10^−4^
MLP + ssRBA	50	2.32	9.14 × 10^−4^
cKAN + ssRBA	46	3.26	4.17 × 10^−4^
**KKAN + ssRBA**	41	3.09	**1.74 × 10** ^−**4**^

**Table 4:** Comparison of representation models for the Physics-Informed solution of the Allen-Cahn equation. The models are evaluated based on the number of parameters (|θ|), time per iteration (t), and the Relative $L^{2}$ Error.

	Model	$|\theta |\left(10^{3}\right)$	$t\left(\frac{ms}{it}\right)$	$RL^{2}$ Error
a	MLP	21	6.75	1.36 × 10^−2^
	cKAN	20	6.37	3.55 × 10^−3^
	KKAN	20	6.75	4.41 × 10^−3^
b	MLP + ssRBA-R	25	6.29	1.37 × 10^−4^
	cKAN + ssRBA-R	23	5.15	2.87 × 10^−4^
	KKAN + ssRBA-R	22	6.80	5.50 × 10^−5^
c	MLP + ssRBA	92	26.85	3.52 × 10^−5^
	cKAN + ssRBA	107	31.71	2.91 × 10^−4^
	**KKAN + ssRBA**	118	21.36	**3.07 × 10** ^−**5**^

**Table 5:** Comparison of state-of-the-art methods for solving the Allen-Cahn equation using various representation models, enhancements, and boundary conditions (B.C.). The table includes relative $L^{2}$ errors for models trained with periodic and Dirichlet boundary conditions. MLP-based methods demonstrate strong performance, with BRDR+ achieving the best result among MLPs. The proposed KKAN + ssRBA formulation outperforms all KAN-based formulations and demonstrates highly competitive accuracy compared to state-of-the-art MLPs.

Model	Enhancements	B. C.	$RL^{2}$ Error
MLP	SA (McClenny & Braga-Neto, 2023)	Periodic	(1.51 ± 2.76) × 10^−4^
MLP	RBA (Anagnostopoulos et al., 2024b)	Periodic	(5.80 ± 0.74) × 10^−5^
**MLP**	**BRDR**+ (Chen et al., 2024)	Periodic	**(1.45 ± 0.46) × 10** ^−**5**^
MLP	PirateNets (Wang et al., 2024a)	Periodic	2.24 × 10^−5^
KAN	AcNet (Guilhoto & Perdikaris, 2024)	Dirichlet	6.80 × 10^−5^
KAN (Liu et al., 2024c)	(Guilhoto & Perdikaris, 2024)	Dirichlet	5.30 × 10^−4^
KAN (Liu et al., 2024c)	(Shukla et al., 2024)	Dirichlet	5.65 × 10^−2^
KKAN	ssRBA (Ours)	Periodic	(2.56 ± 0.17) × 10^−5^

**Table 6:** Comparison of different operator learning models for the Burgers’ equation, evaluating the number of parameters (|θ|), prediction accuracy (relative $L^{2}$ error), and computational time across three viscosity values (v). The proposed QR-DeepOKKAN model demonstrates robust and superior performance across the different problem difficulties. For the high-viscosity case (v=1/100), our best model achieves a 0.52 % error, significantly outperforming the 3.3 % error reported for a physics-informed approach in Wang et al. (2021b). For v=1/(100π), our QR-DeepOKKAN achieves a 2.64 % error on the full solution field, which is more accurate than results from a comparable data-driven setup that reported a 3.02 % error on only the final time step (Shukla et al., 2024). In the most challenging low-viscosity case (v=1/1000), our model’s error of 5.74 % is competitive with the 3.4 % error from a specialized physics-informed solver (Chen et al., 2024). Notably, unlike prior studies that focused on specific instances or time steps, our operator learning framework predicts the entire solution history, u(x,t) for t∈[0,1].

v	Model	$|\theta |\left(10^{3}\right)$	$S1\left(\frac{ms}{it}\right)$	$S2\left(\frac{ms}{it}\right)$	$RL^{2}$ Error (10^−2^)
$\frac{1}{100}$	DeepONet	132	5.21		1.80
	DeepOcKAN	108	5.26		1.14
	DeepOKKAN	156	7.78		0.76
	QR-DeepONet	482	3.37	3.63	5.70
	QR-DeepOcKAN	461	3.35	3.15	2.88
	**QR-DeepOKKAN**	496	4.32	4.29	**0.52**
$\frac{1}{100\pi }$	DeepONet	132	5.21		5.46
	DeepOcKAN	108	5.29		3.70
	DeepOKKAN	156	8.27		3.29
	QR-DeepONet	482	3.40	3.60	2.73
	QR-DeepOcKAN	462	3.41	3.63	5.67
	**QR-DeepOKKAN**	496	4.29	4.27	**2.64**
$\frac{1}{1000}$	DeepONet	132	5.09		8.79
	DeepOcKAN	108	5.11		7.00
	DeepOKKAN	156	7.09		6.11
	**QR-DeepONet**	497	3.40	3.60	**5.70**
	QR-DeepOcKAN	461	3.30	3.19	6.56
	QR-DeepOKKAN	509	3.94	3.46	5.74
