# Supplementary material for: KKANs: Ku̇rková-Kolmogorov-Arnold networks and their learning dynamics
Source: Neural Netw. Author manuscript; Available in PMC 2026 May 7. (PMC13151308; doi:10.1016/j.neunet.2025.107831)
Supplement: 1 [file NIHMS2169401-supplement-1.pdf]

Appendix A. Proof of Theorem 1

Thanks to the KART/variants, we can represent any $f \in C(E^d)$ as in Eq. 4:

$$f(\mathbf{x}) = \sum_{q=0}^{m} g_q(\xi), \quad \xi := \xi_q(\mathbf{x}) = \sum_{p=1}^{d} \psi_{p,q}(x_p), \tag{A.1}$$

where $\psi_{p,q}, g_q$ are univariate continuous functions. The continuity of $\psi_{p,q}$ on the closed interval I_ψ implies that there exists $B > 0$ such that

$$\max_{p,q} \max_{x \in I_\psi} \left|\psi_{p,q}(x)\right| \le \frac{B}{d} \quad \Rightarrow \quad \xi \in [-B, B].$$

Since all g_q are uniformly continuous on $[-B, B] \subseteq I_g$, there exists $\delta > 0$ such that

$$\max_q \max_{\substack{|\xi-\eta|<\delta \\ \xi,\eta \in [-B,B]}} \left|g_q(\xi) - g_q(\eta)\right| < \frac{\varepsilon}{2(2m+1)}. \tag{A.2}$$

Using the assumption that $\mathcal{A}_{M_z}(I_z)$ is dense in $C(I_z)$, we know that there exist $\Psi_{p,q} \in \mathcal{A}_{M_\psi}(I_\psi)$ for each pair (p, q) such that

$$\max_{p,q} \max_{x \in I_\psi} \left|\psi_{p,q}(x) - \Psi_{p,q}(x)\right| < \frac{\delta}{d}, \tag{A.3}$$

and there exists $G_q \in \mathcal{A}_{M_g}(I_g)$ such that

$$\max_q \max_{\eta \in [-B,B]} \left|g_q(\eta) - G_q(\eta)\right| < \frac{\varepsilon}{2(2m+1)}. \tag{A.4}$$

Thus, we derive from (Eq. A.3) that

$$|\xi - \eta| := \Big| \sum_{p=1}^{d} \psi_{p,q}(x_p) - \sum_{p=1}^{d} \Psi_{p,q}(x_p) \Big| \le \sum_{p=1}^{d} |\psi_{p,q}(x_p) - \Psi_{p,q}(x_p)| < \delta.$$

Then, we can assemble $F \in \mathbb{K}_M^{m,d}$ using $\Psi_{p,q}, G_q$ as in (5). Consequently, using (Eqs. A.1, (A.2) and (A.4)) leads to

$$|f(\mathbf{x}) - F(\mathbf{x})| = \Big| \sum_{q=0}^{m} g_q\Big(\sum_{p=1}^{d} \psi_{p,q}(x_p) \Big) - \sum_{q=0}^{m} G_q\Big(\sum_{p=1}^{d} \Psi_{p,q}(x_p) \Big) \Big|$$

$$\le \sum_{q=0}^{m} \left|g_q(\xi) - G_q(\eta)\right| \le \sum_{q=0}^{m} \{ |g_q(\xi) - g_q(\eta)| + |g_q(\eta) - G_q(\eta)| \} < \varepsilon.$$

This completes the proof.

Appendix B. Representation models

B.1. Multilayer perceptron (MLP)

The output y of a Multilayer Perceptron (MLP) is computed through a nested formulation, where σ is the activation function, and $W^{(l)}$ and $b^{(l)}$ are the weights and biases of the l-th layer:

$$y(\mathbf{x}) = \sigma\big(W^{(L)}\sigma\big(W^{(L-1)} \ldots \sigma\big(W^{(1)}\mathbf{x} + b^{(1)}\big) \ldots + b^{(L-1)}\big) + b^{(L)}\big).$$

Here, $\mathbf{x} = (x_1, x_2, \ldots)$ is the input vector, and L is the number of layers. With sufficiently many neurons and a suitable activation function, MLPs can approximate any continuous function on compact subsets of $\mathbb{R}^n$, as guaranteed by the Universal Approximation Theorem (Hornik et al., 1989).

B.2. Kolmogorov-Arnold networks (KANs)

Kolmogorov-Arnold Networks (KANs) are inspired by the Kolmogorov-Arnold representation theorem, which states that any multivariate continuous function $f(\mathbf{x})$ on a bounded domain can be represented as a finite composition of univariate functions and addition (Liu et al., 2024c). The function $f(\mathbf{x})$ can be approximated using KANs as:

$$f(\mathbf{x}) \approx \sum_{i_{L-1}=1}^{n_{L-1}} \phi_{L-1,i_L,i_{L-1}}\left(\cdots \phi_{1,i_2,i_1}\left(\sum_{i_0=1}^{n_0} \phi_{0,i_1,i_0}(x_{i_0}) \right) \cdots \right). \tag{B.1}$$

Here, L is the number of layers, n_j is the number of neurons in the j-th layer, and $\phi_{i,j,k}$ are univariate activation functions.

In Liu et al. (2024c), $\phi(x)$ was proposed as a combination of basis functions $b(x)$ and B-splines:

$$\phi(x) = w_b b(x) + w_s \text{spline}(x), \tag{B.2}$$

where w_b, w_s, and c_n are trainable parameters. The spline function is defined as:

$$b(x) = \frac{x}{1+e^{-x}}, \quad \text{spline}(x) = \textstyle\sum_n c_n B_n(x),$$

with splines $B_n(x)$ characterized by polynomial order k and grid size g.

Recursive Chebyshev KANs (cKANs) are used as a baseline to reduce computational costs and improve stability as introduced in Shukla et al. (2024). In cKANs, the univariate functions are defined as:

$$\phi(x) = \sum_{n=1}^{D} C_n T_n(\tanh(x)), \tag{B.3}$$

where, $T_n(x)$ are Chebyshev polynomials, computed recursively:

$$T_{n+1}(x) = 2xT_n(x) - T_{n-1}(x). \tag{B.4}$$

Embedding $\tanh(x)$ ensures normalization for these polynomials.

B.3. KKANs

The KKAN framework combines a flexible inner block with customizable basis functions in its outer block, enabling high adaptability and accuracy. This formulation integrates multiple components, including polynomial embeddings and specialized basis functions. The complete model is described below.

B.3.1. Inner block (ebmlp)

The inner block computes the feature space embedding, $\Psi(x)$, for the input variables. For each input dimension x_i, we proceed as follows:

1. **Expand the input dimension:**

$$H_i^0 = [C_0, T_0(x_i), \cdots, C_{D_e} T_{D_e}(x_i)], \tag{B.5}$$

where D_e is the polynomial degree, T_j denotes the Chebyshev polynomials, and C_j are trainable parameters.

2. **Apply an MLP with L layers:** Each layer is defined as:

$$H_i^l = \sigma(W^{l-1} \cdot H_i^{l-1} + b^l), \tag{B.6}$$

where $\theta^l = \{W^l, b^l\}$ are the weights and biases of the l–th layer, and σ is the activation function.

3. **Apply a second polynomial embedding:** Expand the output of the MLP into an m-dimensional space:

$$\Psi_i(x_i) = [C_0^L, T_0(H_i^L), \cdots, C_{D_e}^L T_{D_e}(H_i^L)], \tag{B.7}$$

$$\Psi_i(x_i) \quad = [\Psi_{i,0}, \cdots, \Psi_{i,m}], \tag{B.8}$$

where C_j^L are trainable parameters.

Next, a **combination layer** aggregates the outputs along the input-dimension coordinate:

$$\xi_q = \sum_{i=1}^{d} \Psi_{q,i}(x_i), \tag{B.9}$$

where d is the input dimension.

B.3.2. Outer block (basis functions)

The outer block, denoted as $g(\cdot)$, applies a specialized basis function to the output of the inner block. The basis functions explored in this study are described as follows:

Chebyshev. For this case, the formulation follows (Eq. B.4), where the trainable parameters C_n are initialized from a normal distribution with mean 0 and variance $\frac{1}{I(D+1)}$, as described in Sidharth et al. (2024).

Legendre. This case is similar to the Chebyshev basis but with Legendre polynomials $L_n(x)$, which are computed recursively:

$$L_{n+1}(x) = \frac{(2n-1)}{n} x L_n(x) - \frac{(n-1)}{n} L_{n-1}(x).$$

The trainable parameters C_n are also initialized from a normal distribution with mean 0 and variance $\frac{1}{I(D+1)}$, as described in Sidharth et al. (2024).

Sine series. This basis, introduced in Guilhoto and Perdikaris (2024), has shown improved performance compared to the vanilla KAN. The basis functions are defined as:

$$\phi(x) = \sum_{i=1}^{D} C_i b_i,$$

where each $b_i(t)$ is given by:

$$b_i(t) = \frac{\sin(w_i t + p_i) - \mu(w_i, p_i)}{\sigma(w_i, p_i)}.$$

Here, the frequencies w_i are initialized from a standard normal distribution ($\mathcal{N}(0,1)$), and the phases p_i are initialized as 0. The mean and standard deviation are defined as:

$$\mu(w_i, p_i) = e^{-w_i^2/2} \sin(p_i), \quad \sigma(w_i, p_i) = \sqrt{\frac{1}{2} - e^{-w_i^2} \cos(p_i) - \mu(w_i, p_i)^2}.$$

Chebyshev grid. Combining previous approaches, this basis introduces a sub-expansion of the input using a linear layer within the normalization step. The basis is defined as:

$$\phi(x) = \sum_{n=1}^{D} C_n T_n\left(\sum_{i=1}^{c} \tanh(W_i x + b_i)\right),$$

where C_n are initialized as described in Sidharth et al. (2024) with $\mathcal{N}(0, \frac{1}{I(D+1)})$. The centers b_i are initialized on a grid in the range $[-0.1, 0.1]$ with c = 5, and W_i is initialized from $\mathcal{N}(0, \frac{1}{Ic})$. This approach improves expressiveness by displacing the input across multiple centers.

Radial basis functions (RBF). For this case, we follow Li (2024) and define the basis functions as:

$$\phi(x) = \sum_{n=1}^{D} C_n e^{-\frac{(x-p_n)^2}{2\sigma^2}},$$

where C_n are initialized from $\mathcal{N}\left(0, \frac{1}{I(D+1)}\right)$, and p_n are initialized from a uniform grid spanning $(-2.0, 2.0)$ with D steps. Here, D represents the number of centers, and σ is a hyperparameter controlling the spread of the basis functions.

Single radial basis functions (RBF-single). To further evaluate the versatility of our 2-block representation framework, we consider a simplified case. Here, the KART architecture (Eq. 11) is rewritten using a single outer function as follows:

$$f(x_1, \ldots, x_d) = \sum_{q=0}^{m} G\Big(\sum_{p=1}^{d} \Psi_{p,q}(x_p)\Big).$$

This formulation demonstrates that our framework can seamlessly adapt to representations with a single outer block. We implement and test this example using Radial Basis Functions (RBF), referring to it as “RBF-Single” throughout the study.

B.3.3. Full model

Finally, we combine the inner and outer blocks as follows:

$$f(x_1, \cdots, x_d) = \sum_{q=0}^{m} g_q(\xi_q). \tag{B.10}$$

Appendix C. Additional enhancements

C.1. Weight normalized modified multi-layer perceptrons

For this part, we combine the modified multi-layer perceptron (mMLP) introduced in Wang et al. (2021a) and the weight normalization proposed in Salimans and Kingma (2016). The mMLP aims to augment the efficacy of PIML by embedding the input variables x into the hidden states of the network. On the other hand, weight normalization is a reparameterization technique that accelerates convergence in PIML (Raissi et al., 2020). In particular, the inputs are encoded in a feature space by employing two distinct encoders, U and V, given by:

$$U = \sigma(x^0 W^U + b^U), \quad V = \sigma(x^0 W^V + b^V) \tag{C.1}$$

The encoders are then assimilated within each hidden layer of a conventional MLP by point-wise multiplication. Thus, each forward pass becomes:

$$\alpha^l(x) = \alpha^{l-1}(x) W^l + b^l, \quad for \;\; l \in \{1, 2, \ldots, L\} \tag{C.2}$$

$$\alpha^l(x) = \sigma(\alpha^l(x)) \tag{C.3}$$

$$\alpha^l(x) = (1 - \alpha^l(x)) \odot U + \alpha^l(x) \odot V, \tag{C.4}$$

where x is the input, α^l and W^l are the neurons and weights of layer l, σ is the activation function and $\odot$ is the element-wise product. Finally, we include WN by reparameterizing our weights as:

$$\alpha = \sigma(W \cdot x + b) \tag{C.5}$$

$$W = \frac{g}{\|\boldsymbol{v}\|_2} \boldsymbol{v}, \tag{C.6}$$

where α is the neuron output, σ is the activation function, x is the input vector, W is a weight vector, and b is the bias. As shown in (Eq. C.6), the weight vector W is redefined in terms of new trainable parameters, $\boldsymbol{v}$ (direction) and g (length). Notice that $\|W\| = g$, so this reparameterization allows us to decouple the weight's length and direction, which speeds up the model convergence. Since g is a scalar, this modification induces minimal computational overhead (Salimans & Kingma, 2016).

C.2. Weight-normalized adaptive ResNet (WNadResNet)

The Weight-Normalized Adaptive ResNet (WNadResNet) builds on the concept of adaptive residual connections proposed in Wang et al. (2024a). However, our implementation focuses on reducing computational overhead while retaining flexibility and performance. Unlike the approach in Wang et al. (2024a), which incorporated modified MLPs, we simplify the architecture to achieve comparable performance with KKANs while significantly lowering computational costs.

For each WNadResNet layer, the forward pass is computed as follows:

1. Compute the transformed feature F from the input H using a weight-normalized layer and a nonlinear activation:

$$F = \sigma(W \cdot H + b), \tag{C.7}$$

where W and b are the weight and bias parameters of the layer, and σ is the hyperbolic tangent activation (tanh).

2. Apply a second transformation G to F:

$$G = W' \cdot F + b', \tag{C.8}$$

where W' and b' are parameters of another weight-normalized layer.

3. Combine the original input H with the transformed feature G through an adaptive residual connection:

$$H' = \sigma(\alpha \cdot G + (1-\alpha) \cdot H), \tag{C.9}$$

where α is a trainable scalar that adaptively balances the contributions of G and H.

The weights W and W' are reparameterized using weight normalization (Salimans & Kingma, 2016).

By introducing the adaptive parameter α, WNadResNet allows the network to dynamically adjust the residual contribution of the input, enhancing learning flexibility. This framework is particularly effective for KKANs, enabling high performance with minimal computational overhead.

Appendix D. Deep operator network (DeepONet)

In this section, we describe the DeepONet (Lu et al., 2019) and QR-DeepONet Lee and Shin (2024) frameworks as described in Lee and Shin (2024).

D.1. Operators

Let $\Omega_X \subset \mathbb{R}^{d_x}$, $\Omega_Y \subset \mathbb{R}^{d_y}$, and $(\mathcal{X}, d_{\mathcal{X}})$ represent a metric space of functions defined over Ω_X. Additionally, let $(\mathcal{Y}, \|\cdot\|_{\mathcal{Y}})$ be a normed vector space of functions defined over Ω_Y. The operator of interest is denoted as:

$$\mathcal{G} : \mathcal{X} \ni f \mapsto \mathcal{G}[f] \in \mathcal{Y}.$$

To approximate $\mathcal{G}$, the function $f(x)$ is discretized using a suitable basis $\{\phi_i\}$:

$$f(x) = \sum_{i=1}^{\infty} \hat{f}_i \phi_i(x), \quad \hat{f}_i = \langle f, \phi_i \rangle,$$

where $\langle \cdot, \cdot \rangle$ is an appropriate inner product. The discrete representation of f is given by:

$$\boldsymbol{f} = (\hat{f}_1, \dots, \hat{f}_{m_x}),$$

where m_x is the number of sensors used for the input function f. The goal is to approximate the operator $\mathcal{G}$ using a neural network-based model $\mathcal{G}_{NN}$.

D.2. DeepONet

For $L \in \mathbb{N}$ and $\boldsymbol{n} = (n_0, n_1, \dots, n_L) \in \mathbb{N}^{L+1}$, a representation model $\mathcal{M}$ with L layers maps an input $x \in \mathbb{R}^{n_0}$ to $z^L \in \mathbb{R}^{n_L}$. Here, $\boldsymbol{n}$ specifies the network architecture (Tables F.7–F.13).

D.2.1. DeepONet structure

DeepONet architectures consist of two main components: the **branch network** and the **trunk network**.

Branch network. The branch network $\boldsymbol{c}(\cdot;\theta)$ is a vector-valued representation model with L_b layers:

$$\boldsymbol{c}(\cdot;\theta) = (c_0(\cdot;\theta), \dots, c_N(\cdot;\theta))^T,$$

where its architecture is defined as $\boldsymbol{n}_b = (m_x, n_1^{(b)}, \dots, N+1)$, and θ represents the trainable parameters.

Trunk network. The trunk network $\phi(\cdot;\mu)$ is a vector-valued representation model with L_t layers:

$$\phi(\cdot;\mu) = (1, \phi_0(\cdot;\mu))^T,$$

where $\phi_0(\cdot;\mu) = (\phi_1(\cdot;\mu), \dots, \phi_N(\cdot;\mu))$ with an architecture $(d_y, n_1^{(t)}, \dots, n_{L_t-1}^{(t)}, N)$. Here, μ represents the trainable parameters.

D.2.2. DeepONet approximation

The output of DeepONet is defined as the inner product of the branch and trunk networks:

$$O_{\text{net}}[\boldsymbol{f};\Theta](y) = \phi^T(y;\mu)\boldsymbol{c}(\boldsymbol{f};\theta),$$

which can be expanded as:

$$O_{\text{net}}[\boldsymbol{f};\Theta](y) = c_0(\boldsymbol{f};\theta) + \sum_{j=1}^{N} c_j(\boldsymbol{f};\theta)\phi_j(y;\mu).$$

Here, $\Theta = \{\mu, \theta\}$ is the set of trainable parameters of the DeepONet.

D.2.3. Training

Let $\{f_k\}_{k=1}^K$ be a set of input functions from $\mathcal{X}$ and $u_k(\cdot) = \mathcal{G}[f_k](\cdot)$ be the corresponding output functions in $\mathcal{Y}$. Let $\|\cdot\|_{\mathcal{Y}_{m_y}}$ be a discretized norm. The objective is to optimize the parameters of DeepONet by minimizing the following loss:

$$\mathcal{L}(\Theta) = \frac{1}{K}\sum_{k=1}^{K} \|O_{\text{net}}[\boldsymbol{f}_k;\Theta](\cdot) - u_k(\cdot)\|_{\mathcal{Y}_{m_y}}^p.$$

D.3. QR-DeepONet

QR-DeepONet extends the standard DeepONet framework by introducing a reparameterization of the trunk network and leveraging a QR decomposition for improved stability during training (Lee & Shin, 2024).

The functional space $\mathcal{Y}$ is defined as $L^p_\omega(\Omega_y)$, with the norm given by:

$$\|g\|_{\mathcal{Y}} = \left(\int_{\Omega_y} |g(y)|^p d\omega\right)^{1/p}, \quad \forall g \in \mathcal{Y},$$

where ω is a probability measure satisfying $\int_{\Omega_y} d\omega(y) = 1$.

For practical applications, the discrete version of this norm, computed via Monte Carlo sampling, is given by:

$$\|g\|_{\mathcal{Y}_{m_y}} = \left(\frac{1}{m_y}\sum_{i=1}^{m_y} |g(y_i)|^p \omega(y_i)\right)^{1/p}, \quad \forall g \in \mathcal{Y},$$

where $\{y_i\}_{i=1}^{m_y}$ are i.i.d. random samples from ω, and m_y is the number of output sensors.

To discretize the function $g \in \mathcal{Y}$, let:

$$\boldsymbol{g} = (g(y_1), \ldots, g(y_{m_y}))^T.$$

The training data is then represented as:

$$(\boldsymbol{f}_k, \boldsymbol{u}_k) = (f_k(x_1), \ldots, f_k(x_{m_x}), u_k(y_1), \ldots, u_k(y_{m_y})), \quad k = 1, \ldots, K.$$

D.3.1. Loss function

The QR-DeepONet loss function is defined as:

$$\mathcal{L}(\{\mu, \theta\}) = \frac{1}{K}\sum_{k=1}^{K} \frac{1}{m_y}\sum_{i=1}^{m_y} \left|\phi^T(y_i;\mu)\boldsymbol{c}(\boldsymbol{f}_k;\theta) - u_k(y_i)\right|^p.$$

This loss function can be reformulated using matrix representations for computational efficiency. Define:

- The trunk matrix:

$$\boldsymbol{\Phi}(\mu) = \begin{bmatrix} \phi^T(y_1;\mu) \\ \vdots \\ \phi^T(y_{m_y};\mu) \end{bmatrix} \in \mathbb{R}^{m_y \times (N+1)}.$$

- The branch matrix:

$$\boldsymbol{C}(\theta) = [\boldsymbol{c}(\boldsymbol{f}_1;\theta), \ldots, \boldsymbol{c}(\boldsymbol{f}_K;\theta)] \in \mathbb{R}^{(N+1)\times K}.$$

- The target output matrix:

$$\boldsymbol{U} = [\boldsymbol{u}_1, \ldots, \boldsymbol{u}_K] \in \mathbb{R}^{m_y \times K}.$$

Using these matrices, the loss function can be compactly expressed as:

$$\mathcal{L}(\{\mu, \theta\}) = \frac{1}{K m_y}\|\boldsymbol{\Phi}(\mu)\boldsymbol{C}(\theta) - \boldsymbol{U}\|^p_{p,p}.$$

D.3.2. Reparameterization of the trunk network

Let $T \in \mathbb{R}^{(N+1)\times(N+1)}$ be a trainable square matrix. The trunk network is reparameterized as:

$$\hat{\phi}(\cdot;\mu, T) = T^T\phi(\cdot;\mu).$$

The output of the network becomes:

$$O_{\text{net}}[\boldsymbol{f}](y) = \hat{\phi}^T(y;\mu, T)\boldsymbol{c}(\boldsymbol{f};\theta) = \phi^T(y;\mu)T\boldsymbol{c}(\boldsymbol{f};\theta).$$

This reparameterization modifies the original branch network $\boldsymbol{c}(\boldsymbol{f};\theta)$ into $T\boldsymbol{c}(\boldsymbol{f};\theta)$. For standard MLPs, this transformation adjusts the last layer's weights and biases:

$$T\boldsymbol{c}(\boldsymbol{f};\theta) = TW^{L_b}\sigma(z^{L_b-1}) + Tb^{L_b}.$$

D.3.3. Two-step training

To optimize QR-DeepONet, a two-step training procedure is employed:

1. **Optimize the trunk network.** First, the trunk network parameters μ and an auxiliary matrix $A \in \mathbb{R}^{(N+1)\times K}$ are optimized by solving:

   $$\min_{\mu, A} \|\boldsymbol{\Phi}(\mu)A - \boldsymbol{U}\|^p_{p,p}. \tag{D.1}$$

   After optimization, perform a QR decomposition of $\boldsymbol{\Phi}(\mu^*)$:

   $$\boldsymbol{\Phi}(\mu^*) = Q^* R^*,$$

   where Q^* is orthogonal, and R^* is upper triangular. Set $T^* = (R^*)^{-1}$.
2. **Optimize the branch network.** Using the precomputed R^* and A^*, optimize the branch network parameters θ by solving:

   $$\min_{\theta} \|\boldsymbol{C}(\theta) - R^* A^*\|. \tag{D.2}$$

This two-step process decouples the optimization of the trunk and branch networks, leveraging the QR decomposition to ensure numerical stability and effective training.

Appendix E. self-scaled Residual-based-attention Algorithm

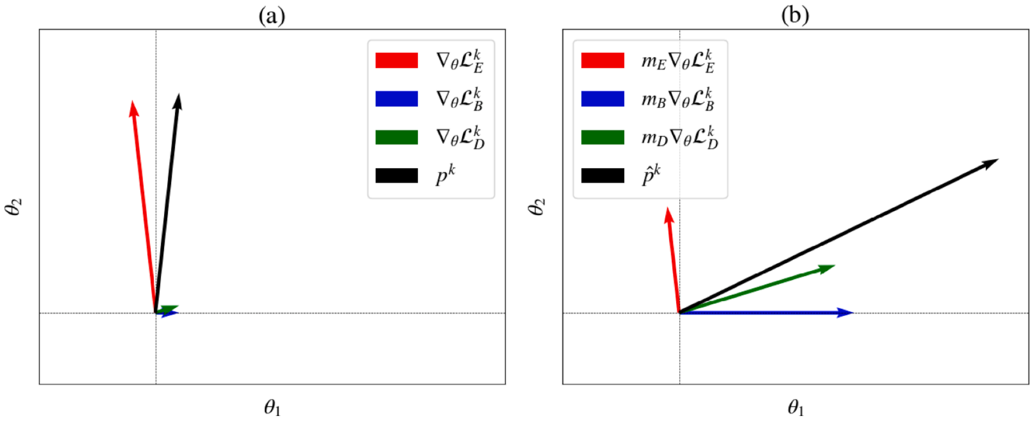

Fig. E.16. Visualization of loss gradients as directional vectors for an idealized model parameterized by $\theta = \{\theta_1, \theta_2\}$. (a) When the magnitude of one component dominates, the update direction p^k becomes imbalanced, leading to poor convergence. (b) By applying appropriate global weights, the update direction is scaled, resulting in a balanced update $\hat{p}^k$ that accommodates all directions.

Algorithm 1 self-scaled Residual-based attention.

Input: Representation model (i.e., MLP, cKAN, or KKAN): $\mathcal{M}$
Training points: X_D and/or X_B,X_E
Optimizer parameters: lr for MLP or cKAN and lr_Ψ, lr_G for KKAN.
ssRBA parameters: η, λ_{max_0},ν, c,λ_{cap}, α, m_E, γ_g
Number of iterations per stage: N_{stage}
Total number of training of iterations: N_{train}
Output: Optimized network parameters θ

Initialize the network parameters: θ for MLP or $\theta = \{\theta_\psi, \theta_g\}$ for KKAN
Initialize RBA: $\lambda^0_{\alpha,i} = 0.1\lambda_{max0}\ \forall \alpha, i$ with $\alpha, i = \{B, D, E\}$
for $k < N_{train}$ **do**
  Update maximum RBA upper bound: $\lambda_{\max} = \max(\lambda_{max0} + k/N_{stage}, \lambda_{cap})$
  Update decay rate: $\gamma^k = 1 - \eta/\lambda_{\max}$
  for each $\alpha \in \{B, D, E\}$ **do**
    Compute the sampling p.d.f.s: $p_\alpha^{(k)} \leftarrow (\lambda_\alpha^{(k)})^\nu / \mathbb{E}[(\lambda_\alpha^{(k)})^\nu] + c$
    Sample bs points from X_α: $X_\alpha^k \sim p_\alpha^{(k)}$
    Compute network prediction: $u_{\alpha,i} \leftarrow \mathcal{M}(\theta, x^k_{\alpha,i})$ for $\forall x_{\alpha,i} \in X^k_\alpha$
    Compute residuals: $r_{\alpha,i}$ using $u_{\alpha,i}$ and equations (18), (14) or (17).
    Update RBA: $\lambda^{k+1}_{\alpha,i} \leftarrow \gamma^k \lambda^k_{\alpha,i} + \eta \| r_{\alpha,i}| / \max_j |r_{\alpha,j}|$
    Compute loss term: $\mathcal{L}^k_\alpha = \langle (\lambda^k_{\alpha,i} r^k_{\alpha,i})^2 \rangle$
    Compute gradient:$\nabla_\theta \mathcal{L}^k_\alpha$
    Compute the average gradient magnitude :$\|\nabla_\theta \bar{\mathcal{L}}^k_\alpha\| = \gamma_g \|\nabla_\theta \bar{\mathcal{L}}^{k-1}_\alpha\| + (1-\gamma_g)\|\nabla_\theta \mathcal{L}^{k-1}_\alpha\|$
  end for
  Update data global weight: $m^k_D = \alpha m^{k-1}_D + (1-\alpha) m_E \|\nabla_\theta \bar{\mathcal{L}_E}^k\| / \|\nabla_\theta \bar{\mathcal{L}_D}^k\|$
  Update weight: $m^k_B = \alpha m^{k-1}_B + (1-\alpha) m_E \|\nabla_\theta \bar{\mathcal{L}_E}^k\| / \|\nabla_\theta \bar{\mathcal{L}_B}^k\|$
  Define total update direction: $p^k \leftarrow -m_E \nabla_\theta \mathcal{L}^k_E - m^k_B \nabla_\theta \mathcal{L}^k_B - m^k_D \nabla_\theta \mathcal{L}^k_D$
  if $\mathcal{M}$ = KKAN **then**
    Update inner-block parameters: $\theta^{k+1}_\psi \leftarrow \theta^k_\psi - lr^k_\psi p^k_\psi$
    Update outer-block parameters: $\theta^{k+1}_g \leftarrow \theta^k_g - lr^k_g p^k_g$
  else
    Update parameters: $\theta^{k+1} \leftarrow \theta^k - lr^k p^k$
  end if
end for

Appendix F. Implementation details

All models were trained using the ADAM optimizer (Kingma & Ba, 2014) with an exponential learning rate decay. The implementation was developed in JAX 0.4.29, with all training, evaluation, and visualization conducted in single precision on an NVIDIA GeForce RTX 3090 GPU with 24GB of VRAM.

For KKAN, the inner blocks were constructed using enhanced basis MLPs (ebMLPs), and the outer blocks utilized suitable basis functions. The activation function for both MLPs and KKANs was the hyperbolic tangent, $\tanh(\cdot)$. Weight normalization (Salimans & Kingma, 2016) was applied to all models to stabilize training and improve convergence. For cKAN models, we follow Sidharth et al. (2024) and initialize our parameters using a truncated normal distribution with a standard deviation given by $\sigma = \frac{1}{I(D+1)}$ where I is the input dimension and D is the polynomial degree. For KKAN models, we initialize our basis functions as described in Appendix B.3.2.

Further specific implementation details for each experiment are described in the following sections.

F.1. Function approximation

F.1.1. Discontinuous

Table F.7
Implementation details for discontinuous function approximation. I denotes the input dimension. For KKAN, there are two polynomial degrees: D for the outer blocks and D_e for the ebMLP used in the inner blocks. The initialization details for the outer blocks are described in the preceding section.

Hyperparameter	MLP	cKAN	KKAN
N. of training iterations	2e5	2e5	2e5
Number of hidden layers N	5	4	4
Hidden layer dimension H	100	40	32
Number of KKAN features m			32
Polynomial degree D		7	7
ebMLP Polynomial degree D_e			7
Initialization	Glorot (Glorot & Bengio, 2010)	$\mathcal{N}\left(0, \frac{1}{I(D+1)}\right)$ (Sidharth et al., 2024)	Glorot (Glorot & Bengio, 2010)
Learning rate lr	1e-3	2e-4	
KKAN: Learning rate inner lr_Ψ			1e-3
KKAN: Learning rate outer lr_G			2e-4
lr−Decay rate	0.9	0.9	0.9
lr−Decay step	5000	5000	5000
ssRBA:γ	0.999	0.999	0.999
ssRBA:η	0.01	0.01	0.01
ssRBA:λ_{max0}	10	10	10
ssRBA:λ_{cap}	20	20	20
ssRBA:N_{stage}	50000	50000	50000
ssRBA:m_E	1.0	1.0	1.0

F.1.2. Smooth

Table F.8
Implementation details for smooth function approximation. I denotes the input dimension. For KKAN, there are two polynomial degrees: D for the outer blocks and D_e for the ebMLP used in the inner blocks. The initialization details for the outer blocks are described in the preceding section.

Hyperparameter	MLP	cKAN	KKAN
N. of training iterations	2e5	2e5	2e5
Number of hidden layers N	5	4	4
Hidden layer dimension H	100	40	32
Number of KKAN features m			32
Polynomial degree D		5	15
ebMLP Polynomial degree D_e			15
Initialization	Glorot (Glorot & Bengio, 2010)	$\mathcal{N}\left(0, \frac{1}{I(D+1)}\right)$ (Sidharth et al., 2024)	Glorot (Glorot & Bengio, 2010)
Learning rate lr	1e-3	2e-4	
KKAN: Learning rate inner lr_Ψ			1e-3
KKAN: Learning rate outer lr_G			2e-4
lr−Decay rate	0.9	0.9	0.9
lr−Decay step	5000	5000	5000
ssRBA:γ	0.999	0.999	0.999
ssRBA:η	0.01	0.01	0.01
ssRBA:λ_{max0}	10	10	10
ssRBA:λ_{cap}	20	20	20
ssRBA:N_{stage}	50000	50000	50000
ssRBA:m_E	1.0	1.0	1.0

F.2. Physics-informed machine learning

F.2.1. Allen-Cahn equation

Table F.9
Implementation details for solving the Allen-Cahn Equation part (a). KKAN models include features of size $m = 64$, with polynomial degrees D for the outer blocks and D_e for the ebMLP inner blocks. Initialization strategies are Glorot and Bengio (2010) for MLPs, Gaussian-based for cKAN, and uniform for KKAN as detailed in the table. Learning rates (lr) and decay parameters are specified for each architecture.

Hyperparameter	MLP	cKAN	KKAN
N. of training iterations	3e5	3e5	3e5
Number of hidden layers N	6	4	4
Hidden layer dimension H	64	32	32
Number of KKAN features m			64
Polynomial degree D		5	9
ebMLP Polynomial degree D_e			2
Initialization	Glorot (Glorot & Bengio, 2010)	$\mathcal{N}\left(0, \frac{1}{I(D+1)}\right)$ (Sidharth et al., 2024)	$U\left(-\sqrt{\frac{3}{I}}, \sqrt{\frac{3}{I}}\right)$
Learning rate lr	1e-3	2e-4	
KKAN: Learning rate inner lr_Ψ			1e-3
KKAN: Learning rate outer lr_G			2e-4
$lr-$Decay rate	0.9	0.9	0.9
$lr-$Decay step	5000	5000	5000
Batch size	1e4	1e4	1e4

Table F.10
Implementation details for solving the Allen-Cahn Equation part (b). WNmMLP refers to the weight-normalized modified MLP architecture (Salimans & Kingma, 2016; Wang et al., 2021a). Fourier feature embeddings are used with a degree of 10 across all models. For KKAN, the architecture includes features of size $m = 64$, with polynomial degrees D and D_e for the outer blocks and ebMLP inner blocks, respectively. Initialization strategies are Glorot and Bengio (2010) for MLPs, Gaussian-based for cKAN, and uniform for KKAN, as described in the table.

Hyperparameter	MLP	cKAN	KKAN
N. of training iterations	3e5	3e5	3e5
Number of hidden layers N	6	4	4
Hidden layer dimension H	64	32	32
Fourier Feature embedding (Wang et al., 2021c) degree	10	10	10
Architecture enhancement	WNmMLP		WNmMLP
Number of KKAN features m			64
Polynomial degree D		5	9
ebMLP Polynomial degree D_e			2
Initialization	Glorot (Glorot & Bengio, 2010)	$\mathcal{N}\left(0, \frac{1}{I(D+1)}\right)$ (Sidharth et al., 2024)	$U\left(-\sqrt{\frac{3}{I}}, \sqrt{\frac{3}{I}}\right)$
Learning rate lr	1e-3	2e-4	
KKAN: Learning rate inner lr_Ψ			1e-3
KKAN: Learning rate outer lr_G			2e-4
$lr-$Decay rate	0.9	0.9	0.9
$lr-$Decay step	5000	5000	5000
Batch size	1e4	1e4	1e4
ssRBA-R:γ	0.999	0.999	0.999
ssRBA-R:η	0.01	0.01	0.01
ssRBA-R:λ_{max0}	10	10	10
ssRBA-R:λ_{cap}	20	20	20
ssRBA-R:γ_g	0.99	0.99	0.99
ssRBA-R:α	0.99975	0.99975	0.99975
ssRBA-R:ν	2.0	2.0	2.0
ssRBA-R:c	0.5	0.5	0.5
ssRBA-R:N_{stage}	50000	50000	50000
ssRBA-R:m_E	1.0	1.0	1.0

Table F.11
Implementation details for solving the Allen-Cahn Equation part (c). WNmMLP refers to the weight-normalized modified MLP architecture (Salimans & Kingma, 2016; Wang et al., 2021a), while WNadResNet refers to the weight-normalized adaptive residual network (see Appendix C.2). Fourier feature embeddings with a degree of 10 are used across all models. For KKAN, the architecture includes features of size $m = 64$, with polynomial degrees D for the outer blocks and D_e for the ebMLP inner blocks. Initialization strategies are Glorot and Bengio (2010) for MLPs and KKANs, and Gaussian-based for cKANs. Learning rates (lr), decay rates, and ssRBA parameters are listed in the table.

Hyperparameter	MLP	cKAN	KKAN
N. of training iterations	3e5	3e5	3e5
Number of hidden layers N	6	5	4
Hidden layer dimension H	128	64	64
Fourier Feature embedding (Wang et al., 2021c) degree	10	10	10
Architecture enhancement	WNmMLP		WNadResNet
Number of KKAN features m			64
Polynomial degree D		5	5
ebMLP Polynomial degree D_e			7
Initialization	Glorot (Glorot & Bengio, 2010)	$\mathcal{N}\left(0, \frac{1}{I(D+1)}\right)$ (Sidharth et al., 2024)	Glorot (Glorot & Bengio, 2010)
Learning rate lr	1e-3	2e-4	
KKAN: Learning rate inner lr_Ψ			1e-3
KKAN: Learning rate outer lr_G			2e-4
lr−Decay rate	0.9	0.9	0.9
lr−Decay step	5000	5000	5000
ssRBA:γ	0.999	0.999	0.999
ssRBA:η	0.01	0.01	0.01
ssRBA:λ_{max0}	10	10	10
ssRBA:λ_{cap}	20	20	20
ssRBA:γ_g	0.99	0.99	0.99
ssRBA:α	0.99975	0.99975	0.99975
ssRBA:N_{stage}	50000	50000	50000
ssRBA:m_E	1.0	1.0	1.0

F.3. Operator learning

F.3.1. Burgers equation

Table F.12
Implementation details for the Burgers equation using the DeepONet framework. The embedding dimension represents the number of neurons in the last layer of the branch and trunk networks. KKAN models include features of size $m = 32$, with polynomial degrees D for the outer blocks and D_e for the ebMLP inner blocks. Initialization strategies are Glorot and Bengio (2010) for MLPs and KKANs, and Gaussian-based for cKANs. Learning rates (lr), decay rates, and other hyperparameters are detailed in the table. WNadResNet refers to the weight-normalized adaptive residual network used in KKANs for enhanced learning performance.

Hyperparameter	MLP	cKAN	KKAN
N. of training iterations	4e5	4e5	4e5
Number of hidden layers N	6	5	5
Hidden layer dimension H	100	32	32
Embedding dimension	100	100	100
Number of KKAN features m			32
Polynomial degree D		5	5
ebMLP Polynomial degree D_e			5
Architecture enhancement			WNadResNet
Initialization	Glorot (Glorot & Bengio, 2010)	$\mathcal{N}\left(0, \frac{1}{I(D+1)}\right)$ (Sidharth et al., 2024)	(Glorot & Bengio, 2010)
Learning rate lr	1e-3	3e-4	
KKAN: Learning rate inner lr_Ψ			1e-3
KKAN: Learning rate outer lr_G			1e-3
lr−Decay rate	0.9	0.9	0.99
lr−Decay step	2500	2500	5000

Table F.13
Implementation details for the Burgers equation using the QR-DeepONet framework. The embedding dimension refers to the number of neurons in the last layer of the branch and trunk networks. The number of parameters is increased due to the trainable matrix A, which, in this case, has 3500×3500 parameters. KKAN models include features of size $m = 32$, with polynomial degrees D for the outer blocks and D_e for the ebMLP inner blocks. Initialization strategies are Glorot and Bengio (2010) for MLPs and KKANs, and Gaussian-based for cKANs. Learning rates (lr) and decay parameters are detailed in the table. WNadResNet refers to the weight-normalized adaptive residual network used in KKANs.

Hyperparameter	MLP	cKAN	KKAN
N. of training iterations -trunk	2e5	2e5	2e5
N. of training iterations- branch	4e5	4e5	4e5
Number of hidden layers N	6	5	5
Hidden layer dimension H	100	32	32
Embedding dimension	100	100	100
Number of KKAN features m			32
Polynomial degree D		5	5
ebMLP Polynomial degree D_e			5
Architecture enhancement			WNadResNet
Initialization	Glorot (Glorot & Bengio, 2010)	$\mathcal{N}\left(0, \frac{1}{I(D+1)}\right)$ (Sidharth et al., 2024)	(Glorot & Bengio, 2010)
Learning rate lr	1e-3	3e-4	
KKAN: Learning rate inner lr_Ψ			1e-3
KKAN: Learning rate outer lr_G			1e-3
lr–Decay rate	0.9	0.9	0.99
lr–Decay step	2500	2500	5000

Appendix G. Additional results

G.1. Function approximation

G.1.1. Discontinuous

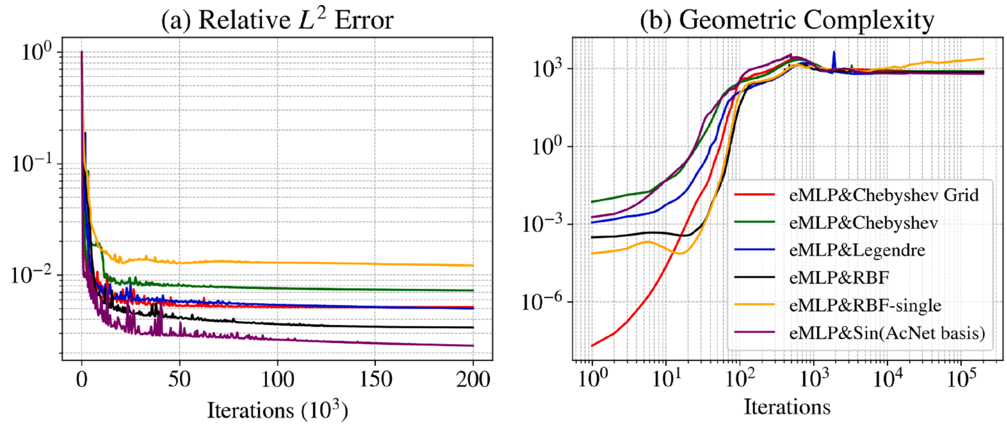

Fig. G.17. Results for the highly discontinuous function for different basis functions. (a) Relative L^2 error convergence on the testing dataset, evaluated on a uniform 256×256 mesh. The best-performing model is obtained using the sin-series basis introduced in Guilhoto and Perdikaris (2024). (b) Geometric complexity evolution during training. All models converge to the same complexity except for RBF-Single, which is higher, indicating that it is possibly over-fitting.

G.1.2. Smooth

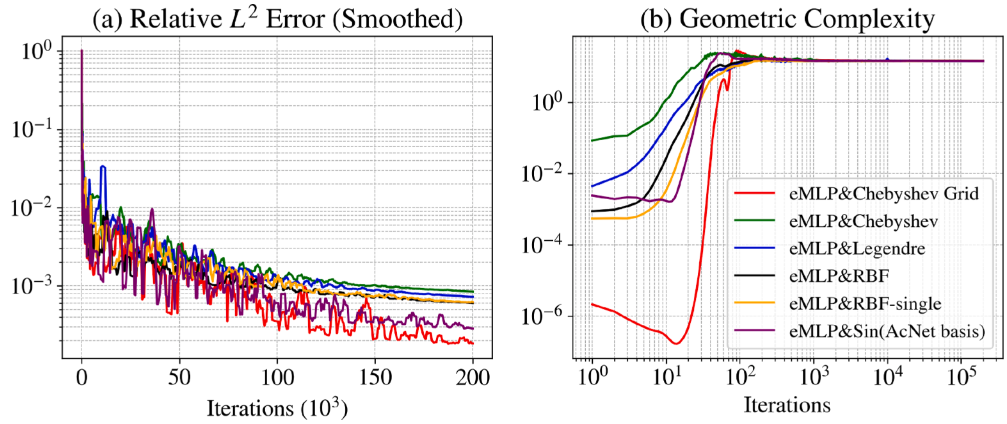

Fig. G.18. Results for the highly discontinuous function for different basis functions. (a) Relative L^2 error convergence on the testing dataset, evaluated on a uniform 256×256 mesh. The best-performing model is obtained using the sin-series basis introduced in Guilhoto and Perdikaris (2024). (b) Geometric complexity evolution during training.

G.2. Physics-informed machine learning

G.2.1. Allen cahn

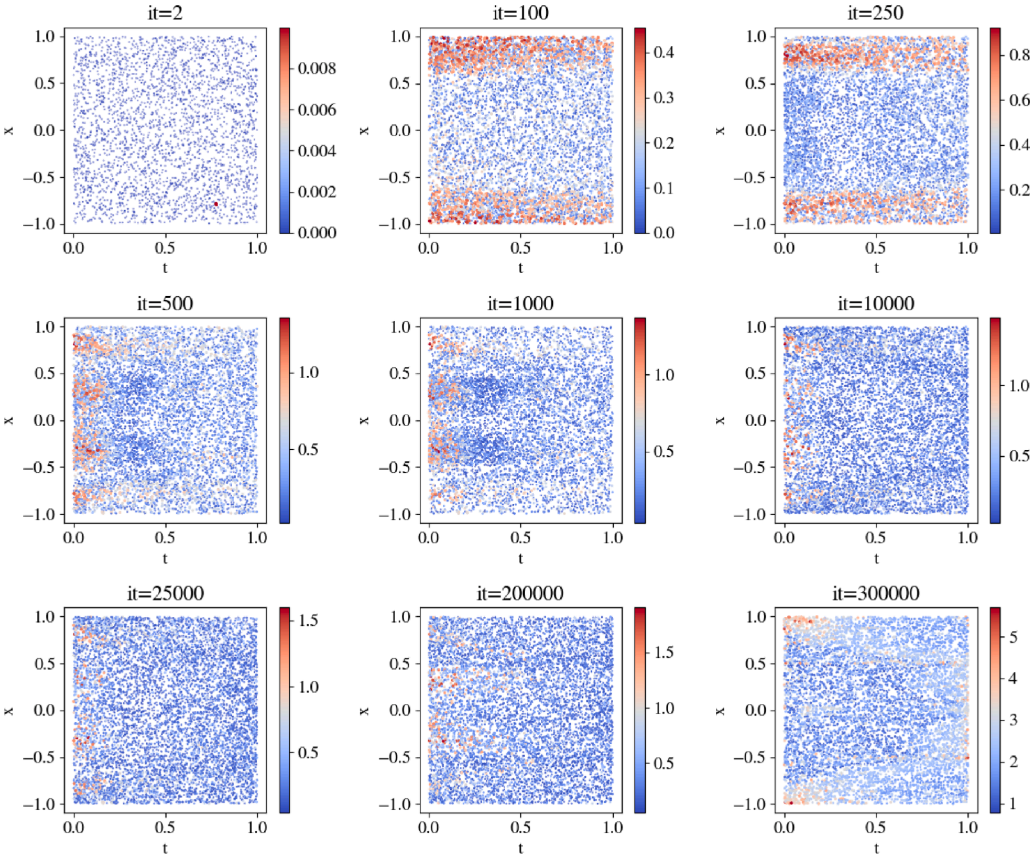

Fig. G.19. ssRBA-Rweight Evolution for the best performing KKAN model for Allen-Cahn Equation.

ssRBA-R parameter sensitivity analysis. This section analyzes the sensitivity of the “ssRBA-R” method to its primary hyperparameters. We first investigate the effect of scheduling the attention upper bound, $\lambda_{\max}$, by fixing $\eta = 0.01$. As described in Section 3.5, the local multipliers $\lambda_{\alpha,i}^{(k)}$ are bounded

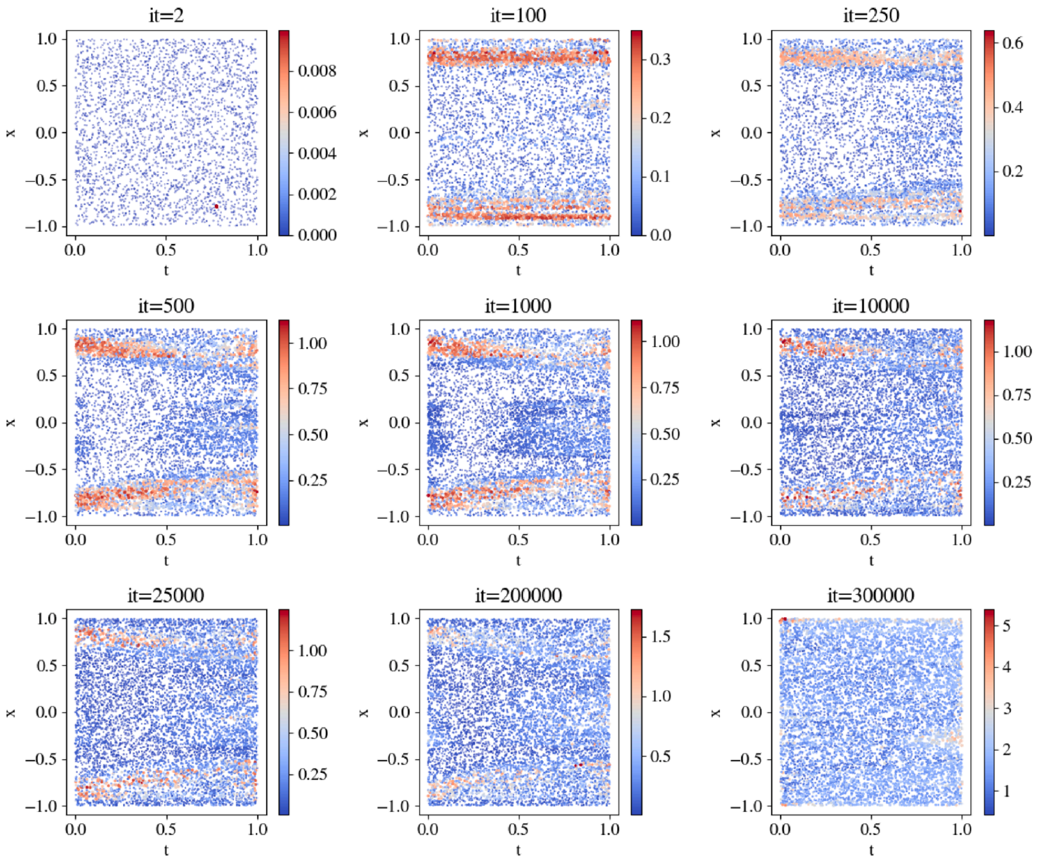

Fig. G.20. ssRBA-R weight Evolution for the best performing MLP for Allen-Cahn Equation.

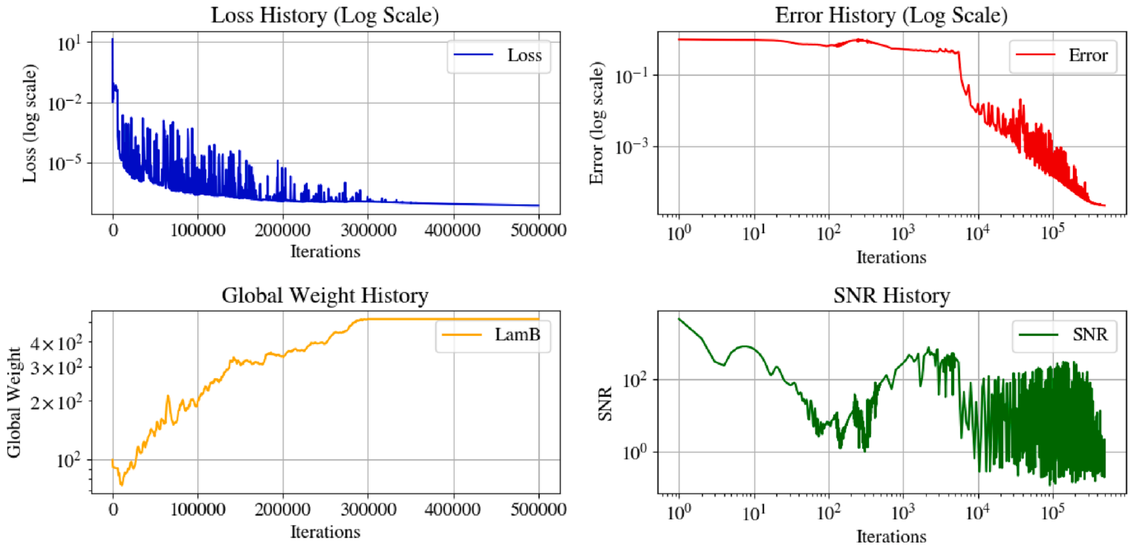

Fig. G.21. Loss, global weight, relative L^2 and SNR convergence history for the best performing KKAN + ssRBA model.

in the range $[0, \lambda_{max}]$. And we proposed increasing the λ_{max} to achieve a high SNR. Therefore, we investigate five schedules where this upper bound is increased in a stepwise manner from a starting to an ending value over the training duration, as shown in Fig. G.23 (top row).

The results reveal a non-monotonic relationship between the chosen schedule and the final accuracy. We observe that a schedule with too low a bound, such as [1,10], is suboptimal, achieving a final relative L^2 error of 1.3×10^{-4}. Increasing the range leads to improved performance, with the schedules in the intermediate range of [5,15], [10,20], and [15,25] achieving the best results, all converging to a similar error of approximately 5.5×10^{-5}. However, if the schedule is too aggressive, as in the [20,30] case, the performance again deteriorates slightly, suggesting an optimal range exists.

The overall robustness of the method across these varied schedules can be attributed to the self-scaling global weights. While a more aggressive λ_{max} schedule leads to larger weighted PDE residuals, the gradient balancing mechanism automatically compensates by increasing the boundary weight, m_B as described in Eq. (28). This effect is empirically verified in Fig. G.23 (top right). This dynamic ensures that the PDE and bound-

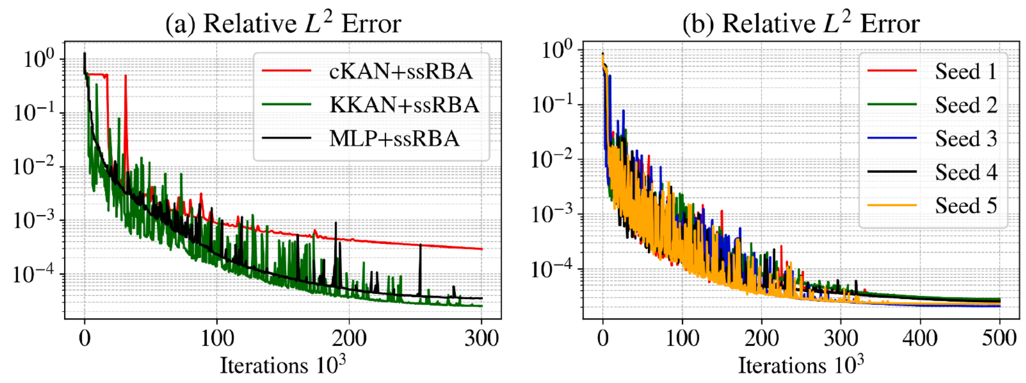

Fig. G.22. Results for solving the Allen-Cahn Equation. (a) Relative L^2 error convergence for models trained with ssRBA and full-batch training over 300,000 ADAM iterations. (b) Relative L^2 error convergence for KKAN + ssRBA models initialized with five different seeds and trained for 500,000 iterations, demonstrating robustness to initialization.

ary loss gradients maintain a comparable magnitude, which aim to promote a balanced and effective update direction regardless of the specific hyperparameters.

For the second part of this analysis, we fix the schedule to $\lambda_{\max}$: $15 \to 20$ and test four different learning rates, $\eta \in \{0.001, 0.01, 0.1, 1.0\}$. As shown in Fig. G.23 (bottom row), the results indicate similar trends, with optimal performance achieved for $\eta = 0.01$. We also note that the learning rate $\eta = 1.0$ leads to the worst performance, with a relative L^2 error of 1.2×10^{-4}. Nevertheless, as in the previous case, the results are comparable. We also note that since the schedule for $\lambda_{\max}$ is identical, the boundary weight m_B converges to the same value.

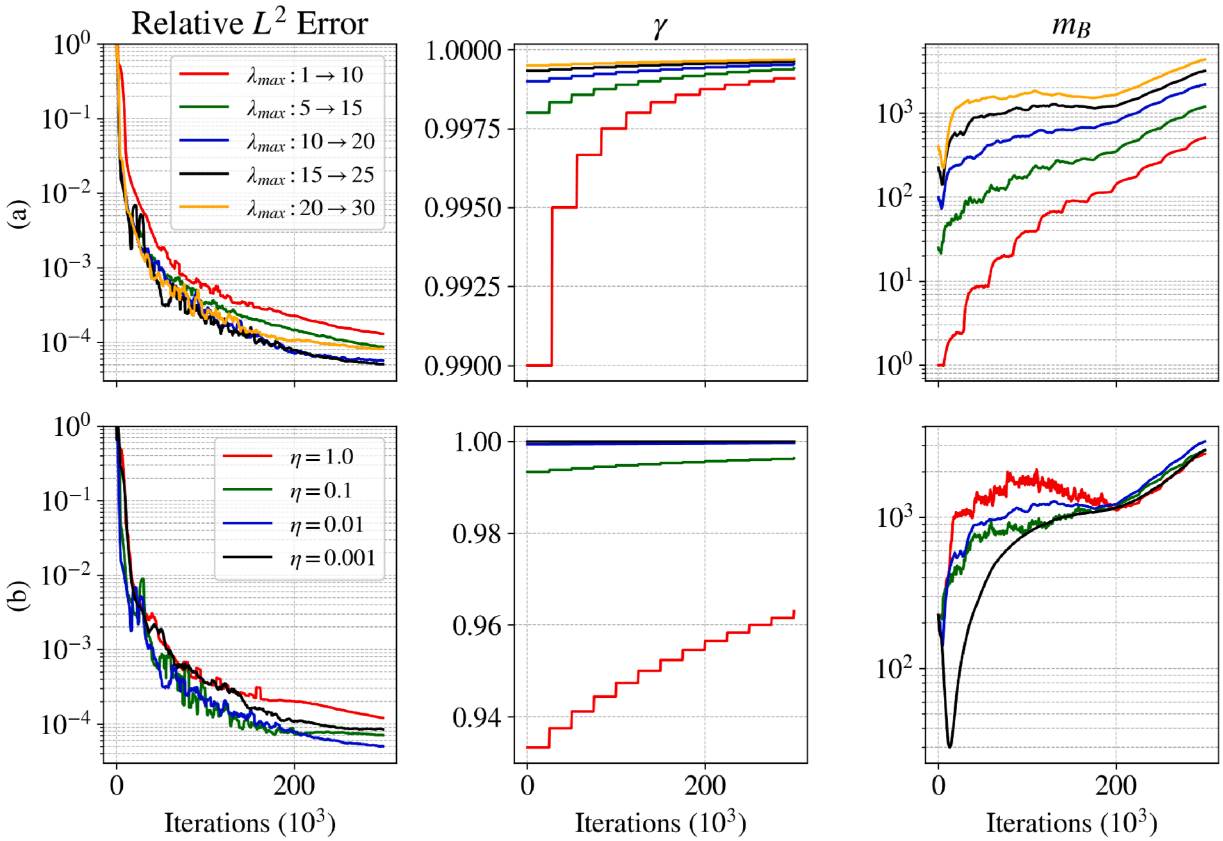

Fig. G.23. Sensitivity analysis of the ssRBA-R hyperparameters for the Allen-Cahn equation. (a) The effect of varying the stepwise scheduling of the upper bound $\lambda_{\max}$, with $\eta = 0.01$ fixed. The plots show a non-monotonic relationship between the schedule and the final accuracy (left), with an optimal performance range existing for intermediate schedules (e.g., [10,20]). More aggressive schedules for $\lambda_{\max}$ result in a higher γ (middle) and induce a larger balancing weight m_B (right). (b) The effect of varying the learning rate η, with the $\lambda_{\max}$ schedule fixed to an optimal range. The results show that $\eta = 0.01$ achieves the best performance. Since the schedule for $\lambda_{\max}$ is identical in these tests, the boundary weight m_B converges to the same value.

G.3. Operator learning

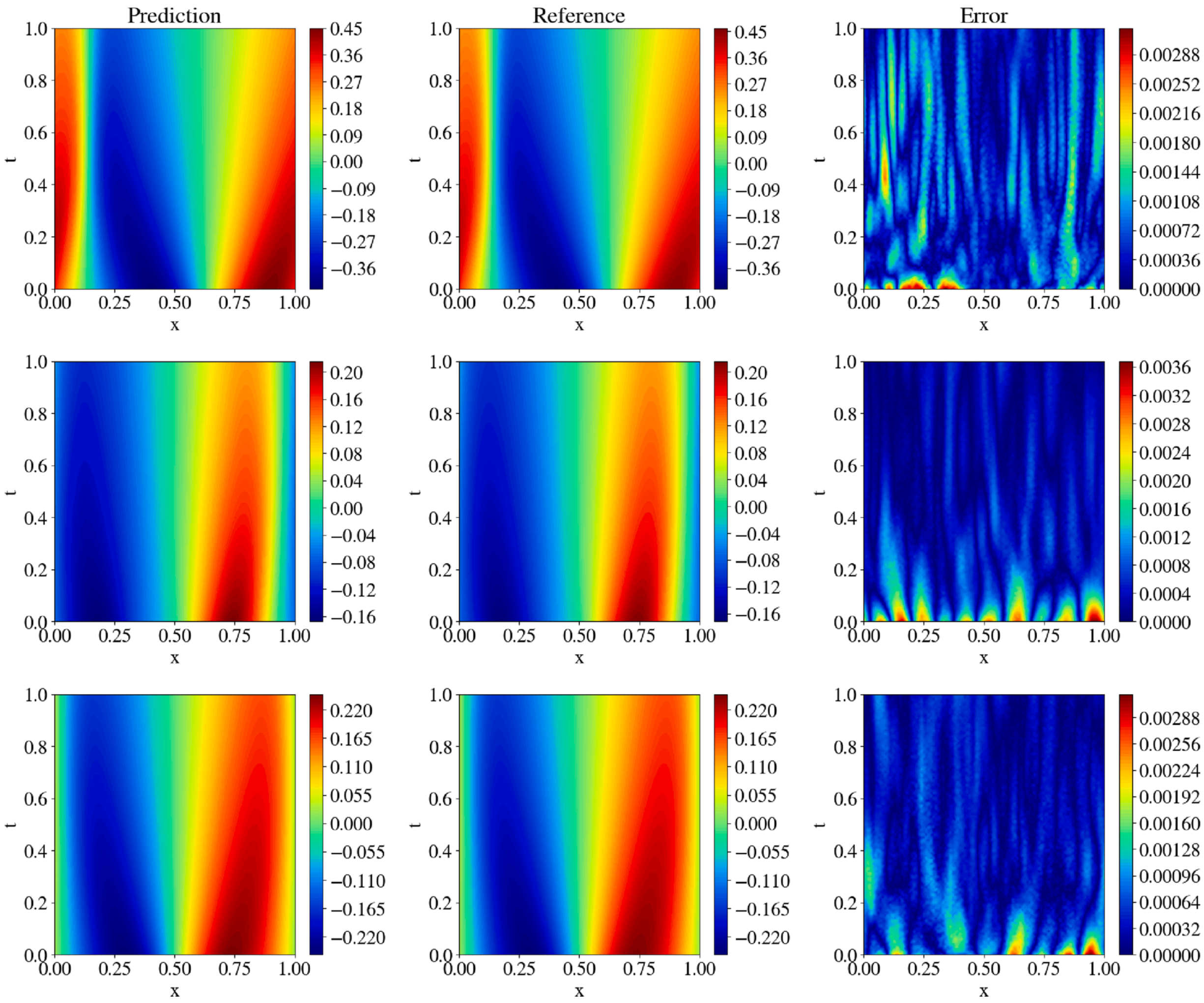

Fig. G.24. QR-DeepOKKAN predictions for three different initial conditions from the testing dataset for Burgers' Equation with $\nu = (1/100)$. The corresponding relative L^2 errors are: (top row) 2.92×10^{-3}, (middle row) 6.36×10^{-3}, and (bottom row) 3.69×10^{-3}.

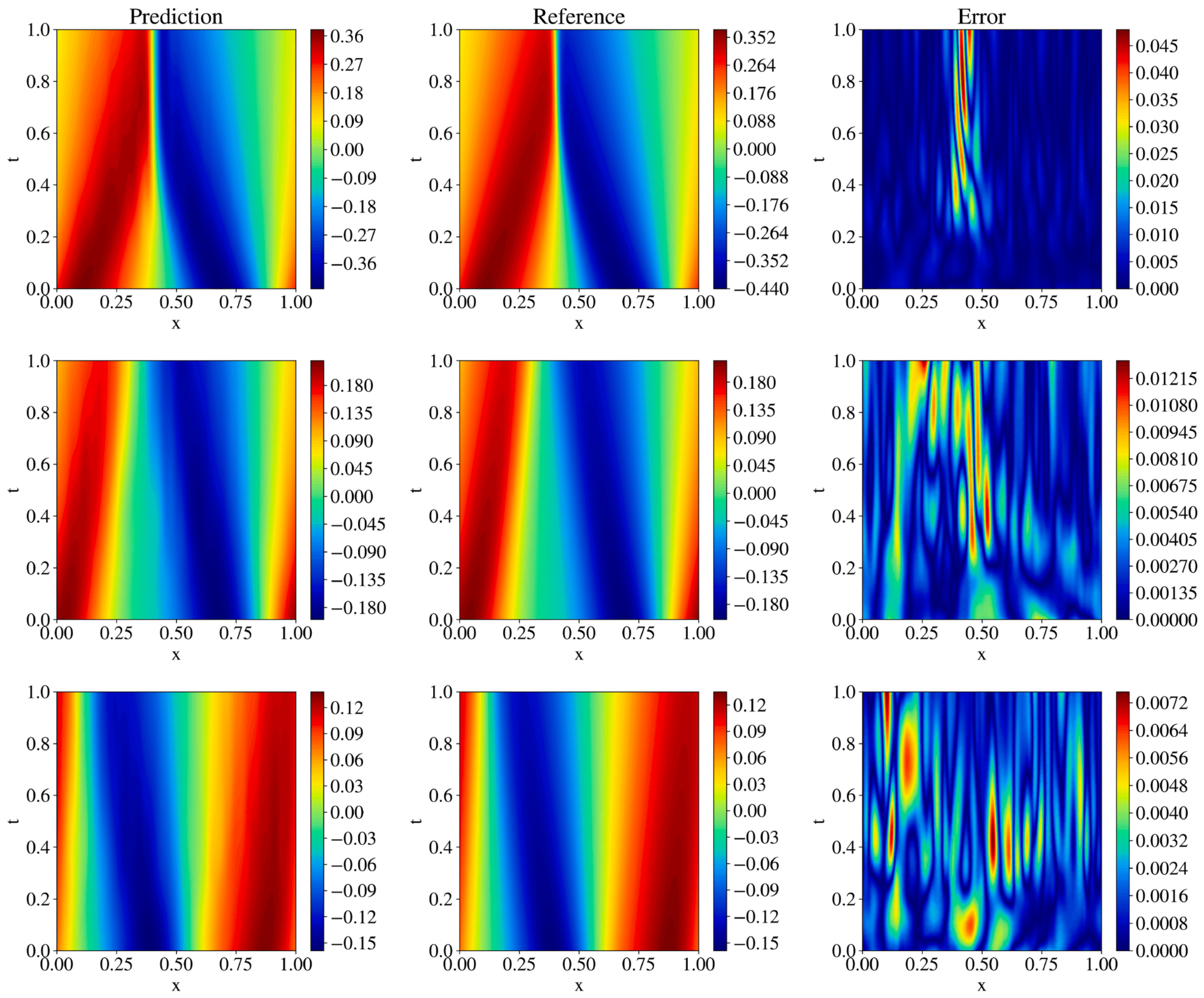

Fig. G.25. QR-DeepOKKAN predictions for three different initial conditions from the testing dataset for Burgers' Equation with $\nu = (1/100\pi)$. The corresponding relative L^2 errors are: (top row) 2.30×10^{-2}, (middle row) 2.03×10^{-2}, and (bottom row) 1.52×10^{-2}.
